# Generation of Alkyl Radicals: From the Tyranny of Tin to the Photon Democracy

**DOI:** 10.1021/acs.chemrev.0c00278

**Published:** 2020-08-06

**Authors:** Stefano Crespi, Maurizio Fagnoni

**Affiliations:** † Stratingh Institute for Chemistry, Center for Systems Chemistry University of Groningen, Nijenborgh 4, 9747 AG Groningen, The Netherlands; ‡ PhotoGreen Lab, Department of Chemistry, V. Le Taramelli 10, 27100 Pavia, Italy

## Abstract

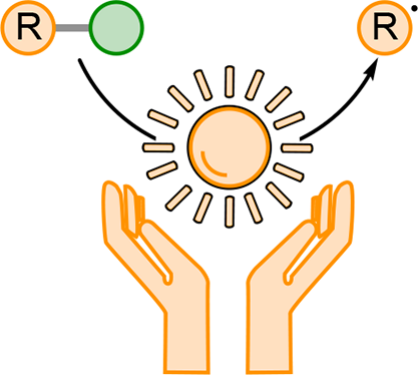

Alkyl radicals are key intermediates in organic synthesis. Their classic generation from alkyl halides has a severe drawback due to the employment of toxic tin hydrides to the point that “flight from the tyranny of tin” in radical processes was considered for a long time an unavoidable issue. This review summarizes the main alternative approaches for the generation of unstabilized alkyl radicals, using photons as traceless promoters. The recent development in photochemical and photocatalyzed processes enabled the discovery of a plethora of new alkyl radical precursors, opening the world of radical chemistry to a broader community, thus allowing a new era of photon democracy.

## 1 Introduction

Among all the open-shell species, carbon-centered radicals are intriguing neutral intermediates that find extensive use in organic synthesis, despite the initial distrust about their possible application.^1−5^ In particular, the generation of unstabilized alkyl radicals under mild conditions granting the controlled and selective outcome of the ensuing reactions has been a challenge for many years. The first and more obvious way to form such species is the homolytic cleavage of a labile C–X bond; alkyl halides appeared as the ideal choice in this respect. The real breakthrough in radical chemistry was the discovery of Bu_3_SnH to promote radical chain reactions as reported about 60 years ago in the reduction of bromocyclohexane.^6^ Reduction of an organotin halide by lithium aluminum hydride formed the reactive tin hydride in solution. In subsequent modifications of the protocol, both sodium borohydride^7^ and sodium cyanoborohydride^8^ acted as effective reducing agents. Alkyl radicals generated via tin chemistry were then used for C–C bond formation mainly via the addition to (electron-poor) olefins, the well-known Giese reaction,^9−12^ an evolution of the original process which made use of organomercury compounds.^12,13^

**Figure 1:**
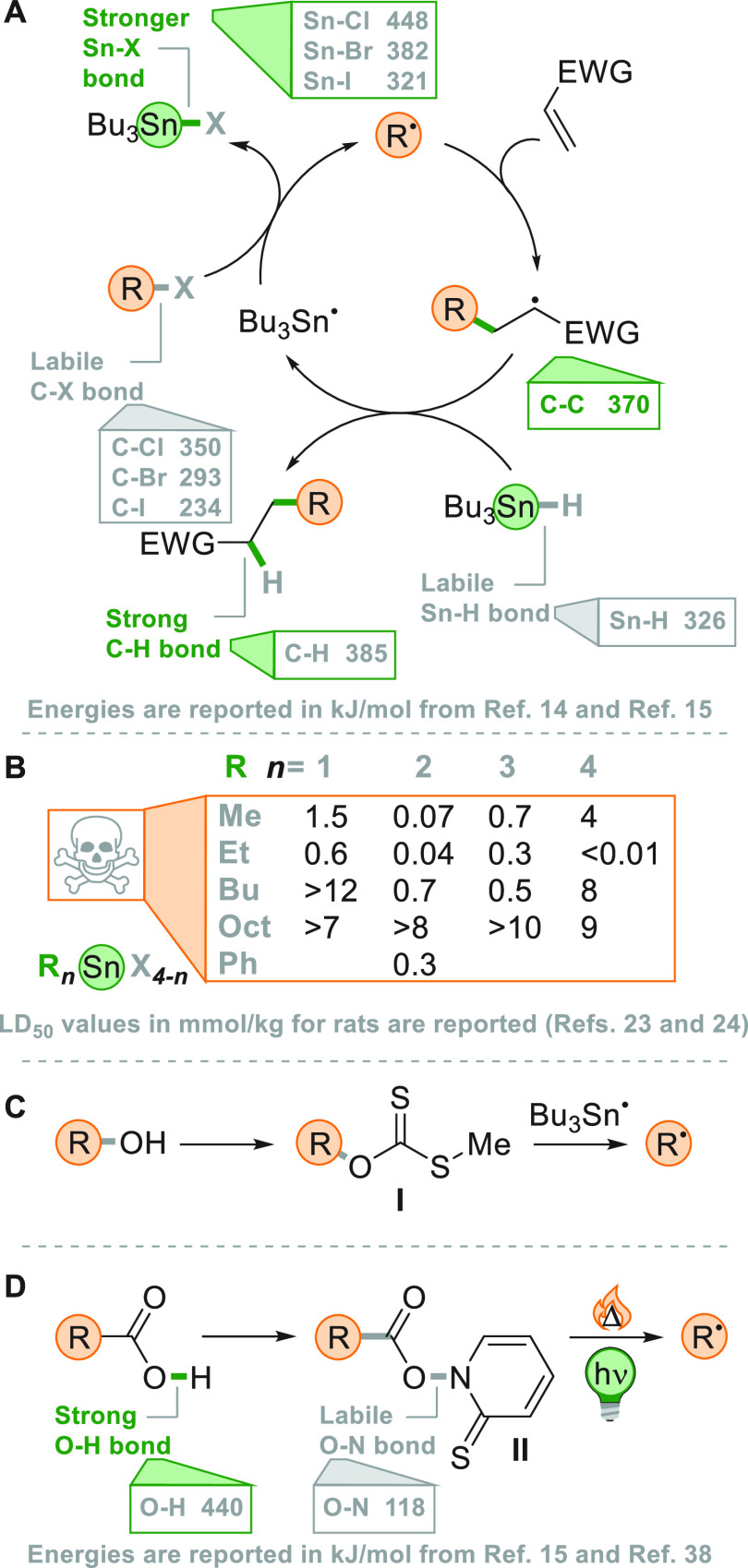
(A) Thermal generation of radicals from alkyl halides in the Giese reaction. (B) LD_50_ values for selected organotin compounds. (C) Thermal generation of radicals from alcohols via xanthates (**I**). (D) Thermal and photochemical generation of radicals from carboxylic acids via Barton esters (**II**).

As illustrated in Figure 1a, tributyltin hydride has the double role of allowing the formation of Bu_3_Sn^•^ as the radical chain carrier and as a hydrogen donor to close the catalytic cycle. The unique features of this catalytic cycle are attributed to the forging of stronger Sn–X and C–H bonds at the expense of the cleavage of the more labile Sn–H and C–X ones. A more quantitative aspect of this reaction can be appreciated comparing the different bond dissociation energies (BDE) associated with the steps mentioned above (see Figure 1a).^14,15^ More recent applications showcase the crucial role of tin intermediates in controlling the outcome of different reactions. Sn–O interactions direct the regioselective addition of the radical in the radical stannylation of the triple bond in propargyloxy derivatives,^16^ whereas tin radicals induced the synthesis of stannylated polyarenes via double radical peri-annulations, increasing the solubility of the products.^17^

The performance of Bu_3_SnH was so competitive^9,18−20^ that more than 20 years ago it was claimed that it was improbable to have “flight from the tyranny of tin” in radical processes,^21,22^ a hard statement that subtly introduces the problem of the substantial toxicity and high biological activity of triorganotin compounds.^23^ The LD_50_ of 0.7 mmol/kg in murine species (see Figure 1b) combined with the long half-lives in aquatic environment represent the biggest concerns for the application of these otherwise extremely versatile species, especially in the absence of viable alternatives.^24^ Indeed, *O*-thiocarbonyl derivatives like xanthates (**I**, obtained from alcohols) were considered an alternative to the alkyl halides, albeit the radical generation required in most cases the use of tin hydrides (Figure 1c).^3,22,25,26^

Efforts in substituting toxic tin derivatives with other hydrides such as (TMS)_3_SiH^27^ or lauroyl peroxides and xanthates met some success, however, only in limited cases.^28−30^ Other initiators to promote tin-free radical chain reactions were organoboranes,^31^ thiols,^32^ P–H-based reagents,^32^ and 1-functionalized cyclohexa-2,5-dienes,^32−34^ but nowadays they are not commonly used in synthetic planning.

The use of metal oxidants (Mn^III^ acetate)^35^ or metal reductants (Ti^III^ catalyst^36^ or Sm^II^ iodide^37^) were sparsely used, but only in the latter case unstabilized alkyl radicals were formed from alkyl iodides.

The introduction of the Barton esters **II** in 1985 represented a step forward in solving the conundrum of the tyranny of tin: the conversion of the strong O–H bond of an acid into the (photo)labile O–N bond of the corresponding thiohydroxamate ester (Figure 1d).^38,39^ Barton esters have the advantage of being slightly colored, allowing the use of visible light irradiation to induce the cleavage of the O–N bond. The last point is significant, demonstrating the formation of alkyl radicals in a very mild way under tin-free conditions with no need of further additives, albeit Barton esters have currently a limited application. On this occasion, photochemistry showed an attractive potential for the development of novel synthetic strategies based on radical chemistry. However, Barton esters remained for several years an isolated niche. In most cases, the photochemical generation of radicals required harmful UV radiation and dedicated equipment.^40^ Since the milestone represented by the development of the chemistry of Barton esters, new photochemical ways were sought toward more efficient ways to generate radicals. The photon appears to be the ideal component for a chemical reaction, assuming the form of a traceless reagent, catalyst, or promoter that leaves no toxic residues in the final mixture.^41−44^ The breakthrough that would allow moving forward from the “tyranny of tin” to a greener “photon democracy” can be associated with the use of solar or visible photons, freely available from the sun that shines throughout the scientific world. The renaissance of the photocatalyzed processes that we have witnessed in the last years represents a significant step toward this direction.^45−58^

The multifaceted use of photoredox catalysis and photocatalyzed hydrogen transfer reactions expanded the range of possible radical precursors and unconventional routes for the generation of several carbon (or heteroatom based) radicals, including the challenging formation of unstabilized alkyl radicals.^59−65^ Consequently, in this review, we aim to present a summary of the novel ways to generate alkyl radicals by photochemical means that, in the last years^66^ have revolutionized the way to carry out radical chemistry. This work will focus exclusively on the reactions promoting the formation of unstabilized alkyl radicals, and not the stabilized ones, e.g., α-oxy, α-amino, benzylic, or allylic.

**Figure 2:**
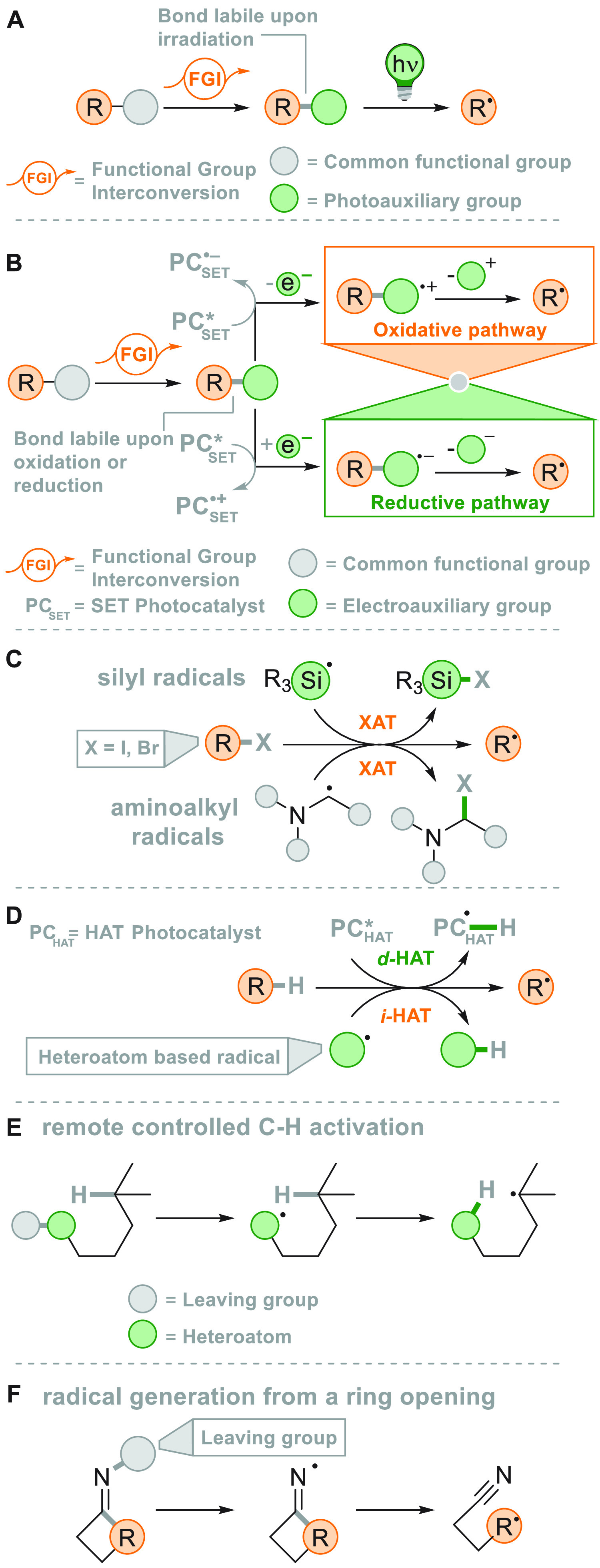
Different approaches for the photogeneration of alkyl radicals (A) by photochemical means through the introduction of a photoauxiliary group (B) via fragmentation of a radical cation (oxidative pathway) or anion (reductive pathway) formed by photoredox catalysis (C) via a halogen atom transfer reaction (XAT) with a photogenerated radical (D) through the photocatalyzed cleavage of a C–H bond via direct (*d*-HAT) or indirect (*i*-HAT) hydrogen atom transfer (E) by the remote-controlled C–H activation via a photogenerated heteroatom based radical (F) by a ring-opening via a photogenerated heteroatom (nitrogen) based radical.

**Figure 3:**
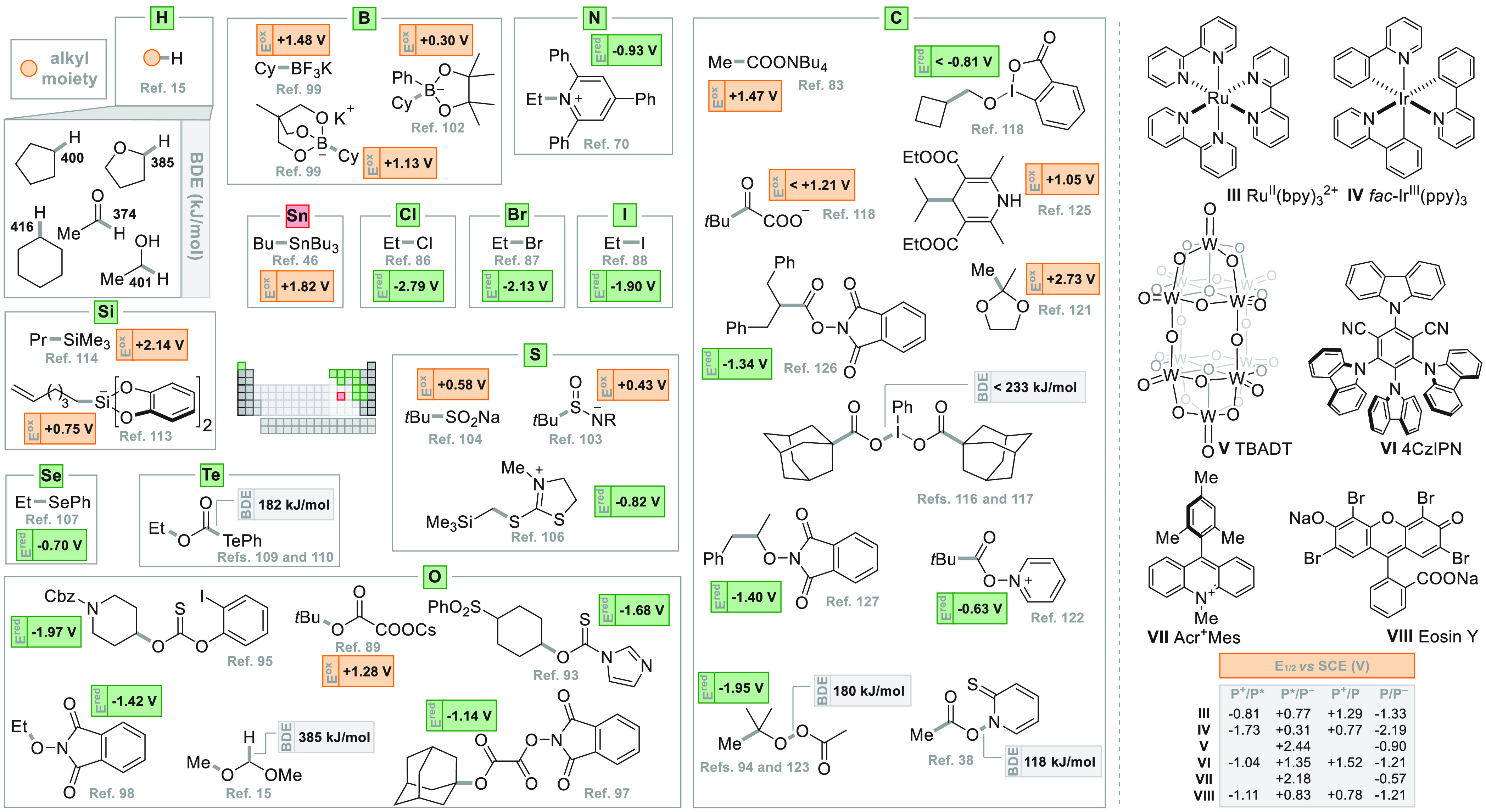
On the left, substrates used to promote the photochemical formation of alkyl radicals divided according to the C–Y bond cleaved. The oxidation potentials (*E*^ox^, in orange) or the reduction potentials (*E*^red^, in green) of the precursors as well as the BDE values of the bond that is broken (highlighted in gray) by direct photocleavage are reported. On the right, a selection of common photoredox catalysts with their main redox features are collected.

Figure 2 collects the main paradigmatic approaches to the photogeneration of alkyl radicals (either photochemically or photocatalyzed). The more classical, although the less employed, path to generate alkyl radicals consists of the introduction of a photoauxiliary group which renders a bond labile to a direct photochemical cleavage (Figure 2A).^67^ The Barton esters are the archetypal moiety belonging to this class.^39^ A conspicuous body of literature have been focusing on the development of suitable alkyl substituents able to facilitate redox reactions making the derivatives more oxidizable or reducible. The strategy that is followed in Figure 2B consists in the conversion of a common functional group (e.g., OH or COOH), which in most cases is tethered to the alkyl group, into a different electroauxiliary group^68^ (Figure 2B). As a result, the interaction of the activated species with an excited photoredox catalyst (PC_SET_) able to induce a single electron transfer (SET) process generates the corresponding radical ions, either by an oxidative pathway or a reductive pathway. The desired alkyl radical is then formed by fragmentation of these radical ion intermediates. The oxidative pathway is efficient when the radical precursor is negatively charged (see further Figure 3) as in the case of alkyl carboxylates and alkyl sulfinates causing the CO_2_ or SO_2_ loss, respectively, despite the fact that the exothermicity of the process is verified only in the C–C cleavage rather than the C–S cleavage.^69^ On the contrary, positively charged Katritzky salts were ideal candidates for the releasing of radicals via the reductive pathway (Figure 3).^70^

A viable alternative is the photogeneration (often from a photoredox process) of a reactive radical on a heteroatom like a silyl radical, which can exploit a halogen atom transfer reaction to afford an alkyl radical through the smooth Si-X bond formation (XAT, Figure 2C).^71,72^ This strategy provides an elegant way to overcome the Giese conditions in the tin mediated activation of alkyl halides. Recently, an α-amino radical was used in the same strategy, promoting the formation of alkyl radicals via C–X bond cleavage.^73^

A more challenging approach requires the photocatalyzed selective cleavage of a strong alkyl-H bond, via a direct hydrogen atom transfer reaction (*d*-HAT, Figure 2D) operated by an excited photocatalyst (PC_HAT_).^72,74−76^ The indirect version of the latter path exploits the photogeneration of a stable heteroatom based radical (*i*-HAT, Figure 2D) that will become the competent intermediate in the abstraction of the H atom from the alkyl moiety.^76^ An indirect HAT (*i*-HAT) may also take place by intramolecular hydrogen transfer thus releasing an alkyl radical (Figure 2E).^76−81^ As an alternative, the photochemical radical generation may induce a ring-opening in strained structures like cyclobutanes, to form a substituted alkyl radical (Figure 2F).^82^

Figure 3 showcases a collection of the main alkyl radical precursors devised for the generation under photochemical conditions of unstabilized alkyl radicals. In this figure, the radical precursors were collected depending on the C(sp^3^)-Y bond cleaved during the radical release. As apparent, the photochemically triggered cleavage of several C-heteroatom bonds like C–X,^71,83−88^ C–O,^89−98^ C–B,^99−102^ C–S,^103−106^ or C–N^70^ (Figure 3) affords carbon-centered radicals. The alkyl radical generation is granted by the very versatile photochemical tool. This feature includes particular cases such as C–Se (in alkyl selenides),^107,108^ C–Te [in (aryltelluro)formates,^109,110^ for a previous thermal generation of alkyl radical from diorganyl tellurides, see ref (111)], and C–Si (in tetra alkyl silanes and bis-chatecolates)^112−114^ to be added to C–Sn (in alkyl stannanes).^112,115^ Interestingly, even the more resilient C–H^15,76^ or C–C^38,83,94,116−130^ bonds may be cleaved for alkyl radical generation, opening up new exciting possibilities for the synthetic (photo)chemist (Figure 3).

For the clarity of the reader, each radical precursor is accompanied by its oxidation potential (*E*_OX_, in orange) or its reduction potential (*E*_RED_, in green) to guide the feasibility on the generation of the radical via the oxidative or reductive pathway (type B, Figure 2B), respectively. Since the redox potentials may vary with the nature of the alkyl group, the values reported are referred to known structures. In alternative, the BDE values of the bond that is broken by direct photocleavage (type A, Figure 2A) or by photocatalyzed hydrogen abstraction (type D, Figure 2D) are reported. Figure 3 (right part) likewise collects the redox properties of commonly used photoredox catalysts including metal-free photoorganocatalysts (POC) to be used in the oxidative/reductive pathways.^131−137^

The reactions collected and commented on in this review are primarily divided according to the type of the bond formed, namely the forging of C–C or C–heteroatom bonds, along with the construction of rings of different sizes. When possible, in each section, we will further categorize the reactions depending on the mechanism of the radical generation, ascribing them to the six types (A–F) described in Figure 2.

## 2 Formation of a C(sp^3^)-C Bond

Photochemically generated alkyl radicals have been employed to forge C(sp^3^)-C(sp^*n*^) bonds (*n* = 1–3) in an intermolecular fashion following different strategies. In most cases, a conjugate addition onto a Michael acceptor or a Minisci-like reaction occurred, albeit alkenylations, acylations, or oxyalkylations are likewise used.

### 2.1 Formation of a C(sp^3^)-C(sp^3^) Bond

#### 2.1.1 Addition to C–C Double Bonds: Hydroalkylations

Many reactions belonging to this class involve the nucleophilic alkyl radical addition onto an electrophilic Michael acceptor, resulting in a formal hydroalkylation of the double bond *viz*. the incorporation of an alkyl group (in position β with respect to the EWG group in the starting unsaturated compound) and a hydrogen atom (in position α). This reaction is usually one of the first that many authors would test during the discovery process of a new radical precursor, as testified by the plethora of reagents that are used in this transformation. Photoredox catalysis is by far the preferred approach here, especially by using the oxidation of a negatively charged precursor (oxidative pathway in Figure 2B).

**Scheme 1:**
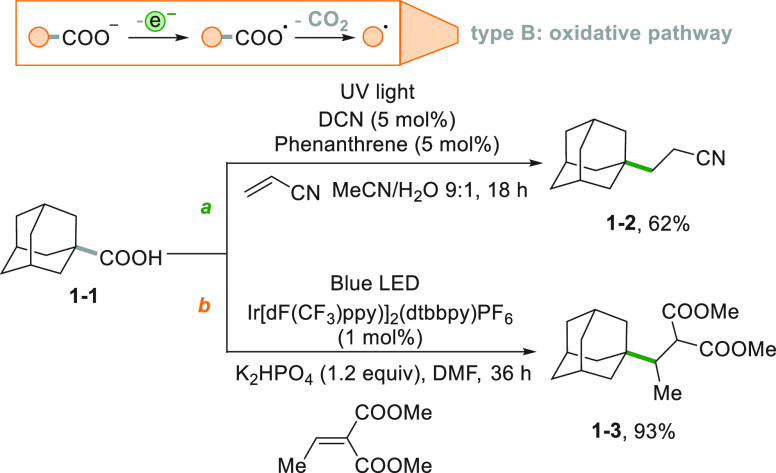
Different Strategies for the Decarboxylative Adamantylation of Electron-Poor Alkenes

A typical example is the oxidation of carboxylates^138,139^ that releases an alkyl radical via CO_2_ loss from the carboxyl radical intermediate (Scheme 1). Adamantylation of both acrylonitrile (Scheme 1a)^140^ and dimethyl 2-ethylidenemalonate starting from adamantane carboxylic acid **1**-**1** (Scheme 1b)^141^ were carried out following this strategy. In the former case, the authors employed 1,4-dicyanonaphthalene (DCN) as the POC under UV light irradiation, while visible light and an Ir^III^ complex in the latter case. The approach used in Scheme 1b was also useful for the three steps preparation of the medicinal agent (±)-pregabalin.^141^ Also, the Fukuzumi catalyst (9-mesitylene-10-methylacridinium perchlorate, [Acr^+^Mes]ClO_4_) can promote this Giese-type reaction,^142^ allowing the alkylation of α-aryl ethenylphosphonates for the synthesis of fosmidomycin analogues.^143^

**Scheme 2:**
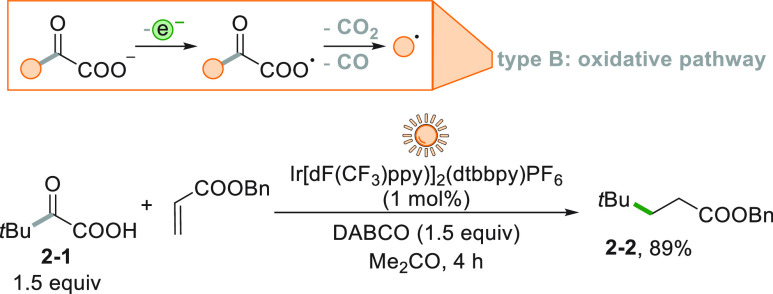
Decarboxylative-Decarbonylation of an α-Keto Acid

A variation of this procedure is the decarboxylative-decarbonylative process occurring on an α-keto acid **2**-**1** under sunlight-driven photoredox catalyzed reaction conditions (Scheme 2).^125^

**Scheme 3:**
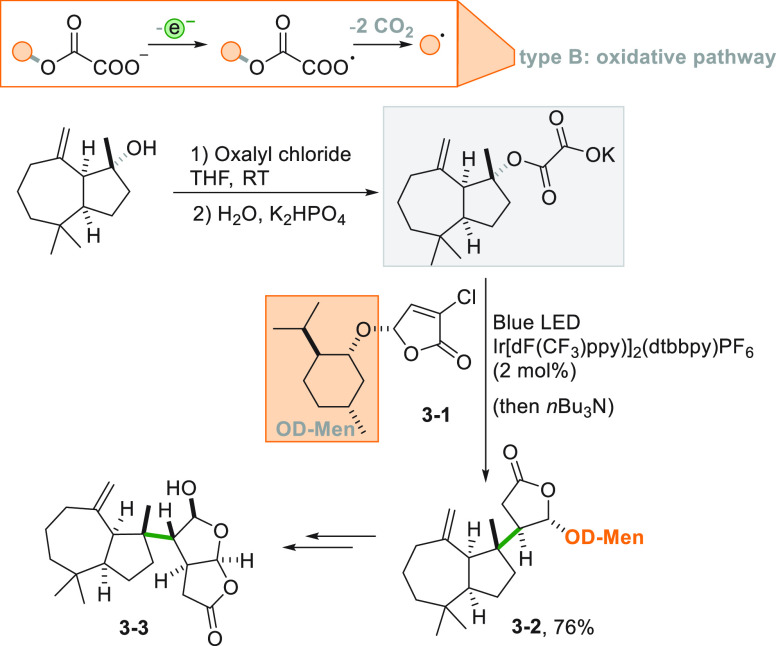
Enantioselective Preparation of Cheloviolene A

Oxalates are another class of electron-donors having two carboxylate moieties that can be lost upon photocatalyzed oxidation. These species may be introduced *in situ* by reaction of the alcohol with oxalyl chloride. The process induced the cleavage of a C–O bond, and the resulting radical could be trapped by butenolide **3**–**1** to form the menthyl derivative **3**–**2** used for the enantioselective preparation of cheloviolene A (**3**–**3**, Scheme 3).^144^ An Ir^III^-based photocatalyst efficiently promoted the reaction also in this case, allowing the synthesis of quaternary centers^89^ and the total synthesis of trans-clerodane diterpenoids.^145^

**Scheme 4:**
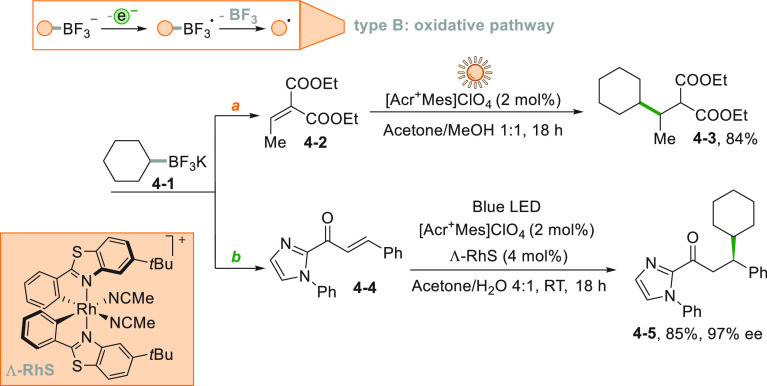
Visible and Solar Light Photocatalyzed Functionalization of Michael Acceptors with Alkyl Trifluoroborate Salts

Alkyl trifluoroborates stand out as another important class of easily oxidizable moieties.^146^ The photocatalyzed oxidation of these salts (e.g., **4**–**1**) causes the smooth release of BF_3_ and the formation of the reactive alkyl radical. Such a reaction was employed to functionalize Michael acceptors under sunlight irradiation (Scheme 4a) exploiting Acr^+^Mes as the POC.^147^ Complexation of **4**–**4** by a chiral rhodium complex (Λ-RhS, Scheme 4b) delivered **4**–**5** in good yields with 97% ee.^148^

**Scheme 5:**
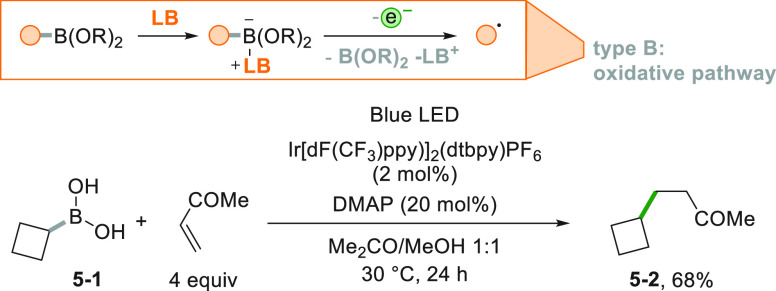
Activation of Boronic Acids with a Lewis Base

This synthetic strategy can be extended to neutral boronic acids or esters, upon *in situ* activation by a Lewis base (LB). The so formed negatively charged species is consequently more prone to oxidation, which eventually will provide the formation of the alkyl radicals. A typical example is illustrated in Scheme 5 where the boronic acid **5**–**1** was activated by 4-dimethylaminopyridine (DMAP) and then oxidized by an Ir^III^ complex. The resulting cyclobutyl radical was trapped by methyl vinyl ketone to access the substituted ketone **5**–**2** in a good yield.^100^ This reaction was later scaled up under flow conditions by using the *Photosyn* reactor. In such a way, the authors could synthesize gram amounts per hour of the analogues of some drugs belonging to the GABA family.^149^

**Scheme 6:**
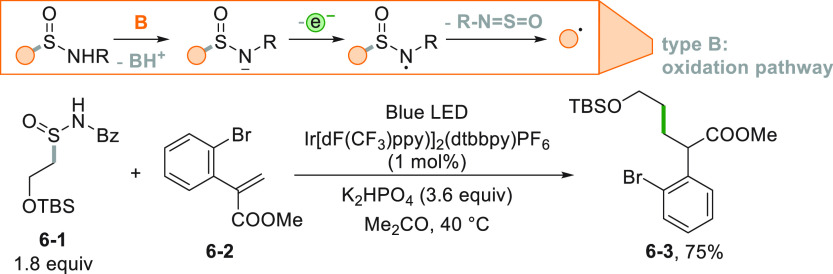
Desulfurative Strategy for the Conjugate Addition of Alkyl Radicals onto Michael Acceptors

Following the examples of the carboxylate derivatives, the electron-donating species may be generated *in situ* by deprotonation, as in the case of sulfonamides, employed in the desulfurative conjugate addition of alkyl radicals onto Michael acceptors (Scheme 6). Again, the process is based on a photocatalyzed oxidation pathway. The starting sulfonamide (**6**–**1**) was first deprotonated by a mild base (K_2_HPO_4_), and the resulting anion was easily oxidized to a *N*-centered radical. Loss of *N*-sulfinylbenzamide generates the desired radical that gave the adduct **6**–**3** upon reaction with **6**–**2** in 75% yield.^103^

**Scheme 7:**
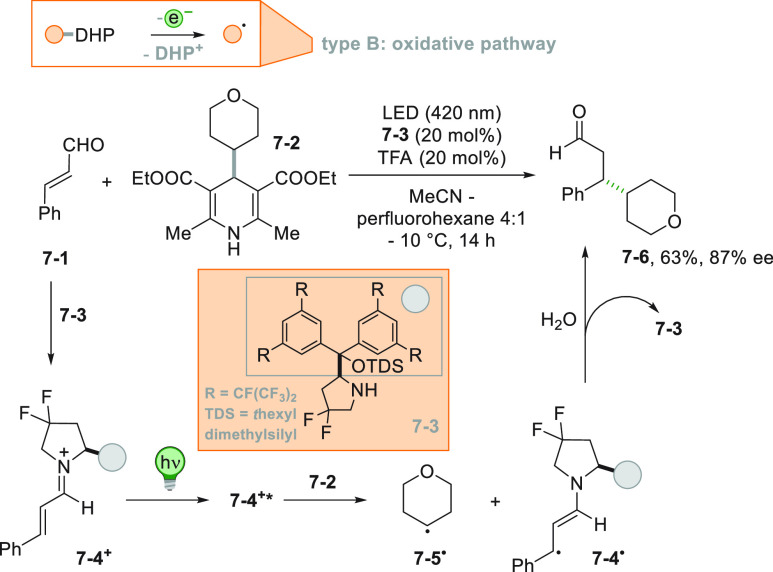
Alkyl-DHPs as Radical Precursors in Combination with Iminium Catalysis

In some instances, the radical precursor is a neutral compound. This situation is possible only when the derivative contains a highly oxidizable or reducible moiety. 4-Alkyl-1,4-dihydropyridines (alkyl-DHPs) under PC-free conditions act as radical precursors when combined with photoexcited iminium ion catalysis (Scheme 7). Here, enal **7**–**1** formed a chiral iminium ion **7**–**4**^**+**^ by reaction with amine **7**–**3**. Cation **7**–**4**^**+**^ upon visible light excitation oxidized the alkyl-DHP **7**–**2** that in turn released the radical **7**–**5**^**•**^ upon fragmentation, along with radical **7**–**4**^**•**^. Radical recombination followed by hydrolysis gave the desired alkylated dihydrocinnamaldehyde **7**–**6** in a satisfactory yield with a good enantiomeric excess (Scheme 7).^150^ A similar Giese reaction was later proposed, where the alkyl-DHP was excited and a SET reaction with Ni(bpy)_3_^2+^, acting as an electron mediator, took place. The alkyl radical derived from the radical cation of alkyl-DHP readily attacked a series of Michael acceptors.^151^

**Scheme 8:**
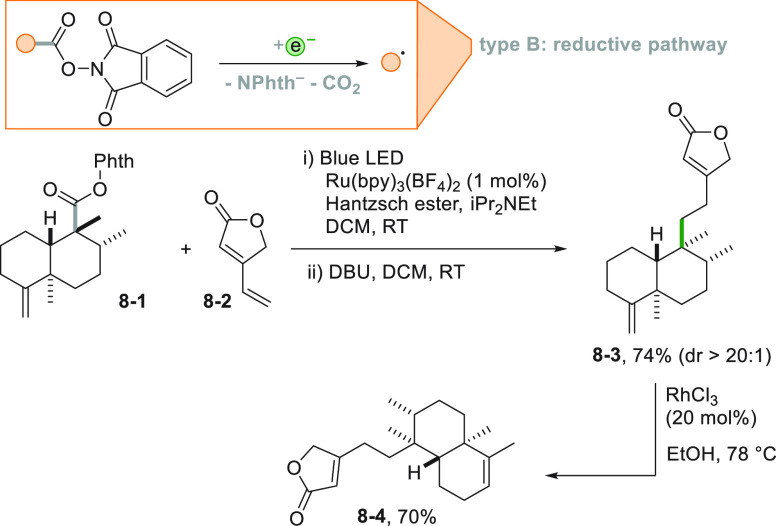
Synthesis of (−)-Solidagolactone via *N*-(Acyloxy)phthalimides

Looking at the other edge of the redox spectrum, easily reducible compounds were devised as radical precursors via a photocatalyzed process. As an example, the incorporation of a *N*-phthalimidoyl moiety in an organic compound helps its photocatalyzed reduction, ultimately leading to the release of the alkyl radical. A typical case is represented by *N*-(acyloxy)phthalimides.^126^ A stereoselective variant of this reaction was applied to the synthesis of (−)-solidagolactone (**8**–**4**, Scheme 8). Thus, the photocatalyzed reduction of phthalimide **8**–**1** by a Ru^II^ complex released a tertiary carbon radical. Attack to the terminal carbon of the unsaturated core present in β-vinylbutenolide **8**–**2** yields **8**–**3** with a very high diastereomeric excess. Further elaboration of compound **8**–**3** gave **8**–**4** in a single step.^152^ This reaction emerges as a very interesting tool to construct quaternary carbons^153^ and to synthesize biologically active derivatives, e.g., (−)-aplyviolene.^154^

**Scheme 9:**
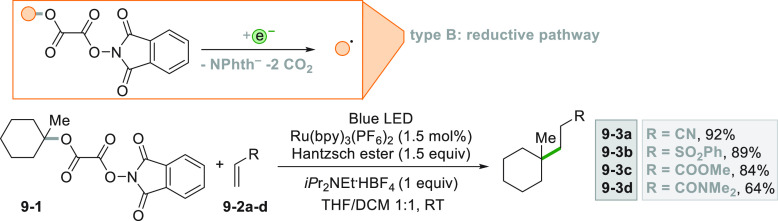
Generation of Alkyl Radicals from *N*-Phthalimidoyl Oxalates

Interesting results were also obtained using *N*-phthalimidoyl oxalates such as **9**–**1** in place of the *N*-(acyloxy)phthalimides for the generation of alkyl radicals starting from tertiary alcohols (Scheme 9).^92,97^ The similarity of this reaction to the one presented in Scheme 8 is striking, despite a less atom economical radical generation.

**Scheme 10:**
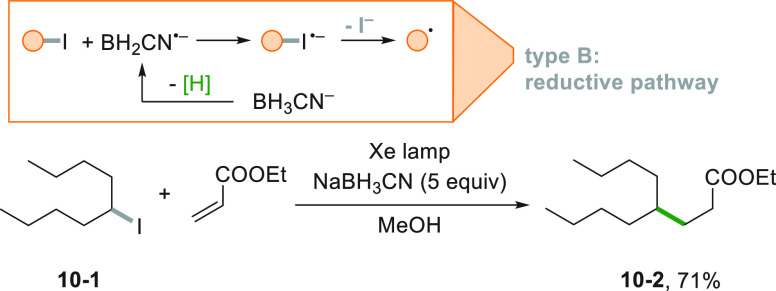
Giese-Type Reaction of Iodides in the Presence of BH_3_CN^–^

Reduction of an organic compound may be carried out even on organic iodides by using cyanoborohydride anion as the reducing agent. The reaction is chemoselective, since no alkyl bromides or chlorides could be activated following this way. Giese adducts were formed by irradiation with a Xe lamp of the reaction mixture in good yields as illustrated by the formation of **10**–**2** from **10**–**1** in Scheme 10.^155^ This is another interesting example to circumvent the use of tin hydrides in the activation of alkyl halides.

Alkyl chlorides can be activated using Ir(dtbby)(ppy)_2_PF_6_ in the presence of micelles. The micellar environment stabilizes the photogenerated [Ir(dtbby)^•–^(ppy)_2_] species (−1.51 V vs SCE), unable to directly reduce the alkyl chlorides (ca. −2.8 V vs SCE). A second excitation of this long-lived intermediate allows the electron transfer to the halide, which could react with different electron-poor olefins, forging a novel C–C bond. The micellar system allowed intramolecular cyclizations to form five-membered cycles.^156^

**Scheme 11:**
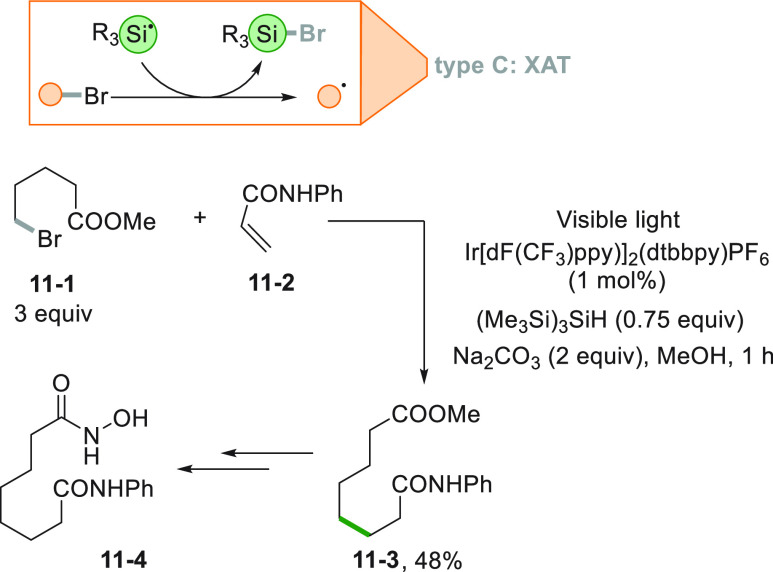
Synthesis of Vorinostat *via* XAT Strategy

Reduction of the alkyl halide **11**–**1** could be avoided applying a halogen transfer reaction. In fact, due to the strong BDE of the Si-halogen bond, an alkyl radical is formed thanks to the action of a purposely generated silyl radical (from (Me_3_Si)_3_SiH, TTMSS) by a photoredox catalytic step. Radical addition onto an unsaturated amide (**11**–**2**) gave the 1,8-difunctionalized derivative **11**–**3**, a key compound in the preparation of Vorinostat **11**–**4**, a histone deacetylase (HDAC) inhibitor active against HIV and cancer (Scheme 11).^157^ This is the typical case where the radical is formed by the cleavage of an Alk-Br bond without the assistance of tin derivatives. It is interesting that the reaction requires a substoichiometric amount of silane to proceed. Indeed, with higher loadings the product yield decreases, possibly due to the presence of competing nonproductive pathways. A chain reaction mechanism could be envisaged; however, the quantum yield for this reaction (Φ = 0.45) does not fully clarify the mechanistic details of the transformation.

**Scheme 12:**
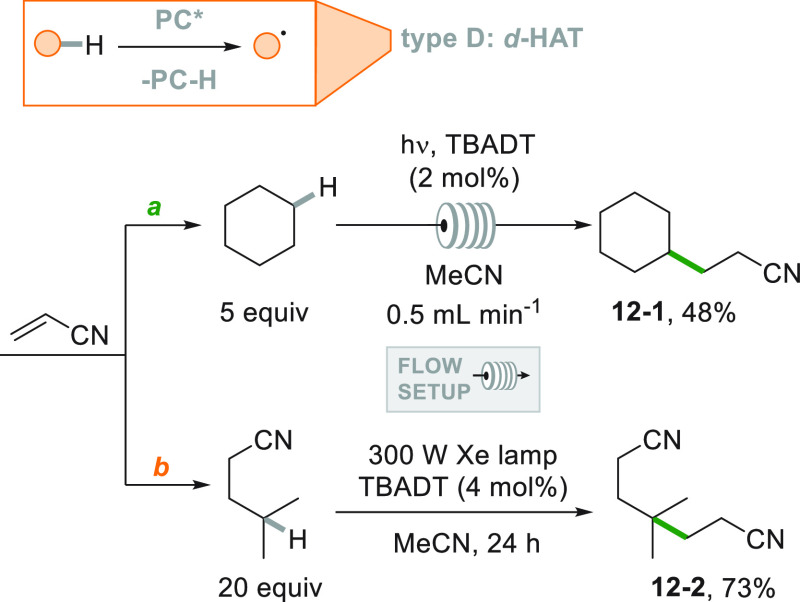
TBADT-Photocatalyzed Hydroalkylation of Acrylonitrile

In many instances the formation of the alkyl radical arose from a direct or indirect photocatalyzed C–H homolytic cleavage. The excited state of the decatungstate anion in its tetrabutylammonium salt form (TBADT) promoted in several cases the direct chemoselective cleavage of a C–H bond.^75,158^Scheme 12 depicts two examples involving the hydroalkylation of acrylonitrile. Unsubstituted cycloalkanes were suitable hydrogen donors under flow conditions (yielding **12**–**1**Scheme 12a).^159^ Moreover, the chemoselective cleavage of the methine hydrogen in isovaleronitrile allowed the preparation of dinitrile **12**–**2** in 73% yield (Scheme 12b).^160^

**Scheme 13:**
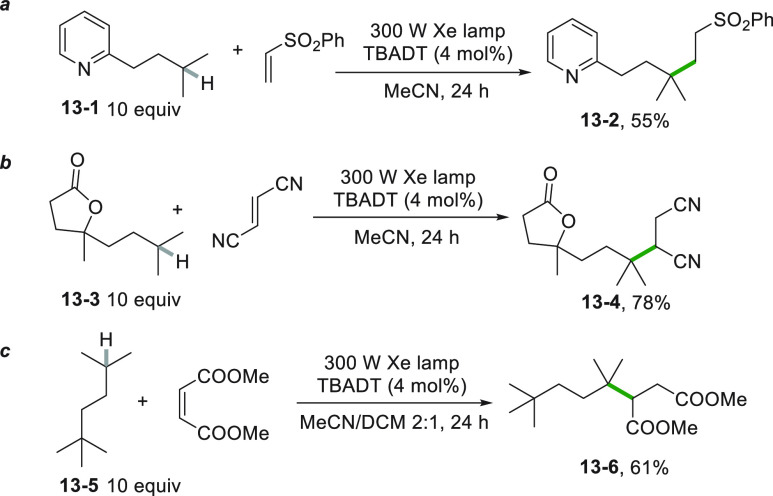
TBADT-Driven Functionalization of Tertiary Carbons

Similarly, the presence of a tertiary hydrogen was the driving force of the chemoselective TBADT-photocatalyzed C–H cleavage in several derivatives, as depicted in Scheme 13. As an example, alkylpyridine **13**–**1** was selectively functionalized and gave derivative **13**–**2** as the exclusive product in the reaction with a vinyl sulfone (Scheme 13a).^161^ Interestingly, the labile benzylic hydrogens present in **13**–**1** remained untouched under these reaction conditions. Noteworthy, steric and polar effects cooperatively operated in the derivatization of lactone **13**–**3**. As a result only the methine hydrogen of the isopropyl group was selectively abstracted and afforded **13**–**4** in very high yields by reaction with fumaronitrile (Scheme 13b), albeit the seven different types of hydrogens present in **13**–**3**.^162^ The C–H cleavage may also take place in branched alkanes as witnessed by the derivatization of **13**–**5** to form the succinate derivative **13**–**6** (Scheme 13c).^163^

**Scheme 14:**
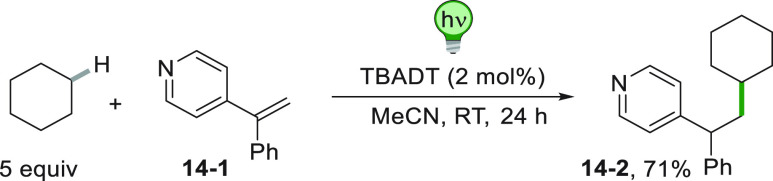
Functionalization of a Vinylpyridine with a Cycloalkane

In rare instances, the hydroalkylation reaction may be applied to olefins different to the usual Michael acceptors. Thus, substituted vinylpyridines were functionalized by TBADT-photocatalyzed addition of cycloalkanes. Scheme 14 showed the smooth synthesis of **14**–**2** starting from **14**–**1** simply by irradiation of the reaction mixture containing a slight excess of cyclohexane in the presence of a catalytic amount of the decatungstate salt.^164^

**Scheme 15:**
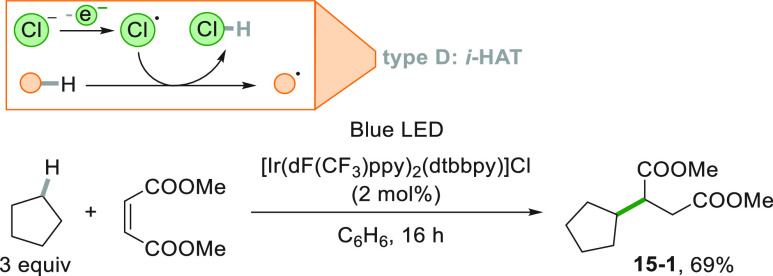
Indirect HAT Mediated by a Cl^•^ Radical

Recently, alternative PCs have been developed for the direct photocatalyzed activation of C–H bonds in cycloalkanes, namely uranyl cation^165^ and Eosin Y,^166^ both having the advantage of absorbing in the visible light region. The alkyl radical formation may be induced by a photogenerated stable radical which acts as a radical mediator. An Ir^III^ based photoredox catalyst oxidized the chloride anion (being the counterion of the Ir complex) to the corresponding chlorine atom, which abstracted a hydrogen atom from cyclopentane, thus forming adduct **15**–**1** in 69% yield upon addition onto a maleate ester (Scheme 15).^167^

**Scheme 16:**
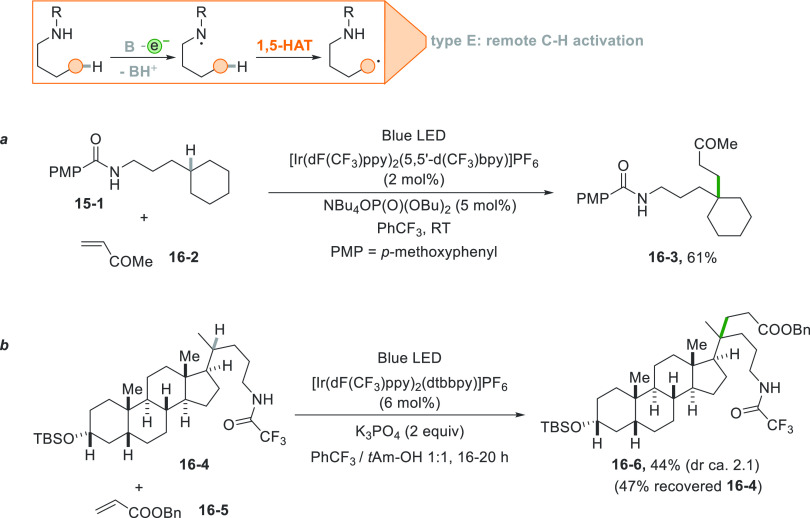
Intramolecular 1,5-HAT Forming Tertiary Alkyl Radicals

Another intriguing way to induce the cleavage of unactivated C(sp^3^)-H bonds is by a photocatalyzed intramolecular hydrogen abstraction. Usually a photoredox or a proton-coupled electron transfer (PCET)^47^ step induced the formation of a heteroatom centered radical that abstracts a tertiary C–H bond intramolecularly in a selective fashion, following a 1,5-HAT process mimicking the Hoffmann-Löffler-Freytag reaction (Scheme 16).^168−170^

When the reaction was applied to compound **16**–**1**, an oxidative PCET generated a neutral amidine radical that promotes the 1,5-hydrogen atom abstraction forming a tertiary radical which is able to functionalize olefin **16**–**2** in a complete regioselective fashion affording **16**–**3** (Scheme 16a).^171^ The reaction was also applied to medicinally relevant molecules such as the steroid-derived trifluoroacetamide **16**–**4** (Scheme 16b). Despite the fact that this compound has several labile C–H bonds including tertiary C–H bonds and C–H bonds adjacent to heteroatoms, the intramolecular hydrogen abstraction followed by conjugate addition onto **16**–**5** gave **16**–**6** as the sole product.^172^

The remote activation of the C–H bond in the δ-position following this approach is a general reaction as demonstrated in related systems applied to amides protected with a carbamate group^173^ or in simple benzamide derivatives.^174^ In the latter case, the reaction was carried out in the presence of a chiral Rh-based Lewis acid catalyst that allowed the asymmetric alkylation of α,β-unsaturated 2-acyl imidazoles.^174^

**Scheme 17:**
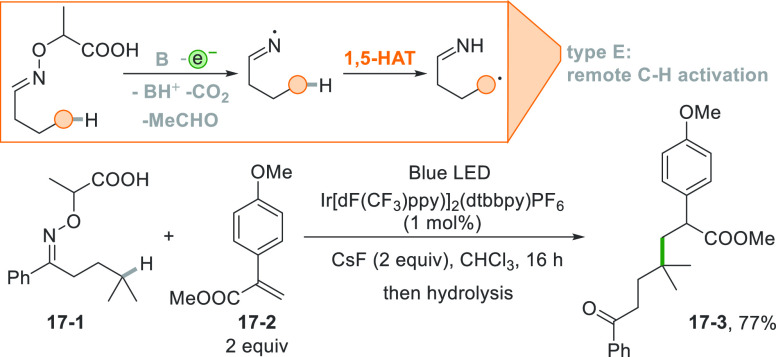
1,5-HAT Promoted by an Iminyl Radical

The abstracting species could be likewise a photogenerated iminyl radical as illustrated in Scheme 17. Here a carbonyl group is converted in an oxime derivative (e.g., **17**–**1**) by reaction with an α-aminoxy acid. Photocatalyzed oxidation followed by fragmentation of the resulting carbonyloxy radical gave an iminyl radical prone to a 1,5-HAT to afford a tertiary radical that upon addition to acrylate **17**–**2** gave compound **17**–**3** in 77% yield.^175^

#### 2.1.2 Heteroalkylation of C–C Double Bonds

**Scheme 18:**
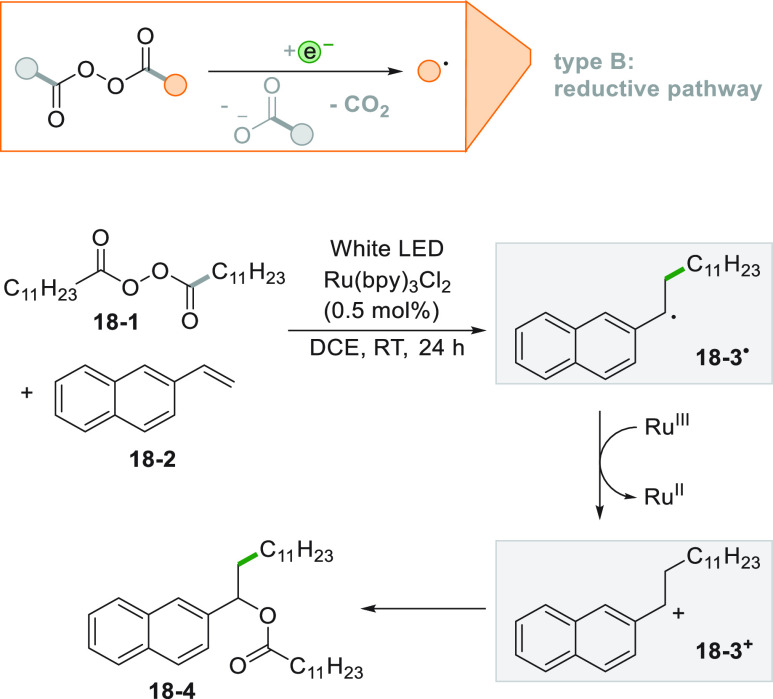
Oxyalkylation via Alkyl Diacyl Peroxides

An interesting variation of the functionalization of a double bond is the formation of a C–C bond (upon an alkylation step) followed by the formation of another C–Y bond (Y ≠ H) on the adjacent carbon. As an example, alkyl diacyl peroxides were reduced photocatalytically and the fragmentation released an alkyl radical and a carboxylate anion both incorporated in the structure of the product. Thus, 2-vinylnaphthalene **18**–**2** was converted into compound **18**–**4** in a very good yield upon reaction with lauroyl peroxide **18**–**1** upon an oxidative quenching process by consecutive C–C and C–O formation (Scheme 18).^176^ The reaction was made possible by the oxidation of the resulting radical adduct **18**–**3**^**•**^ (by Ru^III^, the oxidized form of the PC) that generated the cation **18**–**3**^**+**^ that was easily trapped by the carboxylate anion previously released.

**Scheme 19:**
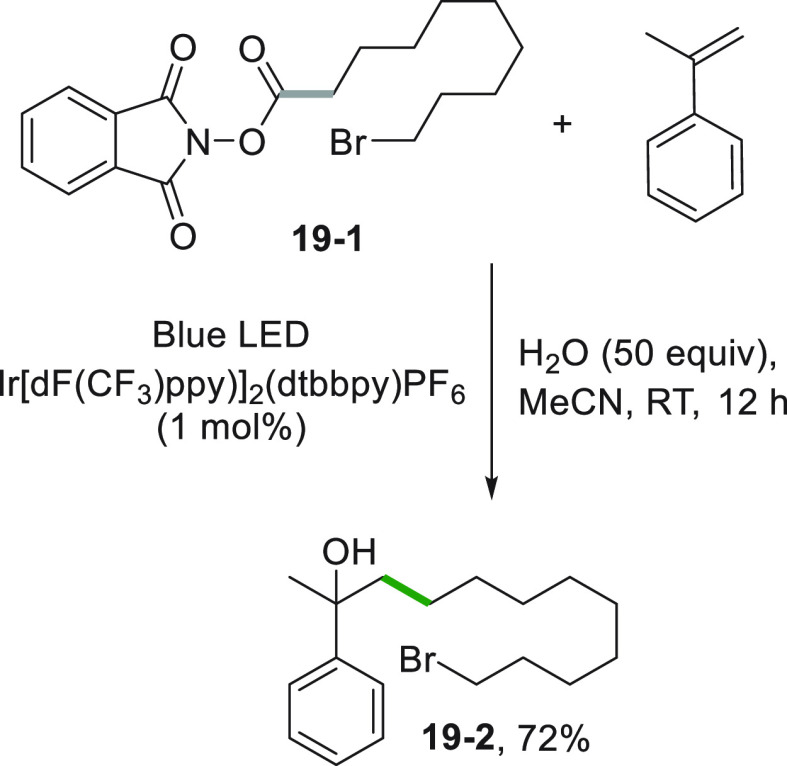
Multicomponent Oxyalkylation of Styrenes

*N*-(acyloxy)phthalimide **19**–**1** as radical precursor found use in a similar multicomponent oxyalkylation of styrenes. The addition of the alkyl radical onto the vinylarene followed by the incorporation of water present in the reaction mixture afforded derivative **19**–**2** in 72% yield (Scheme 19).^177^ Noteworthy, the labile C–Br bond in **19**–**1** remained untouched in the process.

The use of water as the oxygen source was likewise used in the difunctionalization of aryl alkenes where the carbon-centered radical was formed by an intramolecular 1,5-HAT of a photogenerated iminyl radical.^178^

**Scheme 20:**
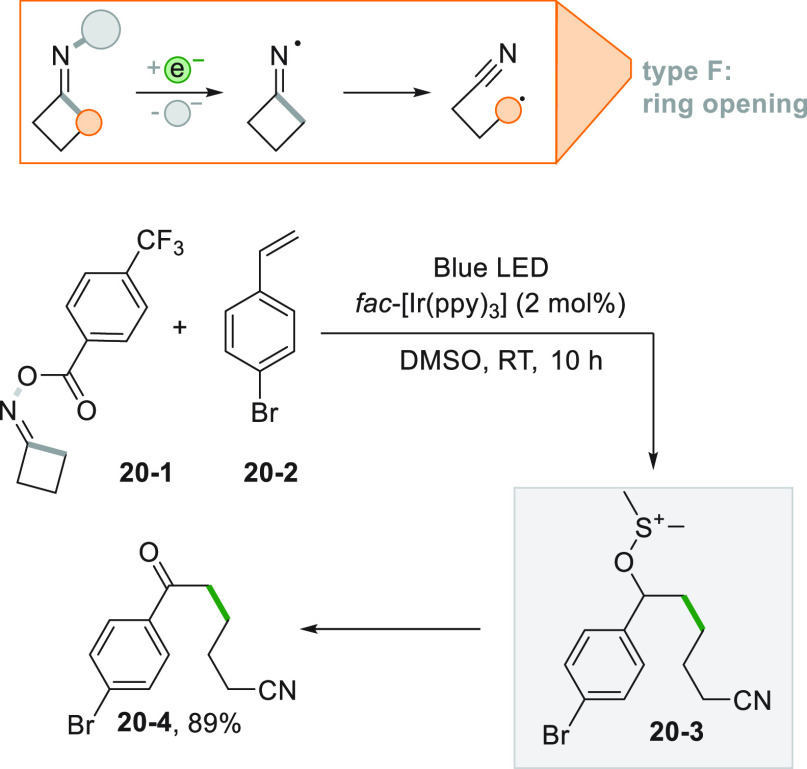
Photocatalyzed Oxyalkylation of Styrenes Based on the Kornblum Oxidation

Performing the reaction in DMSO, allows for the use of the solvent as an oxygen donor adopting the Kornblum oxidation. The intermediate benzyl radical formed after the alkylation step reacts with the solvent and eventually forming a carbonyl group in place of a simple C–O bond. An elegant example is shown in (Scheme 20) for the synthesis of ketonitrile **20**–**4**.^179^ A cycloketone oxime ester (**20**–**1**) was photocatalytically reduced, inducing a ring opening on the resulting iminyl radical. The resulting cyano-substituted alkyl radical reacted with styrene **20**–**2**, and the addition with DMSO formed the intermediate **20**–**3**, that, upon Me_2_S loss, afforded the product.

**Scheme 21:**
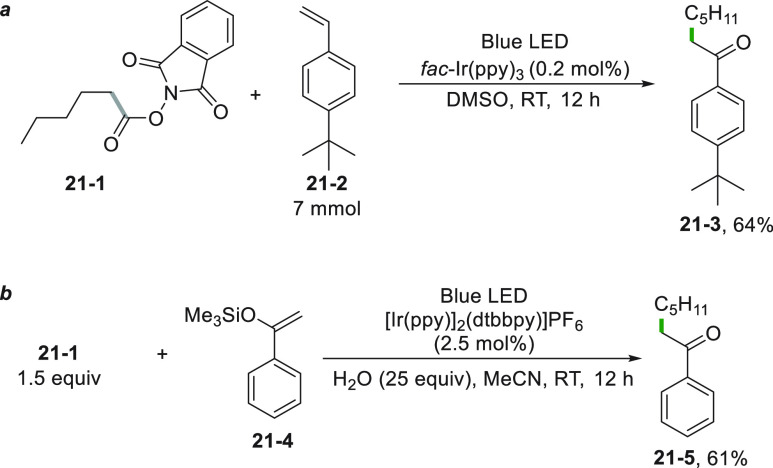
Oxyalkylation by Using *N-*(Acyloxy)phthalimide Derivatives as Radicals Source

A related oxyalkylation of styrenes made again the use of the Kornblum oxidation as the last step in the synthesis of substituted acetophenones. Indeed, *N*-hydroxyphthalimides (e.g., **21**–**1**) were employed as the radical source, and an Ir^III^ complex was used as the PC, obtaining good yields even on a 7 mmol scale (64% of **21**–**3**, Scheme 21a).^180,181^ Ester **21**–**1** was also adopted for the preparation of the aryl alkyl ketone **21**–**5** in 61% yield (Scheme 21b). In this case, however, the decarboxylative alkylation was applied to silyl enol ethers having the carbonyl oxygen already incorporated in the initial structure such as **21**–**4**.^182^ The same process described in Scheme 21b can be carried out under uncatalyzed conditions under blue LED irradiation in the presence of an excess of NaI (150 mol %) and PPh_3_ (20 mol %). The reaction was based on the photoactivation of a complex formed by *N*-(acyloxy)phthalimide with NaI and PPh_3_ through Coulombic and cation-π interactions. In this case, the excitation caused the reduction of the phthalimide by a SET reaction within the complex.^183^

**Scheme 22:**
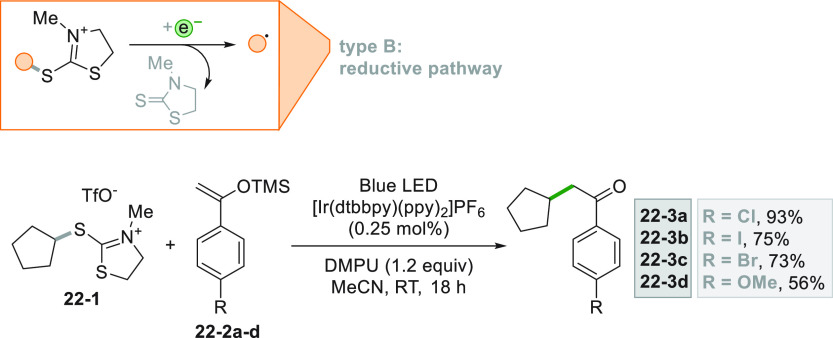
Synthesis of Alkylated Ketones from Mercaptothiazolinium Salts

Alkylated ketones **22–3a–d** were likewise obtained by the Ir^III^-photocatalyzed reaction between a 2-mercaptothiazolinium salt (**22**–**1**, as alkyl radical precursor) and silyl enol ethers **22–2a–d** (Scheme 22).^106^

**Scheme 23:**
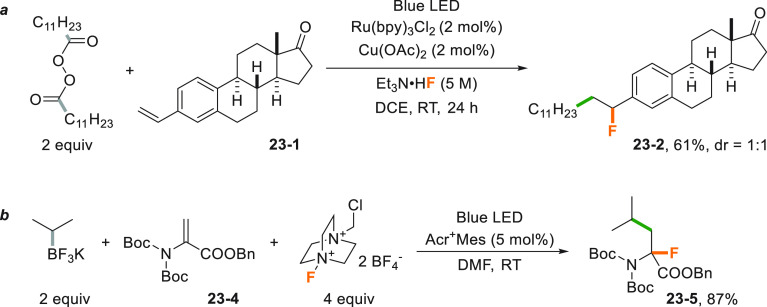
Carbofluorination of (a) Styrenes and (b) Dehidroalanine Derivatives

Lauryl peroxide (LPO, see Scheme 18) was adopted for the Ru-catalyzed three-component carbofluorination of styrenes as illustrated in Scheme 23a. The vinylic double bond of compound **23**–**1** derived from estrone was functionalized twice by using triethylamine trihydrofluoride Et_3_N·HF as the fluoride anion source to deliver the desired alkyl-fluorinated olefin **23**–**2** in 61% yield. The reduction of LPO is mediated by the presence of a copper salt in the role of a cocatalyst in a dual catalytic process.^184^ The carbofluorination was later applied to dehydroalanine derivative **23**–**4** by using alkyltrifluoroborates and an excess of Selectfluor as an electrophilic fluorine source (Scheme 23b). The use of a visible light POC ([Acr^+^Mes]ClO_4_) allowed for the synthesis of a wide range of unnatural α-fluoro-α-amino acids including F-Leu (**23**–**5**).^185^

**Scheme 24:**
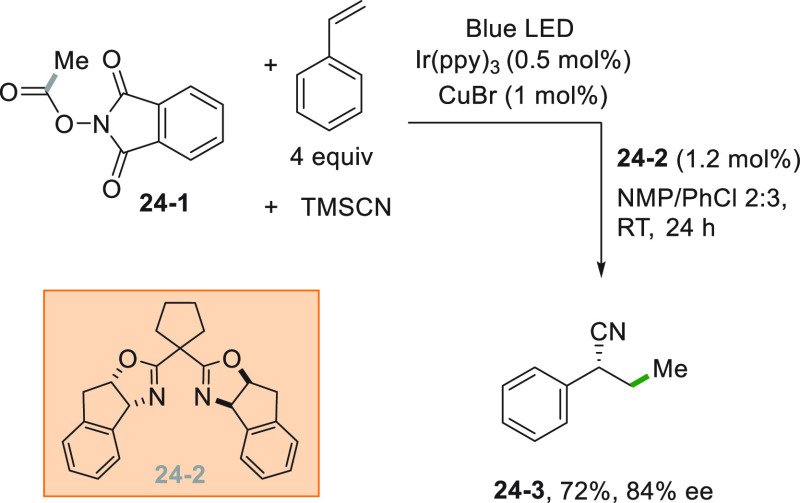
Enantioselective Cyanoalkylation of Styrenes

In rare instances, two C–C bonds could be formed in the adjacent position of the double bond as in cyanoalkylations. The enantioselectivity of the reaction was controlled exploiting the capability of a copper catalyst to form complexes with chiral Box ligands. Thus, a methyl radical was obtained by Ir^III^-photocatalyzed reduction of phthalimide **24**–**1** that readily attacked styrene (Scheme 24). Meanwhile, the Cu^I^ salt incorporated Box **24**–**2** as the ligand, and the resulting complex reacted with the adduct radical in the presence of TMSCN. As a result, cyanoalkylated **24**–**3** was obtained in a good yield and in good ee.^186^

A particular case of cyanoalkylation was later reported in the photocatalyzed reaction between cyclopropanols and cyanohydrins having a pendant C=C bond. Oxidative ring opening of the three-membered ring followed by addition onto the double bond and cyano migration gave a series of multiply functionalized 1,8-diketones incorporating the cyano group.^187^

#### 2.1.3 Allylation

**Scheme 25:**
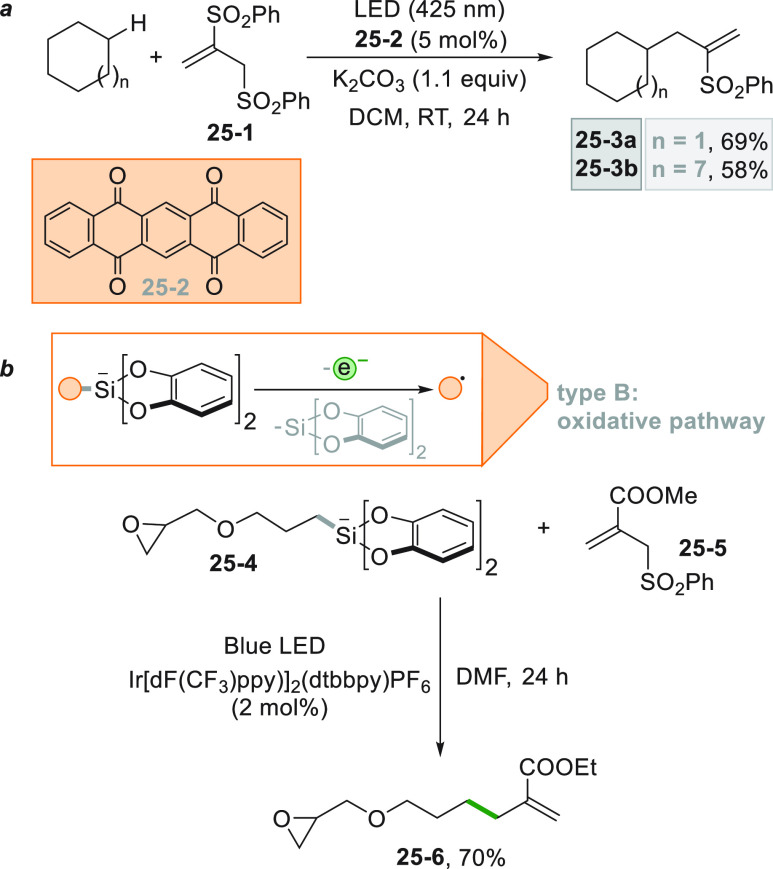
Allylation through Alkyl Radicals Generated (a) *via* HAT and (b) from Si bis-Catecholates

Allylation reactions can be easily performed by reaction of an alkyl radical with substituted allyl sulfones (mainly with 1,2-bis(phenylsulfonyl)-2-propene **25**–**1**, Scheme 25a). The alkyl radical was generated under visible light irradiation by hydrogen abstraction from cycloalkanes by an aromatic ketone, e.g., 5,7,12,14-pentacenetetrone **25**–**2**. Addition of a cycloalkyl radical onto **25**–**1** followed by sulfonyl radical elimination gave access to vinyl sulfones **25–3a–b** in good yields (Scheme 25a).^188^

Other related reactions were designed to forge C(sp^3^)–allyl bonds following this simple scheme. The alkyl radical was formed by photocatalytic oxidation of hypervalent bis-catecholato silicon compounds as shown in Scheme 25b. Thus, compound **25**–**4** upon oxidation released the desired substituted alkyl radical, and addition onto allyl sulfone **25**–**5** gave the corresponding allylated derivative **25**–**6** in 70% yield.^113^ Olefin **25**–**5** was also used in a decarboxylative allylation of alkyl *N*-acyloxyphthalimides under Ru^II^ photocatalysis. The great advantage of the process was the reaction time since the allylation was completed in a few minutes at room temperature.^189^

**Scheme 26:**
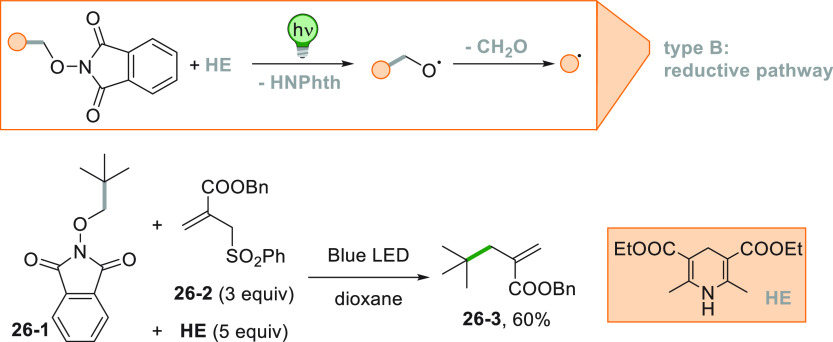
Hantzsch Ester Mediated Photocleavage of *N*-Alkoxyphthalimides

A particular class of phthalimides could be employed with no need of a photocatalyst to promote the reaction. *N*-alkoxyphthalimide (**26**–**1**) is able to form a donor–acceptor complex with electron donor compounds, such as the Hantzsch ester **HE**. Upon excitation, an electron transfer occurred within the complex generating a radical anion, which released an alkoxy radical upon N–O bond cleavage. Loss of formaldehyde formed the desired alkyl radical which reacted with **26**–**2**, to obtain product **26**–**3** in 60% yield (Scheme 26).^127^

**Scheme 27:**
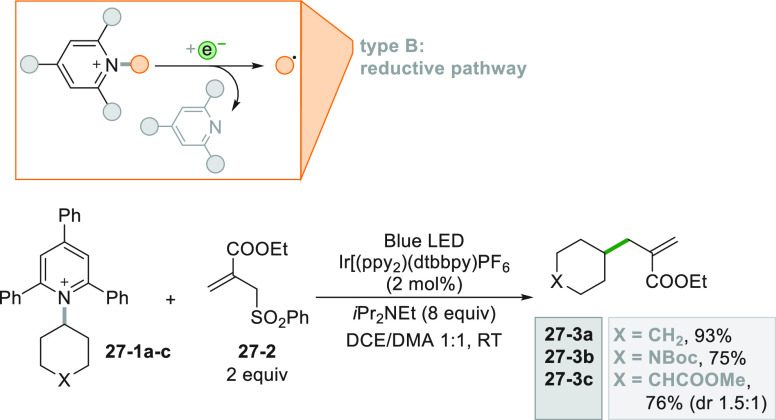
Photocatalyzed Allylation by Using Katritzky Salts

Photocatalyzed reduction of Katritzky salts **27–1a–c** obtained from the corresponding amines (Scheme 27) gave access to the allylated compounds **27–3a–c**. Thus, the monoelectronic reduction of pyridinium salts **27–1a–c** caused the release of the corresponding pyridines along with the substituted cyclohexyl radicals than upon trapping by **27**–**2** efficiently afforded acrylates **27–3a–c**.^190^

**Scheme 28:**
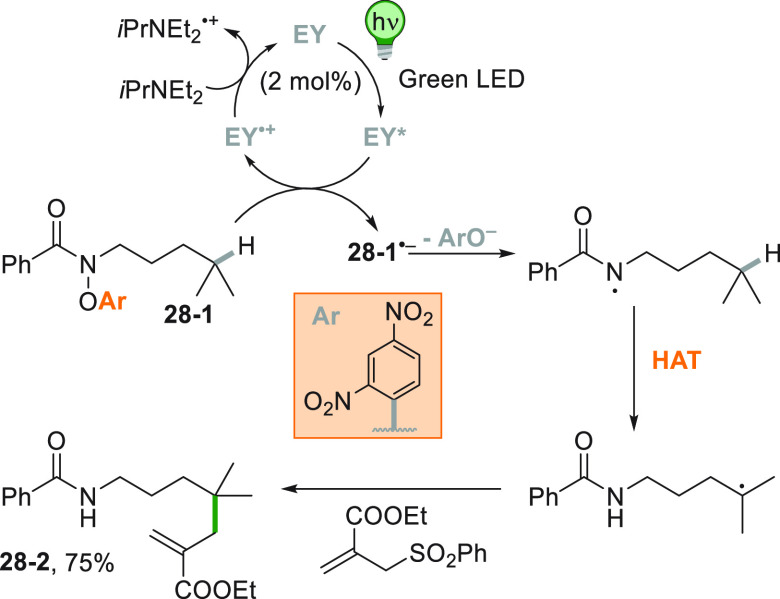
Remote Allylation *via* Amidyl Radicals

A remote allylation under visible light irradiation was devised starting from amide **28**–**1** making use of eosin Y (EY) as the PC (Scheme 28). The excited EY is able to reduce **28**–**1** thanks to the electron-withdrawing capability of the substituted phenoxy group on the nitrogen of the amide. The amidyl radical formed upon fragmentation of **28**–**1**^**•–**^ gave rise to a tertiary radical upon 1,5-HAT, allowing the remote allylation, forming **28**–**2** in 75% yield.^191^

**Scheme 29:**
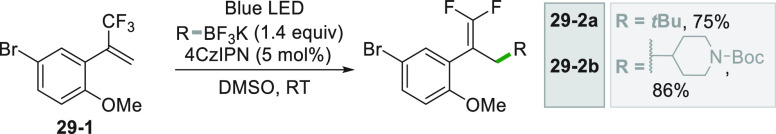
Synthesis of gem-Difluoroalkenes from Alkyl Trifluoroborates

A different approach involved the use of trifluoromethyl-substituted alkenes (e.g., **29**–**1**) that upon addition of the alkyl radical gave access to valuable gem-difluoroalkenes such as **29–2a–b** (Scheme 29). The oxidation of alkyltrifluoroborates was here assured by the organic photocatalyst 4CzIPN, leading to nonstabilized primary, secondary, and tertiary radicals. The defluorinative alkylation resulted from the reduction of the radical adduct, followed by an E1cB-like fluoride elimination.^192^

**Scheme 30:**
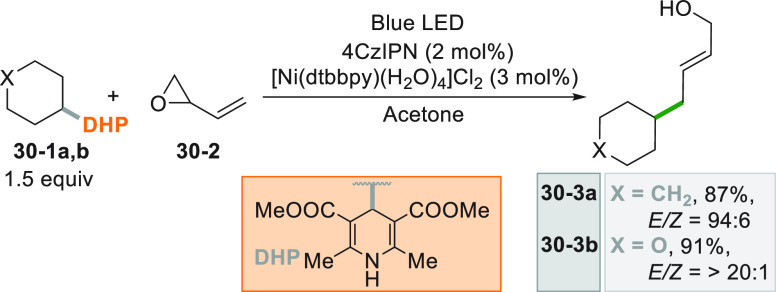
Dual-Catalytic Allylation of Vinyl Epoxides

A dual catalytic approach was designed for valuable allylation using vinyl epoxides as allylating agents (Scheme 30). The mechanism was investigated by quantum mechanical calculations [by DFT and DLPNO–CCSD(T)] and supported an initial complexation of Ni^0^ to **30**–**2** that quickly underwent a S_N_2-like ring opening, followed by the incorporation of the alkyl radical formed by DHP-derived compounds **30–1a,b** into the metal complex. Allyl alcohols **30–3a,b** were then formed by inner sphere C(sp^2^)-C(sp^3^) bond formation from the resulting Ni^III^ complex.^193^

#### 2.1.4 sp^3^–sp^3^ Cross-coupling

**Scheme 31:**
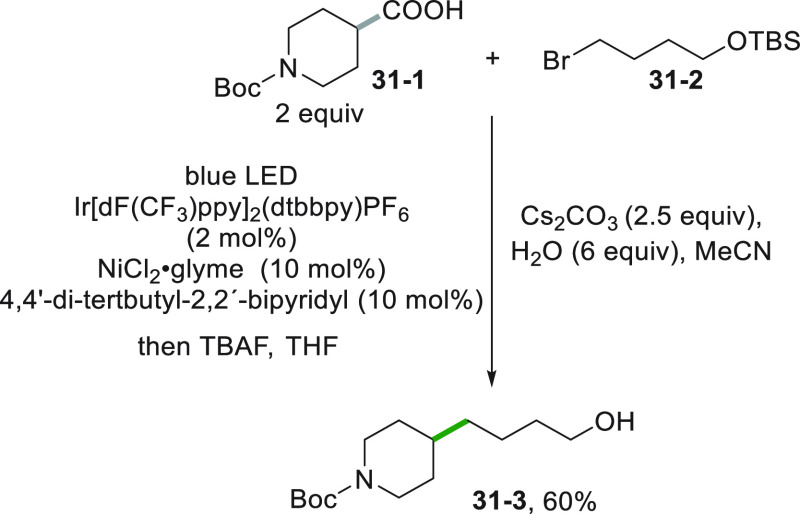
Synthesis of a Precursor of Tirofiban by a Metallaphotoredox Strategy

Another intriguing possibility offered by the photochemical approach to alkyl radicals is the formation of a C–C bond by a sp^3^–sp^3^ cross-coupling reaction. The transformation could lead to novel pathways to interesting targets, as represented by the synthesis of the drug tirofiban in only four steps, starting from easily available compounds. The protocol made use of two consecutive photocatalyzed reactions applying a metallaphotoredox strategy (Scheme 31). The key step is the coupling between carboxylic acid **31**–**1** and alkyl halide **31**–**2**. The halide is first complexed by a Ni^0^ catalyst and the resulting Ni^I^ complex trapped the alkyl radical (obtained by photocatalyzed decarboxylation) to yield a Ni^III^ complex that in turn released the sp^3^–sp^3^ coupled product **31**–**3** after desilylation with TBAF. The desired tirofiban was then obtained by elaboration of **31**–**3** in two subsequent steps.^194^

Another example of a C(sp^3^)–C(sp^3^) cross-coupling process is the reaction between alkylsilicates and alkyl halides. As in the previous case, a dual catalytic Ir/Ni system was required.^195^ The alkyl radical may be likewise generated from an alkyl halide by a halogen atom transfer with a photogenerated silyl radical (from a silanol). The radical that is hence formed could be coupled with another alkyl bromide, e.g., methyl bromide, using a Ni^0^ catalyst to perform valuable methylation reactions.^196^ Aliphatic carboxylic acids were used to form alkyl-CF_3_ bonds via a photocatalyzed reaction making use of Togni’s reagent as the trifluoromethylating agent. The reaction was promoted under visible light irradiation employing an Ir^III^ photocatalyst coupled with a CuI salt. This process tolerates various functionalities including olefins, alcohols, heterocycles, and even strained ring systems.^197^

**Scheme 32:**
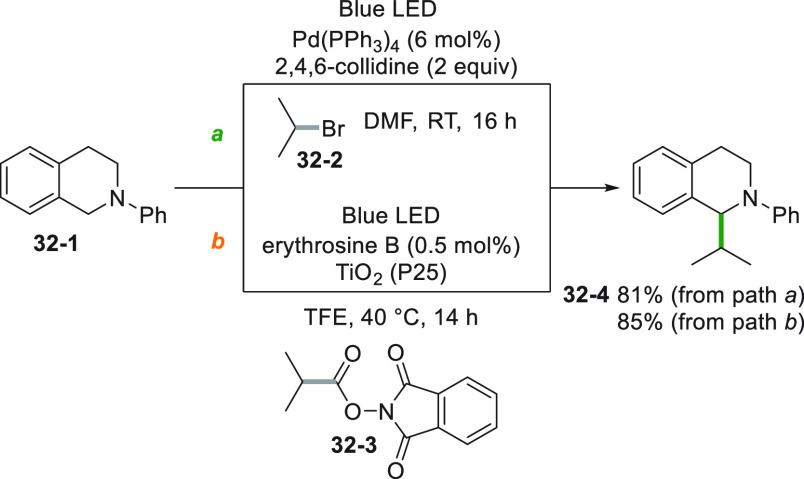
Different Strategies in the Alkylation of *N*-Aryl Tetrahydroisoquinolines

The alkylation of a benzylic position in *N*-aryl tetrahydroisoquinoline **32**–**1** was reported following two different approaches (Scheme 32). The first allowed the reaction of an unactivated alkyl bromide (**32**–**2**) by the excitation of a Pd^0^ complex. Compound **32**–**1** was oxidized in the catalytic cycle, and the resulting α-amino radical coupled with the isopropyl radical to form **32**–**4** in 81% yield (Scheme 32a).^198^ An alternative heavy-metal-free route catalyzed by a dye-sensitized semiconductor is depicted in Scheme 32b. Excitation of an inexpensive dye (erythrosine B) caused the reduction of titanium dioxide that in turn was able to reduce phthalimide **32**–**3** that eventually yielded quinoline **32**–**4**.^199^

#### 2.1.5 Other Reactions

**Scheme 33:**
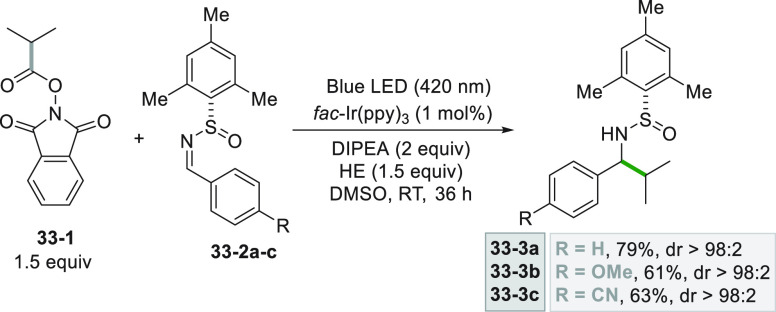
Addition of an Alkyl Radical to Chiral Sulfinimines

In particular cases, a C=N bond can be made sufficiently electrophilic to undergo alkyl radical addition as in the case of *N*-sulfinimines, exploited for the preparation of protected amines. A high degree of diastereoselectivity can be obtained when starting from chiral *N*-sulfinimines (**33–2a–c**, Scheme 33). Thus, the asymmetric addition of an isopropyl radical (formed from derivative **33**–**1**) onto **33–2a–c** allowed the isolation of sulfinamides **33–3a–c** in good yields.^200^

The alkylation of related imines can be carried out by using ammonium alkyl bis(catecholato)silicates as the radical precursors under metal-free conditions adopting 4CzIPN as the POC^201^ or by using potassium alkyltrifluoroborates in the alkylation of *N*-phenylimines.^202^

**Scheme 34:**
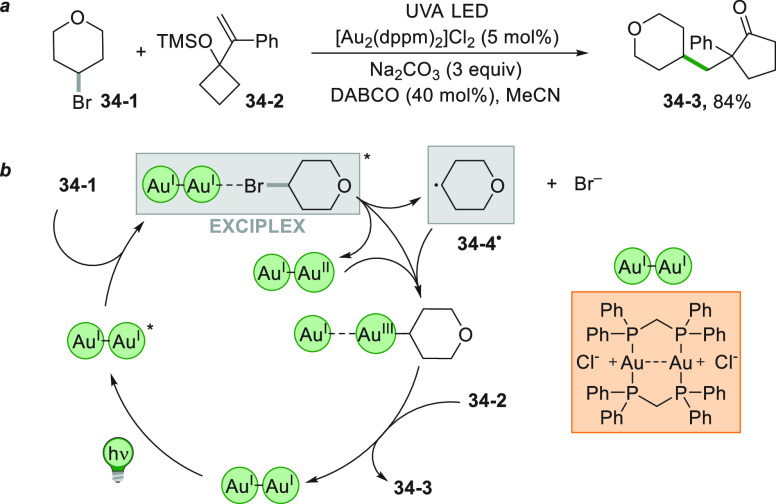
Gold Catalyzed Activation of Bromoalkanes

Another particular case is the alkylative semipinacol rearrangement devised for the synthesis of 2-alkyl-substituted cycloalkanones. The reaction involved the photocatalytic reaction between TMS protected α-styrenyl substituted cyclic alcohol **34**–**2** and the unactivated bromoalkane **34**–**1** (Scheme 34a). The reaction was promoted by the dimeric gold complex [Au_2_(dppm)_2_]Cl_2_. This complex is able to reduce **34**–**1** (ca. −2.5 V vs SCE) despite having an oxidation potential in the excited state considerably lower for the reaction to occur (ca. −1.63 V vs SCE). This can be explained by the formation an inner sphere exciplex between the excited dimeric catalyst and **34**–**1** that promotes the otherwise thermodynamically unfeasible redox process, generating an Au^I^–Au^II^ dimer and **34**–**4**^**•**^. The combination of the latter species formed an Au^III^ complex that induces a semipinacol rearrangement coupled with C(sp^3^)–C(sp^3^) reductive elimination, which furnished **34**–**3** in 84% yield (Scheme 34b).^203^

A similar reaction was later developed starting from cycloalkanol-substituted styrenes and *N*-acyloxyphthalimides under Ir^III^ photocatalysis.^204^

### 2.2 Formation of a C(sp^3^)-C(sp^2^) Bond

#### 2.2.1 Alkenylation

**Scheme 35:**
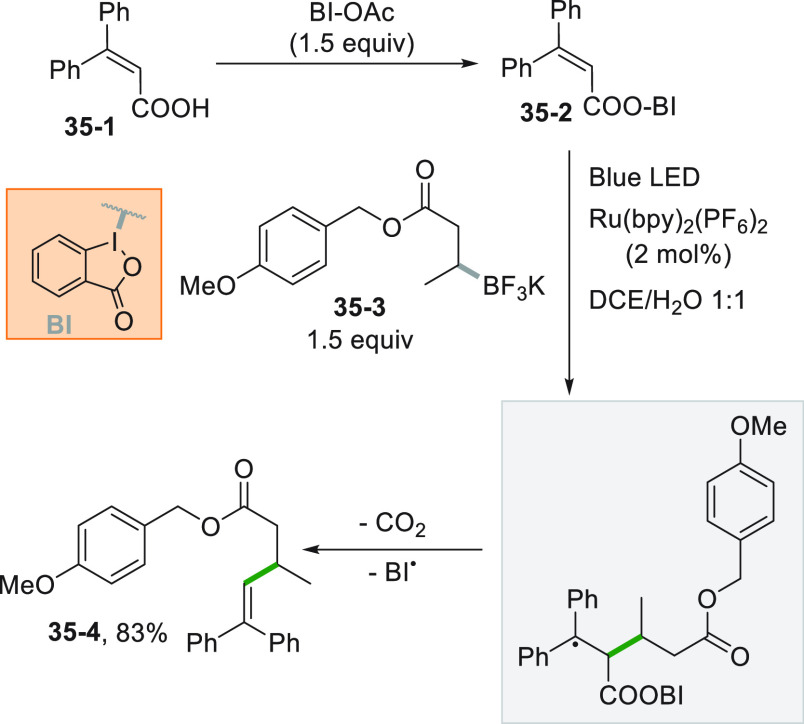
Alkenylations Mediated by Benziodoxole

The reaction between an alkyl radical with a cinnamic acid followed by loss of the COOH group is one of the more common approaches to promote an alkenylation reaction. Thus, the radical formed from salt **35**–**3** attacked the benziodoxole adduct **35**–**2**, synthesized from acrylic acid **35**–**1**. The reaction yielded 83% of the diphenylethylene derivative **35**–**4** upon a deboronation/decarboxylation sequence (Scheme 35).^205^ The benziodoxole moiety gave efficient results in promoting the radical elimination step, while other noncyclic I^III^ reagents were ineffective.

**Scheme 36:**
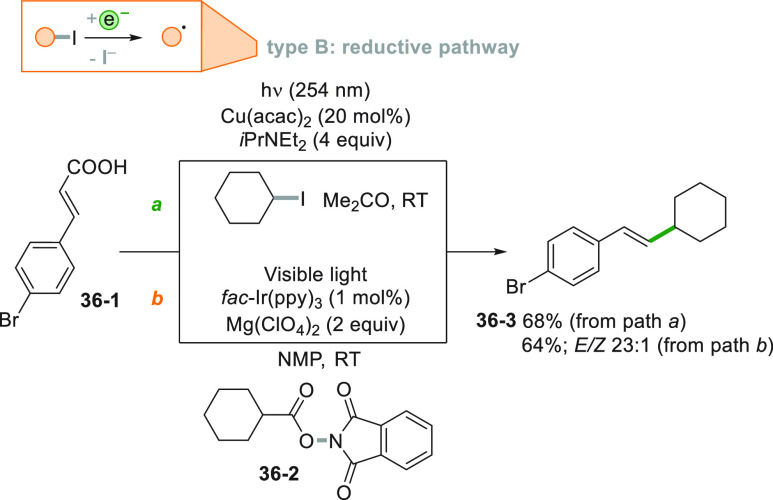
Different Strategies Toward Decarboxylative Alkenylations

Different decarboxylative alkenylations have been reported by changing the radical source and the photocatalyst (Scheme 36). The homolytic cleavage of an alkyl-I bond has been promoted by a Cu^I^ complex and the resulting cyclohexyl radical afforded styrene **36**–**3** in 68% yield upon addition onto cinnamic acid **36**–**1** (path a).^206^ The same product may be formed as well starting from the same substrate by using phthalimide **36**–**2** under visible light irradiation with the help of an Ir^III^^207^ (path b) or a Ru^II^ photocatalyst.^208^ As an additional bonus, the formation of adduct **36**–**3** was obtained with a preferred *E* configuration.

**Scheme 37:**
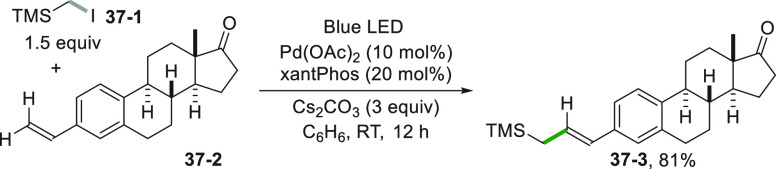
Heck-Like Alkenylation of an Alkyl Iodide

The alkenylation may mimic a Heck reaction as in the visible light-induced Pd-catalyzed reaction between a vinyl (hetero)arene and an α-heteroatom-substituted alkyl iodide or bromide (see Scheme 37). Here, the generation of the radical is induced by the reduction of the TMS-derivative **37**–**1** by the excited Pd^0^ species. Radical addition onto **37**–**2** followed by β-H-elimination from the adduct radical delivered allyl silane **37**–**3** in 81% yield.^209^ Noteworthy, the same reaction did not take place under usual thermal Pd-catalysis.

**Scheme 38:**
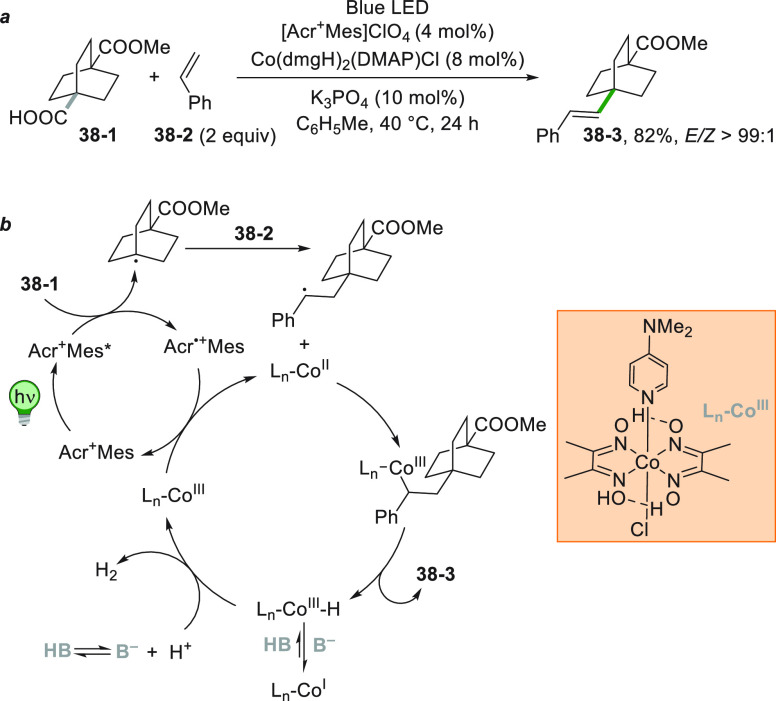
Cobaloxime-Mediated Decarboxylative Coupling of Carboxylic Acids with Styrenes

Alkylation of styrenes could be carried out using an inexpensive palladium source (Pd(PPh_3_)_4_) with no need of any base or classical photocatalyst. The reaction was promoted by visible light, adopting *N*-hydroxyphthalimides as radical sources.^210^ Other visible light Pd promoted alkenylations include the reaction of vinyl arenes with carboxylic acids^211^ or tertiary alkyl halides^212^ as radical precursors. Other metal catalysts, however, were helpful for the substitution of a vinylic hydrogen atom with an alkyl group. In this respect, a dinuclear gold complex was employed for the activation of an alkyl bromide to promote a photocatalyzed Heck-like reaction.^213^ The synergistic combination of a POC and a cobaloxime catalyst promoted the photocatalyzed decarboxylative coupling between **38**–**1** and styrene **38**–**2** to give the alkenylated product **38**–**3** in 82% yield and with a complete *E*/*Z* selectivity as illustrated in Scheme 38.^214^

**Scheme 39:**
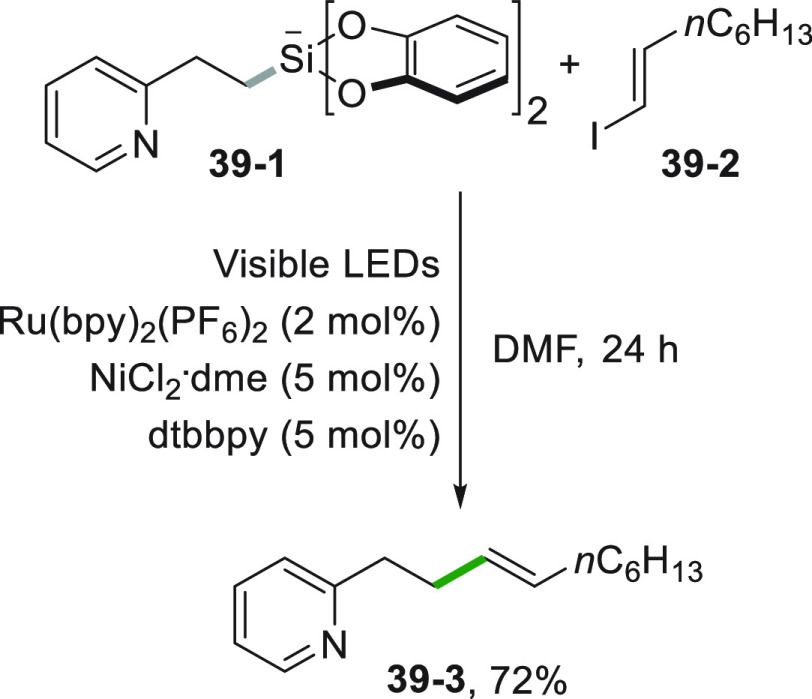
Alkenylation via Alkenyl Iodides

The addition of the alkyl radical may take place even on substituted alkenes *via* an ipso-substitution reaction. An example is shown in Scheme 39 where a vinyl iodide (**39**–**2**) is used for an alkenylation by the reaction with a radical generated from silicate **39**–**1**, obtaining compound **39**–**3**. The Ru^II^ photocatalyst in the dual catalytic system has the role of generating the radical, while the Ni^0^ catalyst activates the C(sp^2^)-I bond.^215^

**Scheme 40:**
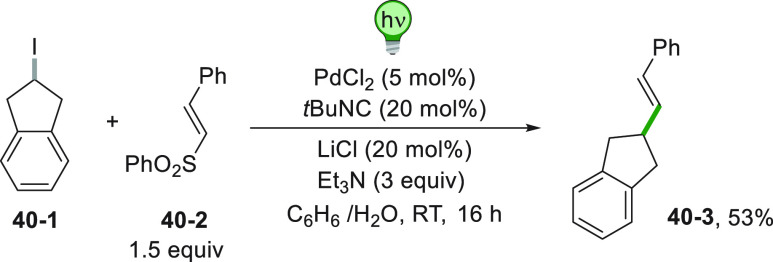
Alkenylation of an Alkyl Iodide with Alkenyl Sulfones

Alkenylation of alkyl iodide **40**–**1** can also take place starting from an alkenyl sulfone (**40**–**2**). Also in this case, an ipso-substitution is central to the novel bond formation and the Pd^0^ catalyst formed the radical by a SET reaction with **40**–**1**. After the addition of the radical onto **40**–**2**, the sequence is completed by the elimination of a sulfonyl radical affording 53% yield of **40**–**3** (Scheme 40).^216^

Alkyl bromides were used in alkenylations by reaction with vinyl sulfones made possible by the photocatalytic generation of silicon centered radical that in turn formed the alkyl radical by a halogen atom transfer reaction.^217^

#### 2.2.2 Acylation

**Scheme 41:**
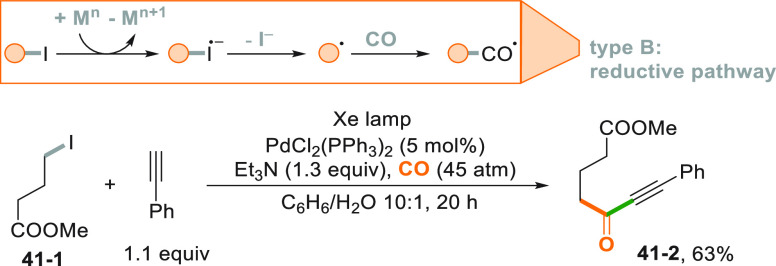
Photocatalyzed Synthesis of Ynones

Acylation owes its importance to the possibility to convert an alkyl radical into a ketone, a reaction that proceeds in most cases with the intermediacy of an acyl radical.^218,219^ A classical approach is based on the homolytic cleavage of an alkyl-I bond followed by carbonylation with CO and reaction with electrophiles of the resulting nucleophilic acyl radical. Scheme 41 illustrates the concept. Irradiation of iodide **41**–**1** with a Xe lamp in the presence of CO (45 atm) and a Pd^0^ complex led to an electron transfer reaction which formed an alkyl radical that, upon carbonylation and addition onto phenyl acetylene, gave ynone **41**–**2** in 63% yield.^220^ The reaction is supposed to proceed via a photoinduced electron transfer from the Pd^0^ catalyst to the iodoalkane, furnishing a Pd^II^ species and the alkyl radical. The carbon-centered radical promptly reacts with CO to generate an acyl radical. The Pd^I^ catalyst intervenes here again to couple the acyl derivative with the alkyne, preserving the triple bond in the final product. This reaction was later applied to the acylation of styrenes to give the corresponding enones.^221^ The electrophilic nature of the alkyne could be exploited if the moiety is placed in the same reagent bearing the iodide. In this case, the first reaction observed was an intramolecular cyclization forming an alkenyl radical which eventually reacted with CO, furnishing an α,β-unsaturated ketone.^222^

**Scheme 42:**
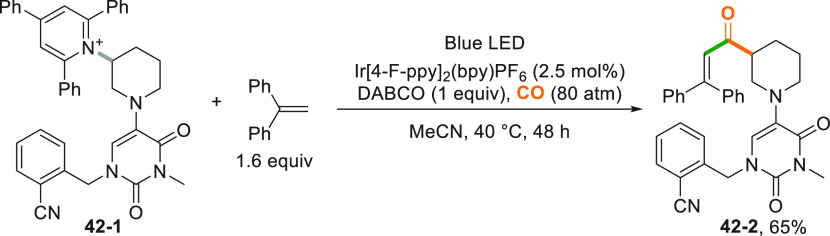
Synthesis of Enones via Photocatalyzed C–N Bond Activation

A reductive step induced the generation of the alkyl radical through an Ir^III^-photocatalyzed C–N bond activation in pyridinium salt **42**–**1** (Scheme 42). Trapping of the alkyl radical with CO followed by addition onto 1,1-diphenylethylene gave access to the Heck-type product **42**–**2** with no interference by the 2,4-dioxo-3,4-dihydropyrimidin-1-yl ring.^223^

**Scheme 43:**
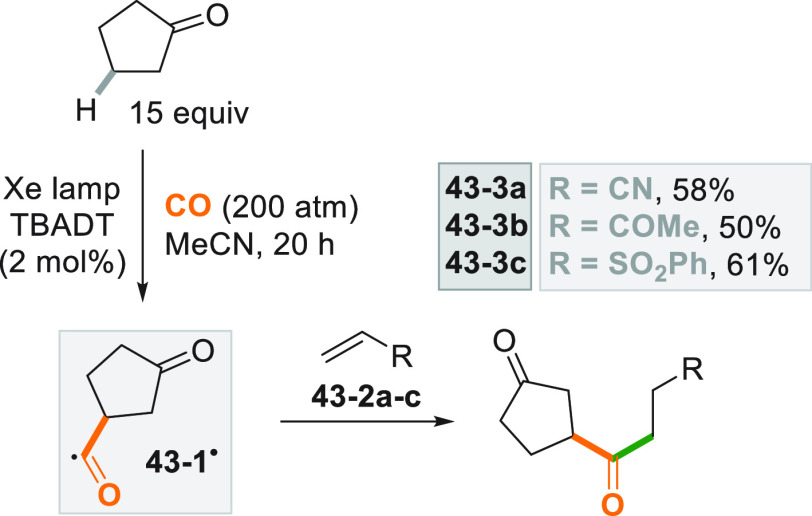
Photocatalyzed Synthesis of Unsymmetrical Ketones

The alkyl radical to be carbonylated was likewise formed starting from a cycloalkane for the preparation of unsymmetrical ketones via radical addition onto Michael acceptors. The reaction proceeded *via* a photocatalyzed decatungstate hydrogen atom transfer reaction^224^ When cyclopentanones were subjected to the photocatalyzed C–H activation, a regioselective β-functionalization occurred. Thus, 1,4-diketones **43–3a–c** were smoothly formed by reaction of the photogenerated acyl radical **43**–**1**^**•**^ onto Michael acceptors **43–2a–c** (Scheme 43).^225^

Unsymmetrical ketones have been likewise formed by carbonylation of alkyl radicals generated from organosilicates by using 4CzIPN as POC under visible-light irradiation.^226^

**Scheme 44:**
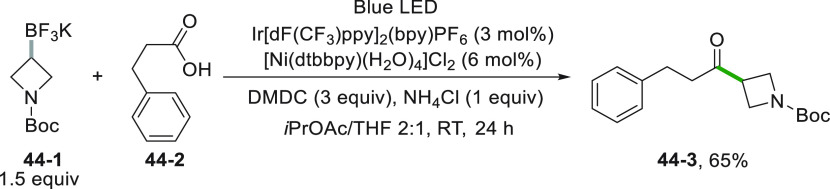
Dual Catalytic Acylation of Alkyl Trifluoroborates

Potassium alkyltrifluoroborates were extensively used for acylation reactions having recourse to a dual photocatalytic system. The unstabilized alkyl radical was generated from trifluoroborate **44**–**1** with the help of an Ir^III^ PC (Scheme 44). Meanwhile, the acid **44**–**2** was converted *in situ* into a mixed anhydride (by reaction with dimethyl dicarbonate, DMDC) that was activated by a Ni^0^ complex. Addition of the alkyl radical onto the resulting complex led to the acylated product **44**–**3**.^227^ In a similar vein, Ir-photoredox/nickel catalytic cross-coupling reactions were devised by using acyl chlorides^228^ and *N*-acylpyrrolidine-2,5-diones^229^ as acylating reagents.

A Ni/Ru, dual-catalyzed amidation protocol was possible thanks to the coupling between an alkylsilicate and an isocyanate. Even in the latter case, the alkyl radical attacked the complex formed between the isocyanate and a Ni^0^ species and, as a result, the mild formation of substituted amides took place.^230^

**Scheme 45:**
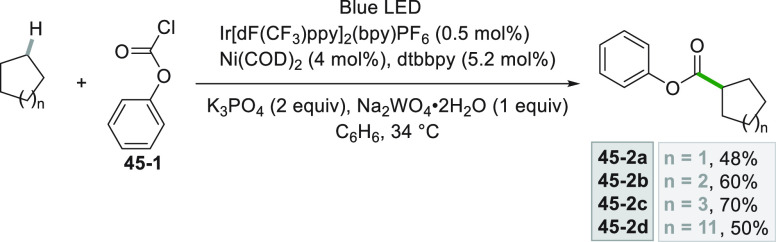
Dual Catalytic Acylation of Cycloalkanes

The acylation of the radical was also exploited for the synthesis of esters. This elegant approach involves the generation of radicals from unactivated C(sp^3^)–H bonds (e.g., in cycloalkanes). The hydrogen abstraction on cycloalkanes was induced by a chlorine atom released from the photocleavage of the complex formed between chloroformate **45**–**1** and a Ni^0^ complex, allowing one to synthesize scaffolds with different ring sizes (**45–2a–d** in Scheme 45).^231^

#### 2.2.3 Minisci-Like Reactions

**Scheme 46:**
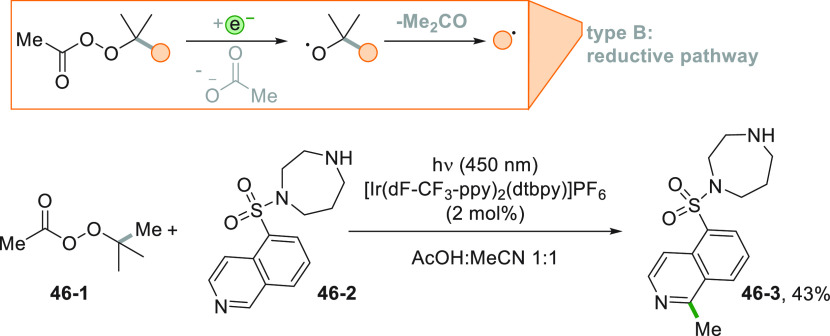
Alkylation of Fasudil

A fundamental transformation for the construction of C(sp^*2*^)-C(sp^*3*^) bond is the Minisci reaction, where the functionalization of heteroaromatics took place by substituting a H atom with an alkyl group. The reaction was widely investigated in the last years and mainly involves the functionalization of a nitrogen-containing heterocycle.^232^ An interesting example is the methylation reported in Scheme 46.^94^ A methyl radical was formed by using a peracetate such as **46**–**1**. The protonation of **46**–**1** by acetic acid facilitates a PCET reduction of the peracetate by the Ir^III^ PC. A double fragmentation ensued, and the resulting methyl radical may attack the protonated form of biologically active heterocycles (e.g., fasudil **46**–**2**) in a mild selective manner to afford **46**–**3** in 43% yield.^94^

**Scheme 47:**
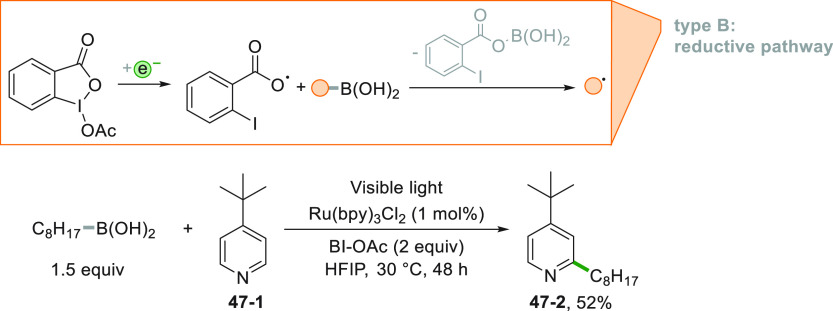
Minisci Reaction by Using Alkyl Boronic Acids

Another approach made use of an alkyl boronic acid as the radical precursor. The process is initiated by the Ru^II^-photocatalyzed reduction of acetoxybenziodoxole (BI-OAc) that liberated the key species ArCOO^•^ (Scheme 47). Upon addition onto an alkyl boronic acid, this ortho-iodobenzoyloxy radical made available the alkyl radical that in turn functionalized pyridine **47**–**1** in position 2 in 52% yield (**47**–**2**, Scheme 47).^233^

The generation of the alkyl radical from boron-containing derivatives was made easier starting from alkyltrifluoroborates. A POC (Acr^+^Mes) is, however, required, but in all cases, the regioselective functionalization of various nitrogen-containing heterocycles was achieved.^234^ A related chemical oxidant-free approach process was later developed where alkyl radicals were formed by merging electro and photoredox catalysis.^235^

**Scheme 48:**
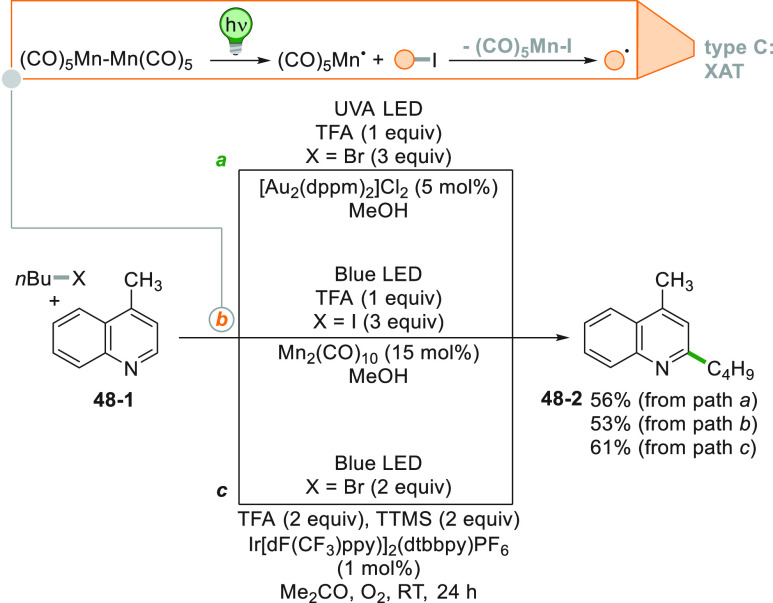
Photocatalyzed Butylation of Lepidine

Alkyl halides are versatile substrates for the photoinduced functionalization (e.g., butylation) of lepidine **48**–**1** (Scheme 48). An uncatalyzed redox process is a rare occurrence here, since alkyl halides reduction is more demanding. This drawback can be overcome by the adoption of a dimeric Au^I^ complex (see Scheme 34) that upon excitation coordinates an unactivated haloalkane promoting an inner sphere PET. This interaction pushes the activation of R-Br despite its larger *E*_red_ with respect to the PC (Scheme 48a).^236^ A different approach promoting the homolytic cleavage of the R-I bond is shown in Scheme 48b. Decacarbonyldimanganese Mn_2_(CO)_10_ was cleaved upon visible light irradiation, and the resulting Mn-based radical was able to abstract the iodine atom from an alkyl iodide thus generating the desired butyl radical. This route was smoothly applied to the late-stage functionalization of complex nitrogen-containing substrates.^237^ Moreover, the activation of alkyl halides may be obtained by the photogeneration of a silyl based radical derived by TTMSS (Scheme 48c, see also Scheme 11). The robustness and the mildness of this approach was witnessed by the broad substrate scope and the compatibility of several functional groups present in the radical.^238^ The use of acidic conditions (required to make the nitrogen heterocycle more electrophilic) may however be avoided. Excited [Ir(ppy)_2_(dtbbpy)]PF_6_ was sufficiently reducing to convert alkyl iodides to alkyl radicals under basic conditions by combining conjugate and halogen ortho-directing effects.^239^

**Scheme 49:**
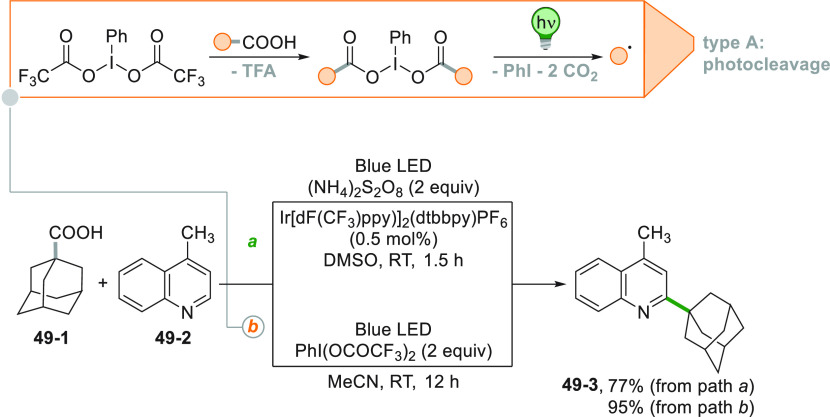
Decarboxylative Minisci Alkylation

In general, lepidine is the preferred substrate to test new ways for the C–H alkylation of heteroarenes. Accordingly, adamantane carboxylic acid **49**–**1** served for the visible light induced synthesis of **49**–**3** starting from lepidine **49**–**2** (Scheme 49). An Ir^III^ PC was adopted to alkylate various nitrogen heterocycles, making use of a large excess of persulfate anion as the terminal oxidant (path a).^240^ The presence of a PC is not mandatory for the adamantylation reaction with (bis(trifluoroacetoxy)-iodo)benzene as starting material. This compound in the presence of a carboxylic acid gave the corresponding hypervalent iodine^III^ reagent that upon irradiation generates the alkyl radical. The TFA liberated in the process was crucial for the activation of the nitrogen heterocycle and adduct **49**–**3** was isolated in 95% yield (path b).^241^

**Scheme 50:**
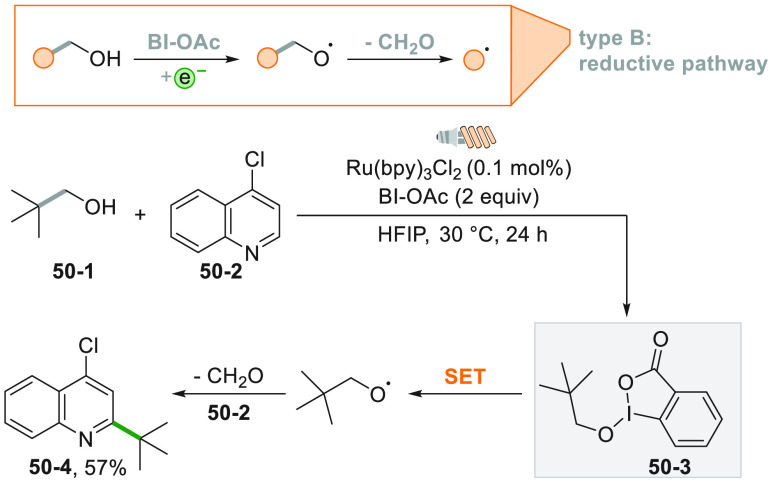
Aliphatic Alcohols as Radical Precursors in Minisci Reaction

Very recently, an interesting approach for the generation of alkyl radicals from the C–C cleavage in alcohols was reported making use of a CFL lamp as irradiation source. The combination of 2,2-dimethylpropan-1-ol (**50**–**1**) with benziodoxole acetate (BI-OAc) gave adduct **50**–**3**. Photocatalytic reduction of compound **50**–**3** released and alkoxy radical that upon fragmentation formed a *t*butyl radical that reacted with *N*-heteroarene **50**–**2** to form **50**–**4** in 57% yield (Scheme 50).^130^

**Scheme 51:**
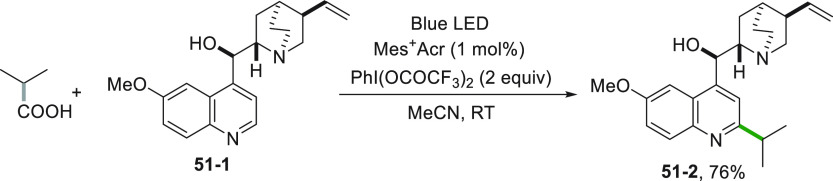
Alkylation of Quinine

The use of hypervalent iodine^III^ in promoting the decarboxylation of R-COOH was effective in the derivatization of drugs or drug-like molecules. As a result, the quinine analogue **51**–**2** was formed in a 76% yield from quinine **51–1,** utilizing Acr^+^Mes as the POC (Scheme 51).^242^

Azoles can be adamantylated starting from adamantane carboxylic acid by a dual catalytic approach (Acr^+^Mes as the POC and [Co(dmgH)(dmgH_2_)Cl_2_] as the cocatalyst)^243^ or simply C_2_-alkylated under photoorganocatalyzed conditions.^244^

**Scheme 52:**
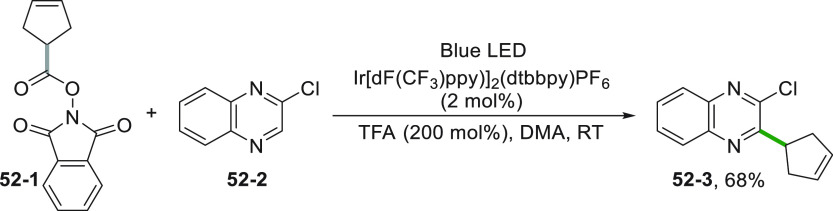
Cyclopentenylation of 2-Chloroquinoxaline

The photocatalyzed reduction of *N*-(acyloxy)phthalimide **52**–**1** induced by an Ir^III^* complex is an alternative approach for the functionalization of *N*-heterocycles such as 2-chloroquinoxaline **52**–**2** to form the cyclopentenyl derivative **52**–**3** (Scheme 52).^245^

**Scheme 53:**
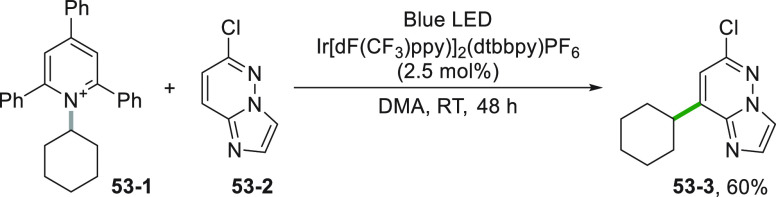
Functionalization of 6-Chloroimidazo[1,2-*b*]pyridazine

The reductive pathway is feasible even when the generation of the alkyl radical was carried out starting from the redox-active pyridinium salt **53**–**1**. In this case, the obtained cycloalkyl radical gave a regioselective addition onto 6-chloroimidazo[1,2-*b*]pyridazine **53**–**2** to yield **53**–**3** under mild conditions (Scheme 53).^70^

**Scheme 54:**
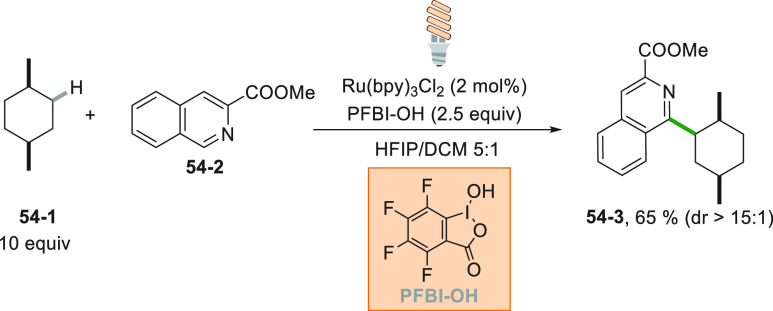
PFBI–OH Mediated Minisci Reaction

The alkyl radical could be formed even from simple hydrocarbons via hydrogen atom transfer reaction. A valuable example is reported in Scheme 54. The hypervalent iodine oxidant PFBI–OH is reduced by an excited Ru^II^ complex generating a carbonyloxy radical that acted as hydrogen atom abstracting agent. Functionalization of isoquinoline **54**–**2** by the resulting radical (derived from **54**–**1**) afforded adduct **54**–**3** in 65% yield (>15:1 dr).^246^ The high selectivity observed in the functionalization of **54**–**1** was ascribed to the slow addition of the tertiary alkyl radical possibly formed onto **54**–**2**.^246^ The direct (rather than indirect) C–H cleavage in cycloalkane was possible by using decatungstate anion as PC. Various nitrogen-containing heterocycles were then easily derivatized even under simulated solar light irradiation.^247^

**Scheme 55:**
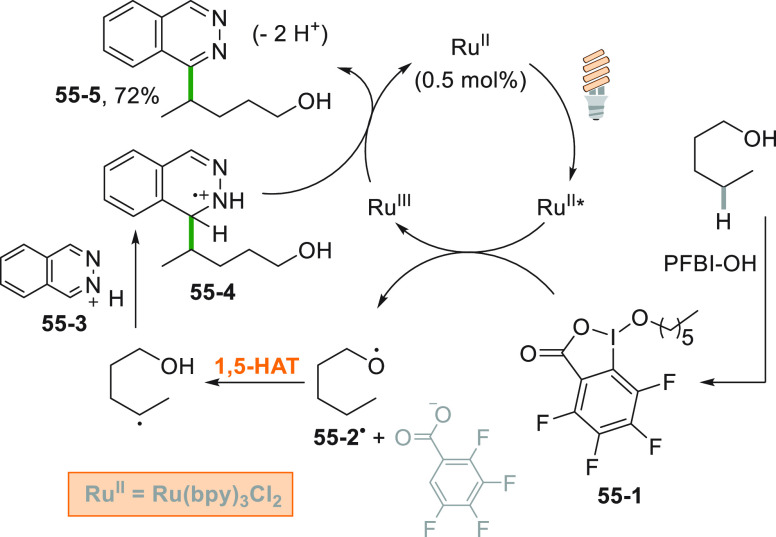
Remote C(sp^3^)–H Heteroarylation of Alcohols

PFBI–OH was likewise used for the remote C(sp^3^)–H heteroarylation of alcohols (Scheme 55). As an example, the reaction of pentanol with PFBI–OH gave adduct **55**–**1** that was reduced by the photocatalyst releasing the alkoxy radical **55**–**2**^**•**^. 1,5-HAT and addition onto protonated phthalazine **55**–**3** afforded adduct **55**–**4** and the functionalized heterocycle **55**–**5** from it in 72% yield after sequential oxidation and deprotonation.^248^

**Scheme 56:**
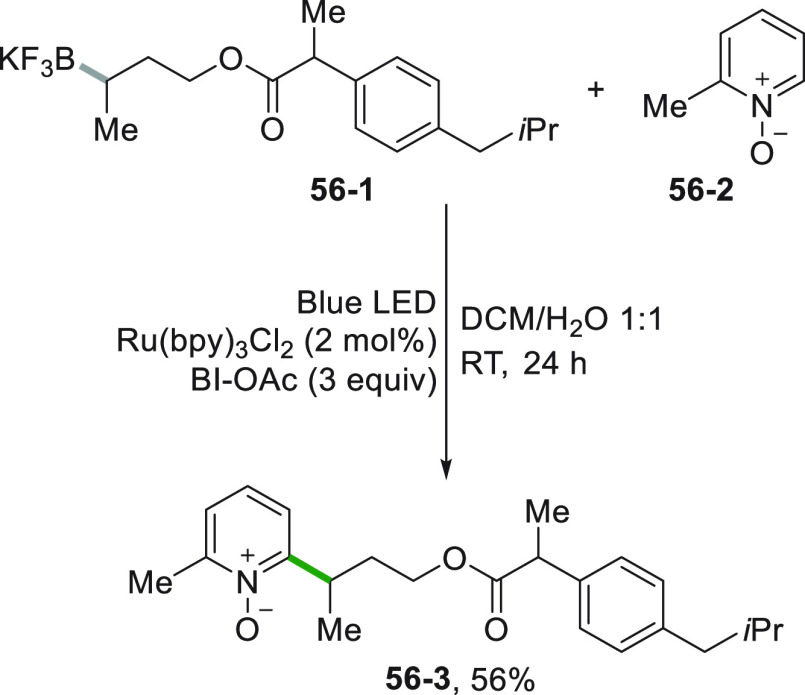
Minisci Alkylation of Pyridine Oxides

As previously stressed, an acid is often required for an efficient Minisci-like reaction. To overcome this problem the alkylation may be carried out on the corresponding *N*-oxide derivatives as it is the case of pyridine *N*-oxides (**56**–**2**, Scheme 56). The radical is generated from a trifluoroborate salt (**56**–**1**) and the alkylation is regioselective in position 2 (forming compound **56**–**3**).^249^ The process is efficient thanks to the photocatalytic degradation of BI-OAc that promoted a hydrogen abstraction, operated by the resulting carbonyloxy radical, on the Minisci radical cation adduct.

**Scheme 57:**
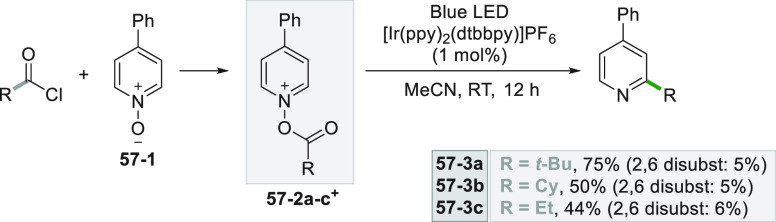
Decarboxylative Alkylation of Heterocycles

On the other hand, the pyridine *N*-oxide **57**–**1** can be acylated *in situ* with suitable acyl chlorides to furnish the electron-poor **57–2a–c**^**+**^ derivatives. Photocatalytic reduction of these intermediates leads to the generation of alkyl radicals prone to attack the pyridine nucleus itself in the ortho position resulting in a decarboxylative alkylation (**57–3a–c**, Scheme 57).^122^

#### 2.2.4 Ipso-Substitution Reactions

The forging of an alkyl-sp^2^ bond (e.g., an alkyl-Ar bond) is undoubtfully one of the most crucial goals pursued by a synthetic organic chemist. Alkyl radicals generated via different mild routes can be successfully employed for the arene *ipso* functionalization, given the presence of a suitable group X on the (hetero)aromatic ring that directs the selective formation of a new Ar–C bond at the expense of an Ar-X bond. Dual catalysis (with the help a Ni-based complex) is one of the preferred approaches.

**Scheme 58:**
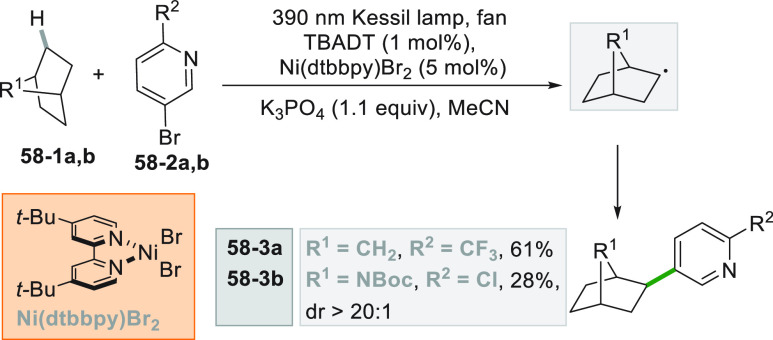
Dual TBADT-Ni Catalysis for the Synthesis of Pyridyl-Functionalized Bicycles

In a recent example, the hydrogen atom transfer ability of the excited TBADT catalyst (see also Scheme 12) is used to form an alkyl radical starting from different aliphatic moieties (see Scheme 58).^250^ The combined action of the tungstate anion and the nickel catalyst (Ni(dtbbpy)Br_2_) allowed the coupling of (hetero)aromatic bromides with unactivated alkanes, overcoming their high bond dissociation energies (ca. 90–100 kcal/mol) and low acidities. Both linear (41–56% yield) and cyclic (57–70% yield) alkanes could be functionalized with a vast range of competent partners. Interestingly, the radicals are generated preferentially on the less sterically demanding secondary carbons in alkanes, affording a remarkable selectivity.

The scope of this method could be proved by the functionalization of natural products and drugs, such as in the preparation of the bicyclic derivative **58–3a** (61% yield) and the *N*-Boc protected epibatidine alkaloid **58–3b** (28%, Scheme 58).^250^ A very similar approach was later reported for the dual photocatalytic formation of an Ar–C bond starting from aryl bromides and cycloalkanes.^251^

**Scheme 59:**
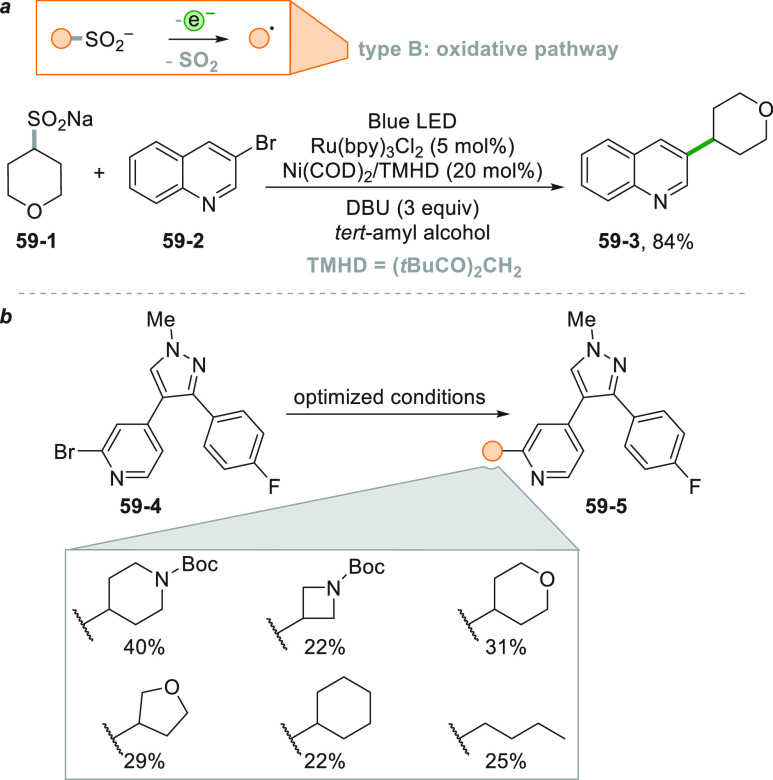
Dual Catalytic Cross-Coupling of Aryl Bromides with Alkyl Sulfinates

Another dual-catalytic approach allowed the coupling reaction of aryl bromides (**59**–**2**, Scheme 59) and alkyl sulfinates (**59**–**1**), in the presence of Ni(COD)_2_ and tetramethylheptanedione (TMHD, Scheme 59a) to give **59**–**3** in 84% yield under air.^104^ The photogenerated radical was trapped by the Ni complex that mediated the coupling with the aryl halide **59**–**2**. The method was then applied to the synthesis of **59–5,** selective ATP-competitive inhibitors of the casein kinase 1δ, an enzyme related to the regulation of the circadian rhythm (Scheme 59b).^104^

**Scheme 60:**
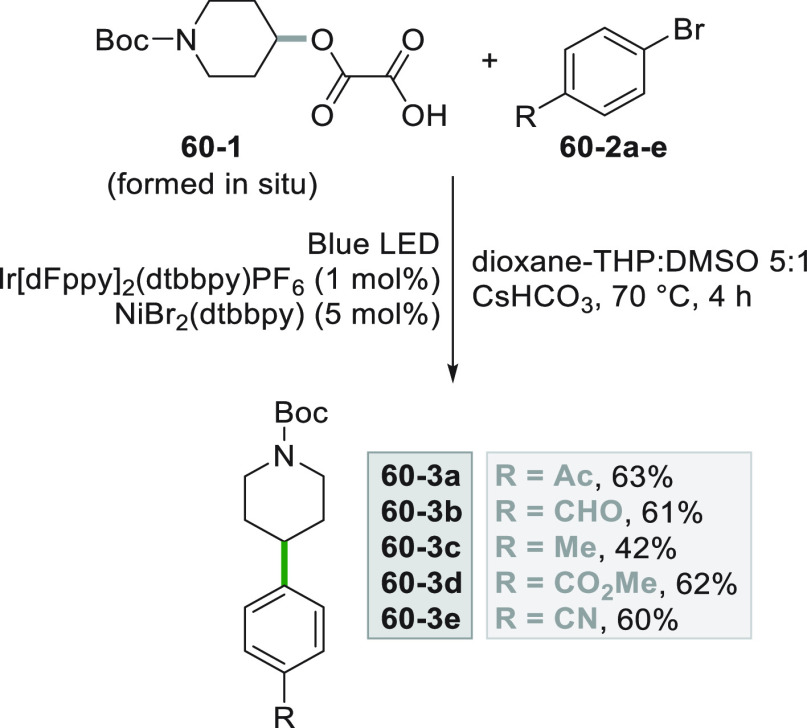
Coupling of Alkyl Oxalates with Aryl Bromides

A very similar strategy to access C(sp^3^) radicals involves the photoredox induced cleavage of alkyl oxalate **60**–**1**, starting from the corresponding alcohols (see Scheme 60, see also Scheme 3).^252^ The rapid *in situ* formation of the oxalate (without purification) was followed by the metallaphotoredox sequence based on Ni catalysis, allowing to obtain the C(sp^2^)-C(sp^3^) coupling to give derivatives **60–3a–e** in good yields.

**Scheme 61:**
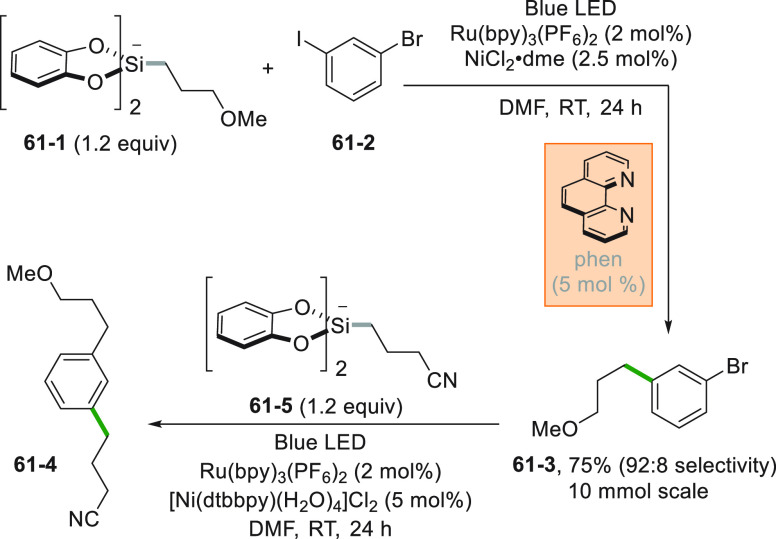
Consecutive Functionalization of Bromo-Iodo Arenes with bis-Catecholato Silicates

The advantage of the use of potassium and ammonium bis-catecholato silicates relies in the smooth generation of unstabilized primary and secondary alkyl radicals to be engaged in dual catalysis.^253,254^ An example is the consecutive functionalization of bromo(iodo)arene **61**–**2** (Scheme 61, see also Scheme 25) for the preparation of **61**–**4** where the radical (from **61**–**1**) is trapped by Ni^0^ (stabilized by a phenanthroline ligand). The synthesis of **61**–**3** can be achieved in high yields on 10 mmol scale with reduced effect on yield (75%) and selectivity (98%). The crude bromide **61**–**3** was further functionalized by a second Ni/photoredox cross-coupling of the alkylsilicate **61**–**5**, affording product **61**–**4** in 66% yield.^255^ The procedure was extended successfully to alkyl triflates, tosylates and mesylates,^256^ and to brominated borazaronaphthalene cores.^257^ The latter approach was crucial to access previously unknown isosteres of azaborines.

**Scheme 62:**
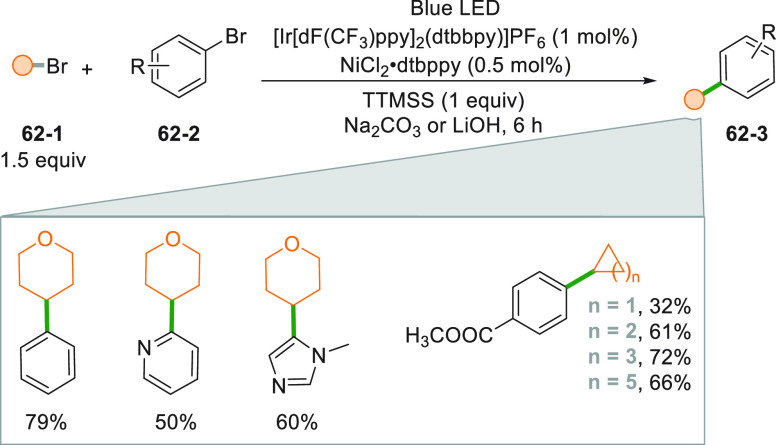
Ir/Ni Complex Mediated Coupling Between Alkyl and Aryl Bromides

The action of a silyl radical on an alkyl bromide **62**–**1** forms an alkyl radical that, again with the help of a Ni based catalyst, reacted with aryl bromides **62**–**2** (Scheme 62, see also Scheme 11). The scope of products **62**–**3** that can be obtained is varied and includes both aromatic and heteroaromatic substrates, along with cycloalkanes of different size.^71^

**Scheme 63:**
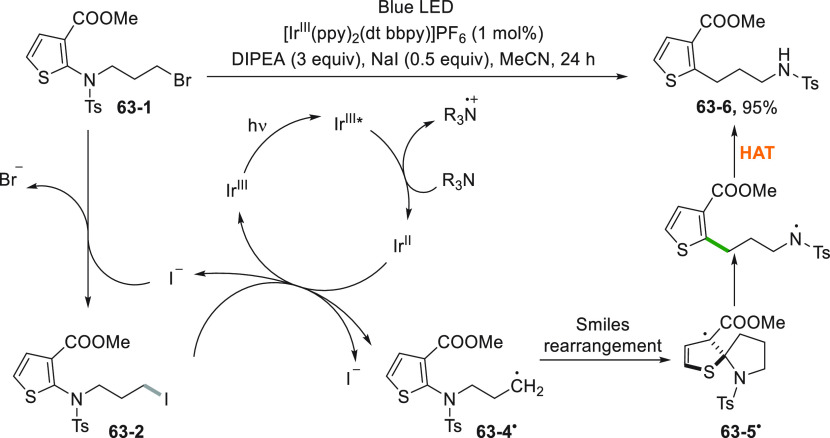
Photocatalyzed Smiles Rearrangement

A peculiar case is when the ipso-substitution took place via a radical rearrangement such as shown in Scheme 63.^258^ Thus, the heteroaromatic sulfonamide **63**–**1** was subjected to the Finkelstein reaction, obtaining the corresponding iodide **63**–**2** that acted as the source of radical able to induce a Smiles rearrangement via **63**–**5**^**•**^. Intermediate **63**–**5**^**•**^ has lost its aromaticity; however, the radical has become tertiary, gaining further stabilization from the ester group nearby. Restoration of the aromaticity is followed by a presumable hydrogen atom transfer to obtain compound **63**–**6** in 95% yield.^258^

**Scheme 64:**
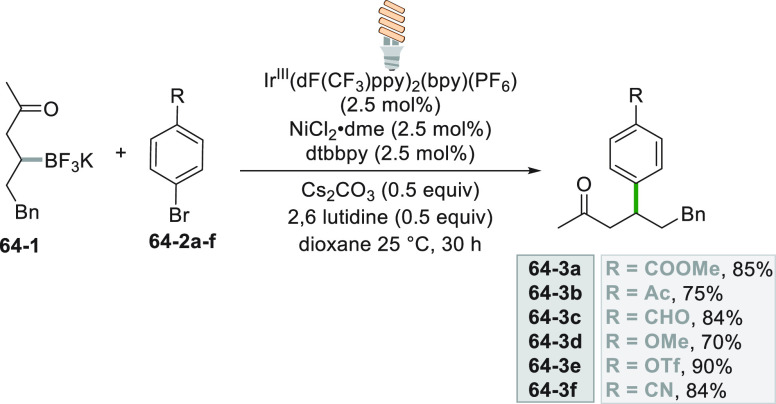
Photoredox/Nickel Dual Catalytic Coupling of β-Trifluoroboratoketones with Aryl Bromides

Dual photoredox/nickel catalysis was successfully applied to couple β-trifluoroboratoketones **64**–**1** with aryl bromides **64–2a–f** (Scheme 64, see also Scheme 4). Arylated compounds **64–3a–f** were efficiently prepared with substituents of different electronic nature on the aryl ring.^259^

**Scheme 65:**
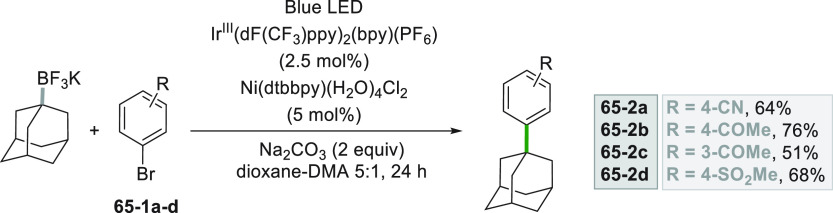
Adamantylation of Aryl Bromides

Potassium tetrafluoroborate salts have been applied to generate secondary alkyl radicals via Ir photocatalysis coupled with Ni.^260^ However, they were found to be likewise suitable for cross-coupling reactions devoted to the forging of quaternary carbon centers (Scheme 65) without the need of using reactive organometallic species.^261^ In the adamantylation of bromides **65–1a–d** better yields were obtained when the aryl ring was substituted with electron-withdrawing groups.

**Scheme 66:**
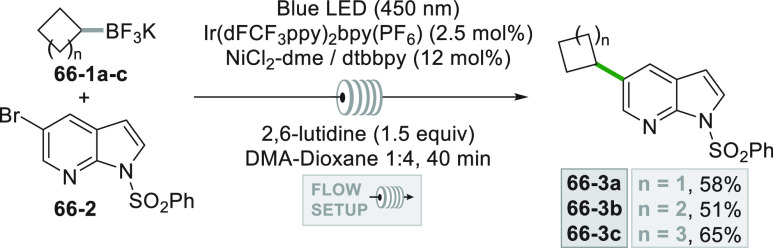
Functionalization of 7-Azaindole Pharmacophores in Flow

An interesting application of this synthetic strategy is the functionalization of 7-azaindole pharmacophores with cycloalkyl scaffolds to improve the drug likeness of the azaindole core structure. Different potential drug candidates (**66–3a–c**, Scheme 66) were prepared via dual photocatalysis in a flow setup varying the dimension and substitution of the ring.^262^

**Scheme 67:**
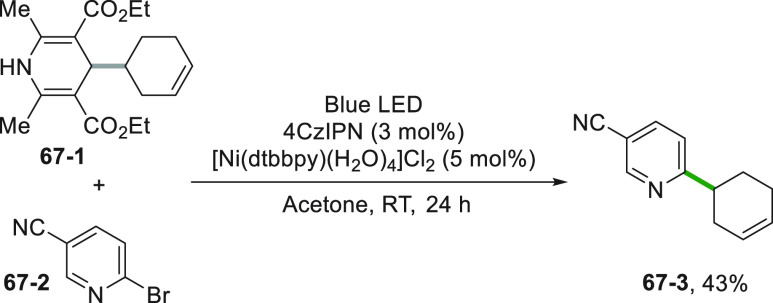
Coupling of DHP-Cyclohexene with Cyanobromopyridine

In a similar way, a DHP-functionalized cyclohexene **67**–**1** was used to generate a secondary alkyl radical. In this case, the authors promoted the oxidation of **67**–**1** by using the strongly oxidizing 4CzIPN photocatalyst. Coupling with bromopyridine **67**–**2** gave substituted pyridine **67**–**3** in moderate yields (Scheme 67, see also Scheme 7).^118^

**Scheme 68:**
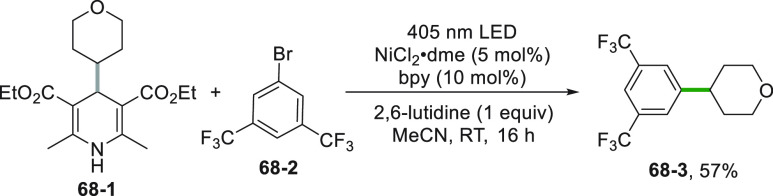
Photocatalyst-Free Activation of DHPs

DHP-derivatives (**68**–**1**) may be used in ipso-substitution reaction even in the absence of a photocatalyst (Scheme 68). Violet light LED illumination directly excited didehydropyridine **68**–**1**, fueling electrons to the Ni^II^ species which formed the catalytic competent Ni^0^ along with the desired radical by the fragmentation of **68**–**1**^**•+**^. Noteworthy, the alkyl aromatic **68**–**3** was then formed where the use of electron-withdrawing groups on the ring contributes to the good yields of the process.^263^

### 2.3 Formation of a C(sp^3^)-C(sp) Bond

#### 2.3.1 Cyanation

**Scheme 69:**
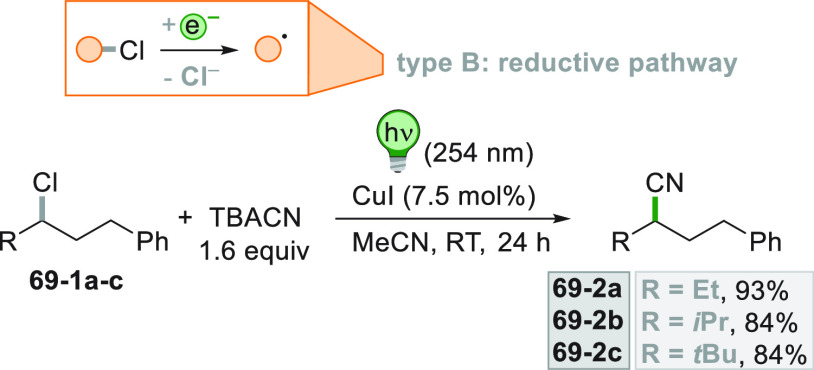
Copper^I^-Mediated Synthesis of Nitriles

Alkyl radicals have been used for the synthesis of alkyl nitriles by using different cyanide sources. The cyanation may be carried out in the presence of cyanide anion as tetrabutyl ammonium salt (TBACN). The C–C bond formation here may be carried out under very mild conditions by using the inexpensive precatalyst CuI, starting from unactivated alkyl chlorides (e.g., **69–1a–c**, Scheme 69). Probably, a Cu^I^-cyanide adduct is the species that was excited and engaged an electron transfer reaction with the alkyl halide to form a Cu^II^-cyanide adduct. This intermediate combines with the alkyl radical formed to release nitriles **69–2a–c**. The Cu^I^-halide complex formed in the reaction restores the initial photocatalyst by exchange with the cyanide anion.^264^

TMSCN was instead used for the remote δ-C(sp^3^)-H cyanation of alcohols under Ir/Cu-photocatalyzed conditions. The reduction of an *N*-alkoxypyridinium salt generated an alkoxy radical that upon intramolecular 1,5-HAT formed an alkyl radical that is cyanated with the help of the copper catalyst.^265^

**Scheme 70:**
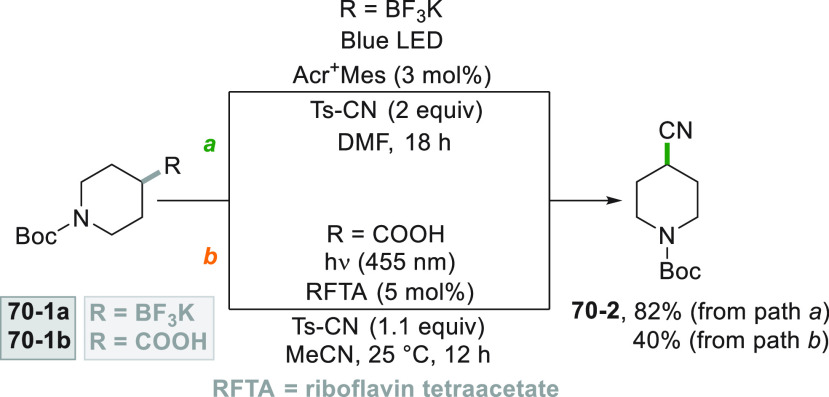
Cyanation of (a) Trifluoroborates and (b) Carboxylic Acids

A typical cyanation procedure, however, makes use of tosyl cyanide as cyanating agent. Thus, the radical obtained by oxidation of trifluoroborate **70–1a** (by excited Acr^+^Mes)^266^ or acid **70–1b** (by riboflavin tetraacetate RFTA)^267^ was trapped by tosyl cyanide to afford nitrile **70**–**2** by a substitution reaction (Scheme 70).

A related Ru^II^-photocatalyzed cyanation employing Ts-CN starting from alkyl trifluoroborates but requiring BI-OAc as a mild oxidant has been likewise reported.^268^

**Scheme 71:**
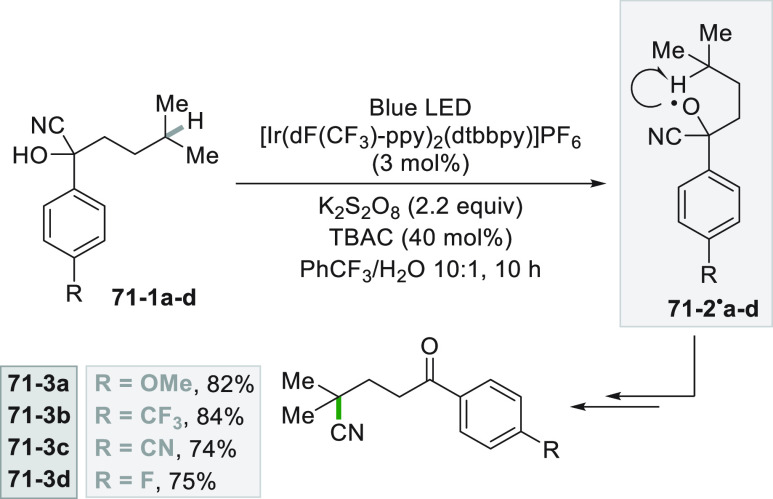
Photocatalyzed Cyano Migration in Cyanohydrines

An elegant way to forge an alkyl-CN bond required the photocatalyzed elaboration of cyanohydrines **71–1a–d**. At first, the interaction of the OH group with the sulfate anion (generated by the decomposition of persulfate anion) allowed its oxidation by a proton-coupled electron transfer (PCET) process promoted by an *in situ* formed Ir^IV^ species. Alkoxy radicals **71**–**2**^**•**^**a–d** were then formed and promoted a regioselective cyanation of remote C(sp^3^)–H bonds by a 1,5-HAT followed by cyano migration to form cyanoketones **71–3a–d** (Scheme 71).^269^

#### 2.3.2 Alkynylation

**Scheme 72:**
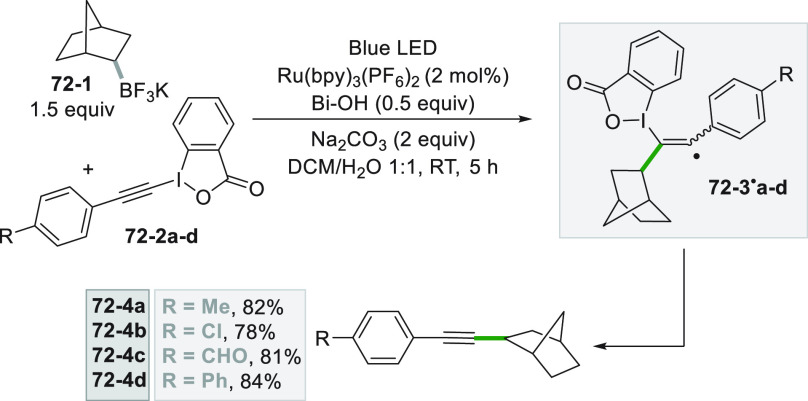
Alkynylation of Alkyl Trifluoroborates

Direct alkynylation of photogenerated alkyl radicals could be accomplished utilizing a reagent or catalyst that activates the alkyne moiety, making it more prone to the forging of a novel C(sp^3^)–C(sp) bond. One of the first strategies that were employed made use of benziodoxole-functionalized alkynes to promote the reaction with the alkyl radical.^270^ A representative case is illustrated in Scheme 72. [Ru(bpy)_3_](PF_6_)_2_ promoted the alkyl radical formation from trifluoroborate salt **72**–**1** that upon addition onto the alkynyl derivatives **72–2a–d** induced the alkynylation via the intermediacy of vinyl radicals **72**–**3**^**•**^**a–d**. This deboronative alkynylation strategy could be performed in neutral DCM:water (1:1) at room temperature, giving access to the alkynylation of primary, secondary, and tertiary derivatives. To further prove the mildness of the conditions used, the authors carried out the reaction in PBS at pH 7.4 in the presence of biomolecules such as amino acids, but also single-stranded DNA and proteins (e.g., bovine serum albumin), obtaining satisfactory yields ranging from 68 to 86% of selectively alkynylated product.^270^

The nature of the substituents on the alkynylbenziodoxole reagent were proved to determine the outcome of the alkynylation process. The electron-rich compounds performed better in the photocatalyzed transformation, both as radical acceptor and oxidative quencher of the Ru^II^* photocatalyst.^271^

**Scheme 73:**
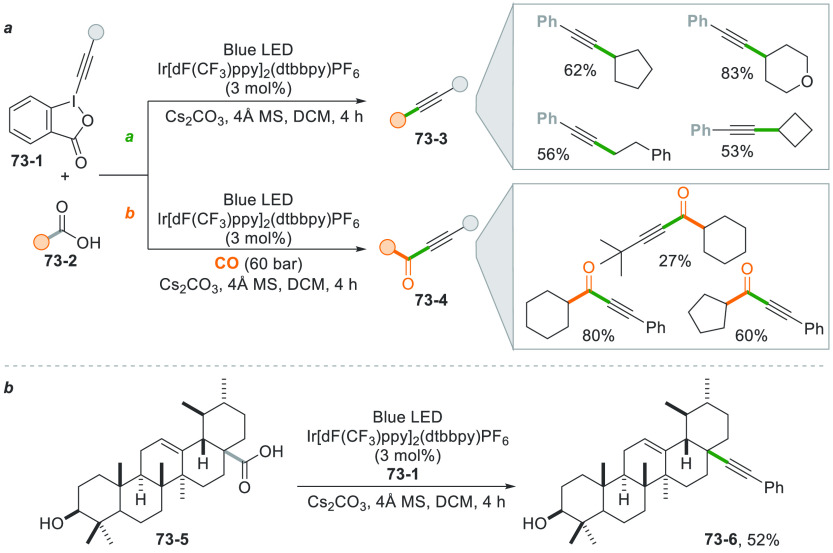
Ir^III^-Catalyzed Alkynylation of Carboxylic Acids

A similar strategy to the one mentioned before consists in the Ir^III^-photoredox-catalyzed alkynylation of carboxylic acids **73**–**2** (see Scheme 73a, path a).^272,273^ In this case benziodoxole derivatives **73**–**1** were again used to activate the sp carbon of the alkyne to the radical attack, affording good yields of products **73–3.** Following these results, they developed a reaction to synthesize ynones **73**–**4** utilizing the same reaction conditions in the presence of gaseous CO (see Scheme 73a, path b and Section 2.2.2). Gram-scale reactions and late-stage functionalization of natural terpenoids such as ursolic acid (**73**–**5**, Scheme 73b) were likewise reported.^273^

**Scheme 74:**
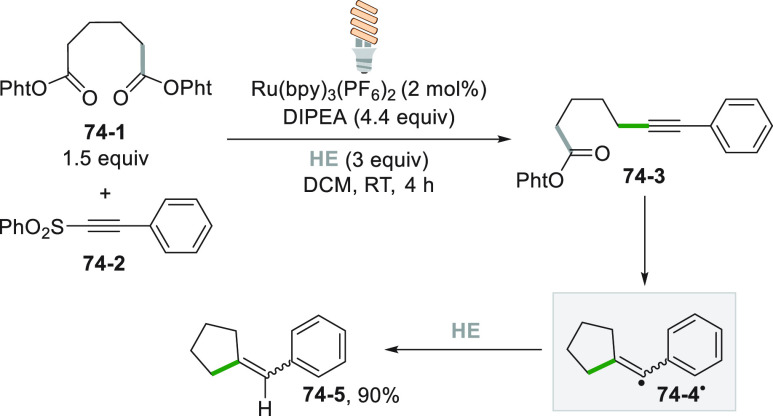
Cascade Double Alkynylation of Functionalized Adipic Acids

Alkynyl sulfones were extensively employed as alkynylating reagents, with a mechanism like the one described in Scheme 72. Alkynyl phenyl sulfone was used in combination with *N*-acyloxyphthalimide derivatives as radical precursors in a Ru^II^-photocatalyzed reaction that gave direct access to TIPS-substituted alkynes.^274^*N*-Phthalimidoyl oxalates and tolyl alkynyl sulfones were found to be competent for the reaction (even for the preparation of internal alkynes having quaternary carbons),^275,276^ the latter even in combination with pyridinium salts as radical precursors.^277^ The consecutive photoredox decarboxylative coupling of doubly functionalized adipic acid derivatives with alkynyl phenyl sulfones induced the cascade formation of interesting cyclic derivatives with an exo double bond (Scheme 74). In this case, compound **74**–**1** underwent two efficient consecutive photoredox decarboxylative couplings leading first to alkyne **74**–**3** that it was subjected to radical cyclization to form radical **74**–**4**^**•**^ and styrene **74**–**5** from it.^278^ The authors reported the formation of five-membered rings via the consecutive formation of two C–C bonds, along with one example showing the application to the synthesis of six-membered derivatives (31% yield).^278^

In rare instances alkynyl bromides could be used as sp counterpart in the radical addition of alkyl derivatives obtained from the oxidative decomposition of various Hantzsch esters under visible light conditions promoted by 4CzIPN.^279^

**Scheme 75:**
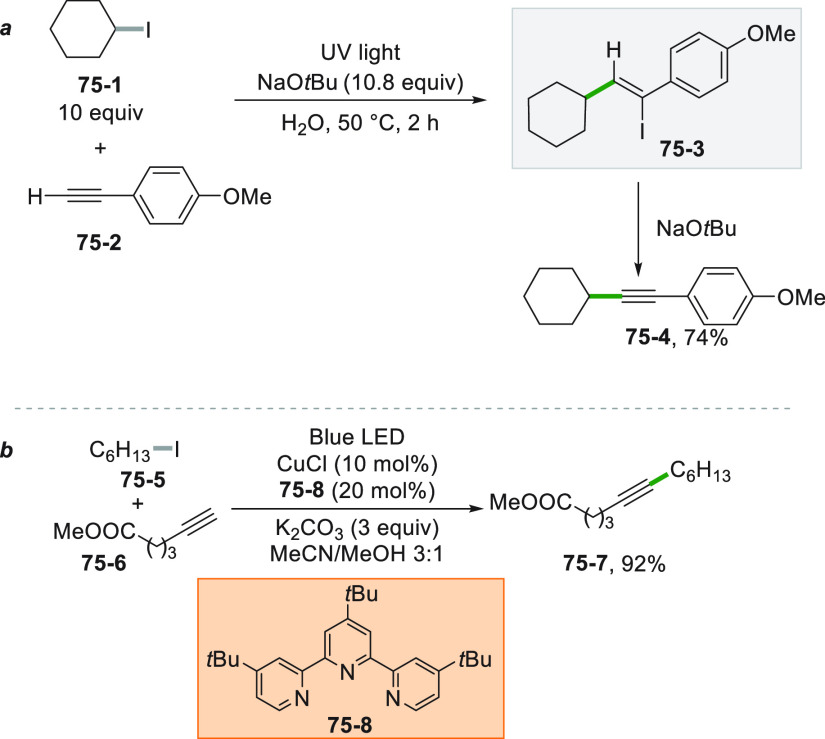
Alkylation of Terminal Alkynes

The versatility of the photocatalytic method, however, allowed to obtain functionalized alkynes starting from terminal alkynes (Scheme 75). The first approach involves the UV light induced cleavage of the C–I bond in iodide **75**–**1** (used in large excess) in basic aqueous media. Addition of the cyclohexyl radical onto alkyne **75**–**2** followed by the incorporation of the iodine atom gave vinyl iodide **75–3.** The strong basic conditions used (NaO*t*Bu) coupled with heating (up to 50 °C) favored an elimination of HI to yield the desired alkyne **75**–**4** under metal-free conditions (Scheme 75a).^280^ Visible-light (450 nm) was used in the copper-catalyzed coupling of an alkyl iodide (**75**–**5**) and again a terminal alkyne (**75**–**6**, Scheme 75b). The success of the reaction was ascribed to the use of terpyridine ligand **75**–**8** that avoided the photoinduced copper-catalyzed polymerization of the starting substrates. Probably, the reaction started by the excitation of the first formed copper acetylide that upon SET with **75**–**5** promoted the synthesis of alkyne **75**–**7** in high yields.^281^

## 3 Formation of a C(sp^3^)-Y Bond

### 3.1 C–B Bond

**Scheme 76:**
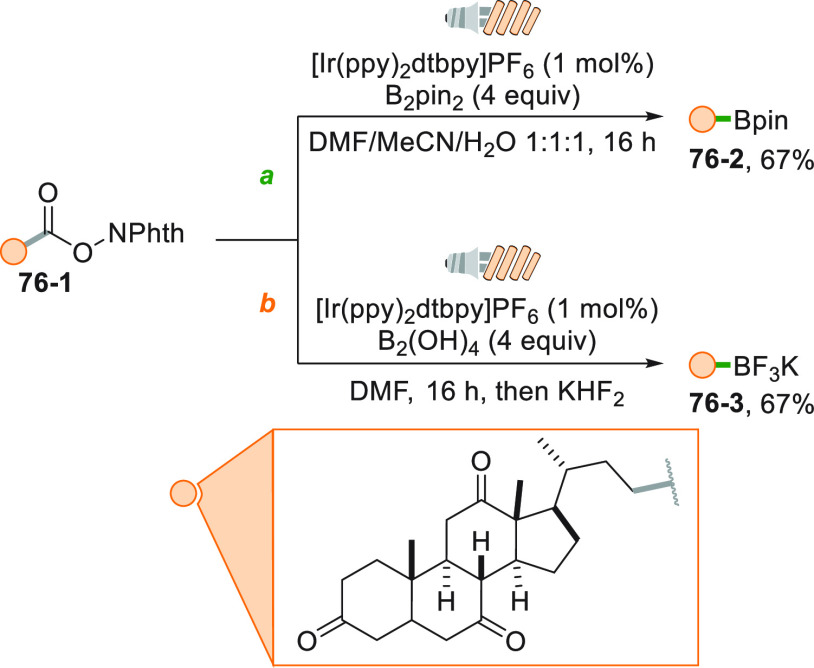
Photocatalyzed Borylation of *N*-Hydroxyphthalimides

Borylation of an alkyl derivative to access differently substituted boron containing compounds can be carried out under mild conditions, employing different photochemical approaches. Thus, the alkyl radical formed from an *N*-hydroxyphthalimide **76**–**1** (derived from dehydrocholic acid) may be trapped either by bis(pinacolato)diboron (B_2_pin_2_) to give the corresponding alkyl pinacol boronates **76**–**2** (Scheme 76, path a) or by tetrahydroxydiboron (B_2_(OH)_4_) followed by treatment with KHF_2_ to give alkyl tetrafluoroborates (Scheme 76, path b).^282^

**Scheme 77:**
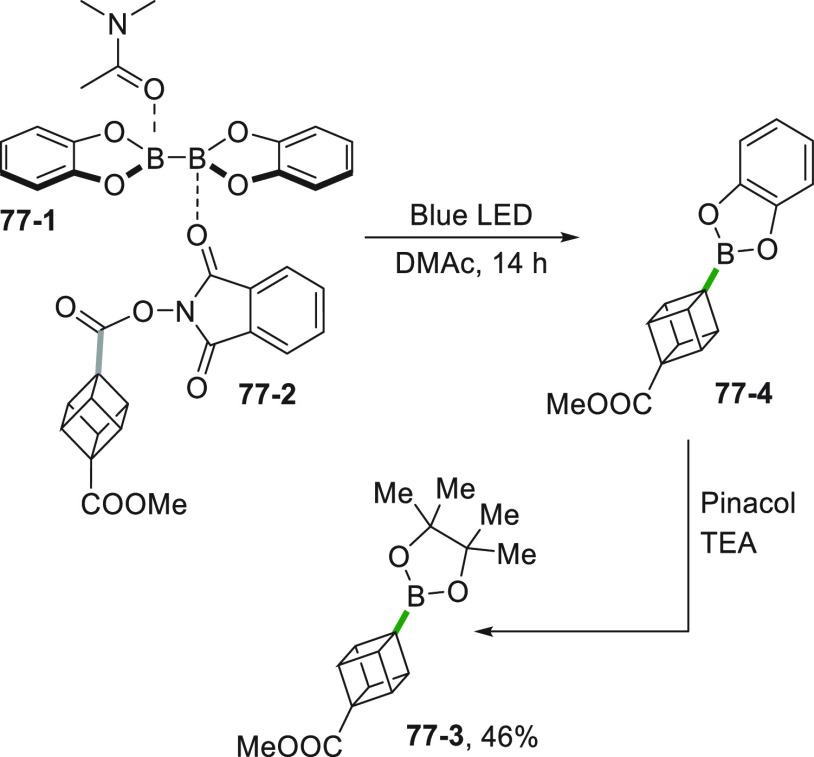
Photocatalyst-Free Borylation

A variation of the previous methodology involves the irradiation of *N*-hydroxyphthalimide esters **77**–**2** in the presence of B_2_cat_2_ (**77**–**1**) with the help of *N,N*-dimethylacetamide (DMAc) as the solvent under uncatalyzed conditions (Scheme 77). These components formed a heteroleptic ternary complex able to be excited by blue light and ultimately leading to the corresponding benzo[1,3,2]dioxaborole **77**–**4** that upon treatment with pinacol and TEA released the desired pinacol boronic ester **77**–**3**. The functionalization of a series of drugs and natural products, such as pinonic acid and fenbufen were likewise effective, underlying the broad scope and functional group tolerance of the method.^283^

Two related approaches were later developed and involve the irradiation of the ternary complex formed by differently substituted *N*-alkyl pyridinium salts, B_2_cat_2_ and DMAc. The reaction gave again pinacol boronic esters in what is considered a deaminative protocol for the borylation of aliphatic primary amines since the latter compounds were used for the synthesis of the pyridinium salts.^284−286^

**Scheme 78:**
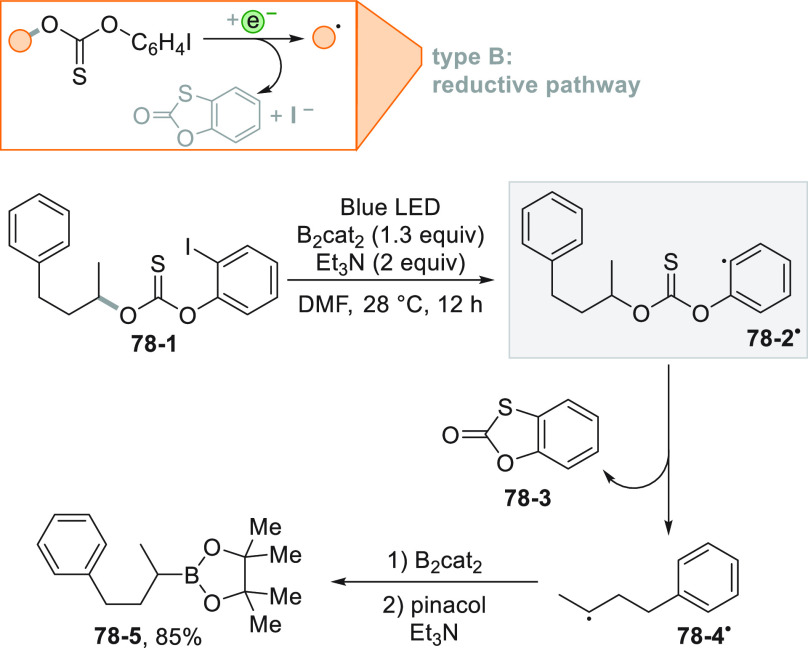
Borylation of 2-Iodophenyl Thionocarbonates

Interestingly, 2-iodophenyl thionocarbonates were later adopted as radical precursor for the preparation of boronic ester via photocatalyzed reaction with B_2_cat_2_ (Scheme 78).^95^ The strategy is based on the photoinduced reduction of compound **78**–**1** that upon iodide anion elimination formed aryl radical **78**–**2**^**•**^ that underwent a 5-*endo*-trig cyclization causing the release of benzo[*d*][1,3]oxathiol-2-one **78**–**3** and alkyl radical **78**–**4**^**•**^. Usual borylation gave boronic ester **78**–**5** in 85% yield.

### 3.2 C–N Bond

Diverse structural motifs based on the C–N bond such as hydrazine and hydrazide cores were accessed by the photochemical addition of alkyl radicals onto the N=N of azodicarboxylates. TBADT-photocatalyzed HAT was applied to synthesize hydrazines by the coupling of cycloalkyl radicals with diisopropyl azodicarboxylate (DIAD). A synthetically challenging three component reaction can be achieved in the presence of CO, allowing the synthesis of the corresponding hydrazides.^287^

**Scheme 79:**
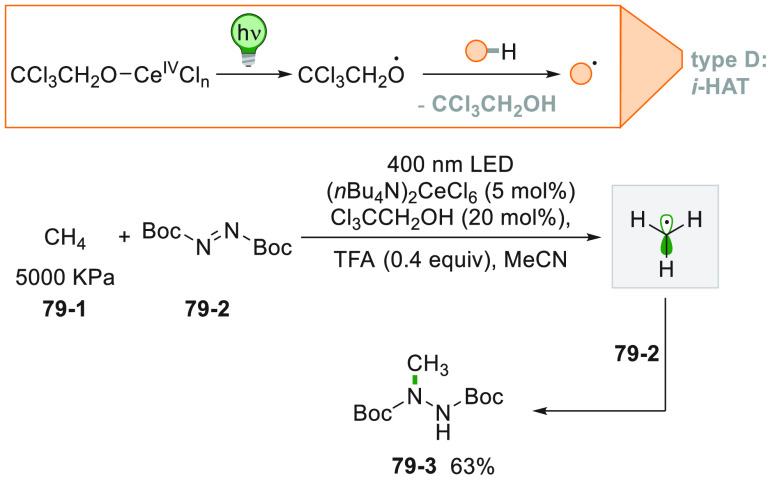
Photocatalyzed Amination of Methane

The C–H amination can be smoothly achieved even starting from light hydrocarbons, such as methane (**79**–**1**, Scheme 79), with di*tert*-butylazodicarboxylate (DBAD, **79**–**2**) in the presence of Ce^III^ salts. This inexpensive photocatalyst furnished the desired product **79**–**3** in 63% yield, with a turnover number up to 2900. The authors proposed a ligand-to-metal charge transfer excitation between the cerium salt and trichloroethanol as the source of alkoxy radicals that acted as hydrogen atom transfer agents.^288^

**Scheme 80:**
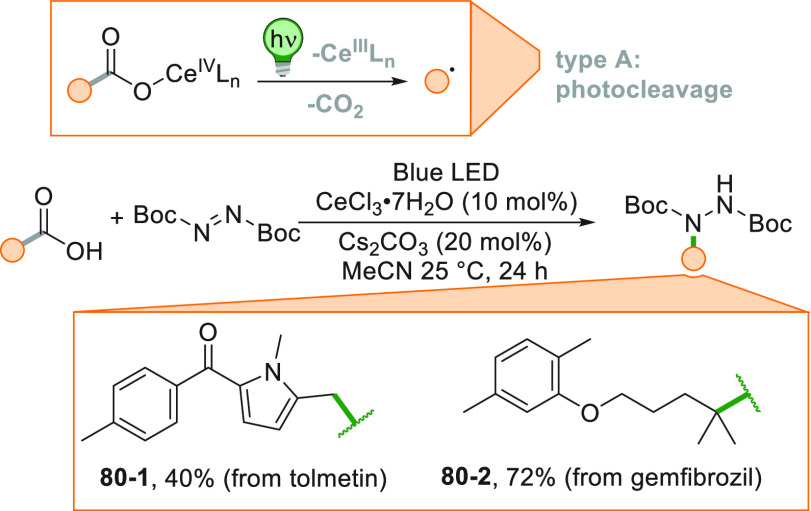
Cerium-Catalyzed Decarboxylative Amination

Aminated alkanes can be obtained by reacting aliphatic carboxylates with DIAD making use of Acr^+^Mes as a photoredox catalyst.^289^ A cerium catalyst was adopted for the generation of several alkyl radicals starting from carboxylic acids, under basic conditions, allowing for the functionalization of a broad range of substrates, including natural products such as drugs like gemfibroxil (**80**–**2**) and tolmetin (**80**–**1**, Scheme 80).^290^

**Scheme 81:**
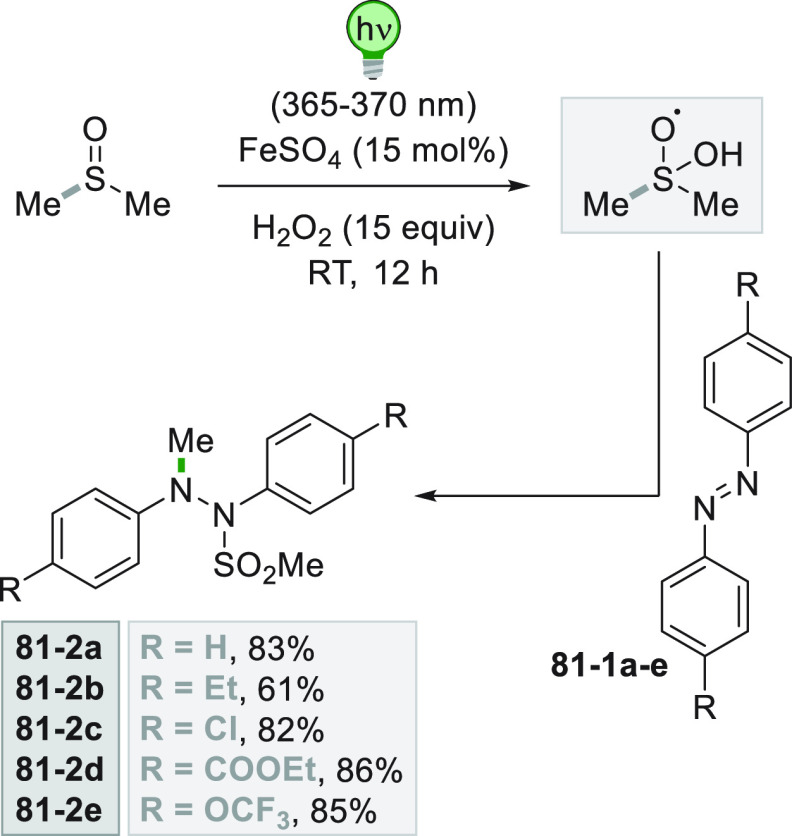
Tandem *N*-Methylation and *N*-Sulfonylation of Azobenzenes

The N=N bond of differently substituted azobenzenes (**81–1a–e**) can be functionalized on both nitrogens with a tandem *N*-methylation and *N*-sulfonylation, by cleavage of DMSO by UV irradiation of the Fenton reagent (FeSO_4_/H_2_O_2,_Scheme 81).^105^

**Scheme 82:**
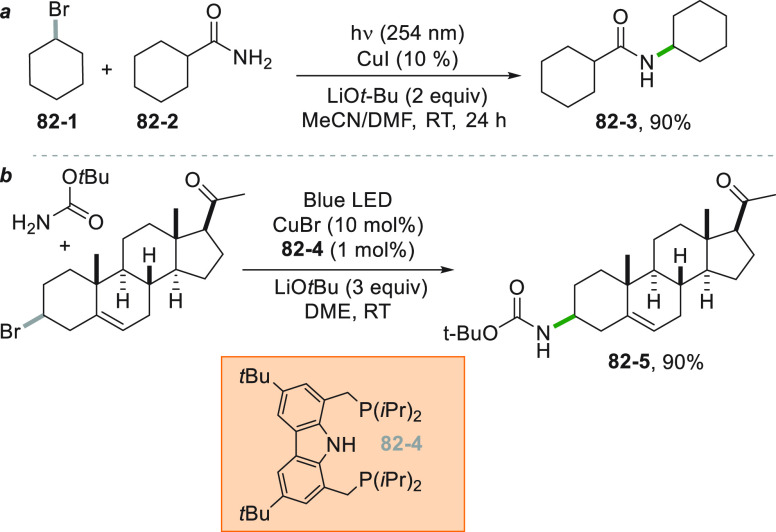
Photocatalyzed Synthesis of (a) Amides and (b) Carbamates

Synthesis of amides can be achieved recurring to copper photocatalysis. Secondary alkyl bromide **82**–**1** could be efficiently coupled with cyclohexane carboxyamide **82**–**2** in 90% yield using CuI in catalytic amounts (Scheme 82a). The authors were able to isolate the catalytic species (a copper–amidate complex), formed by the assembly of four copper ions and four amides.^291^

The same group reported the functionalization of carbamates with secondary alkyl bromides by shifting the wavelength of irradiation in the visible region by developing a tridentate carbazolide/bisphosphine ligand **82**–**4** for the copper catalyst thus able to prepare Boc-pregnenolone **82**–**5** in 90% yield (Scheme 82b).^292^ A variation of this protocol was applied to the synthesis of amines, using secondary unactivated alkyl iodides and CuI/BINOL as the catalytic system.^293^

**Scheme 83:**
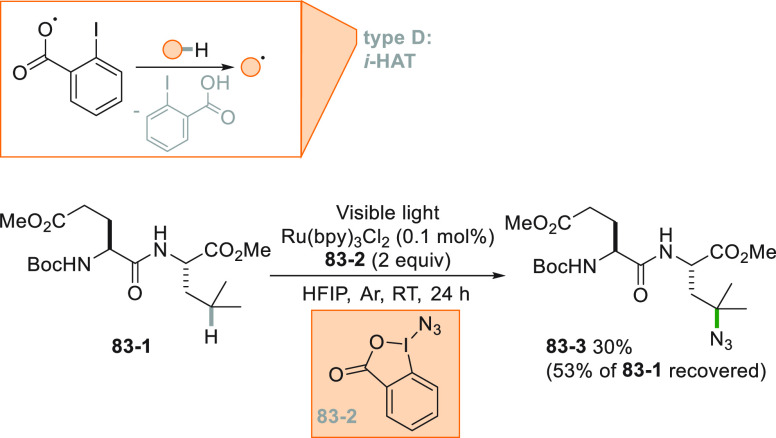
Azidation of Tertiary Aliphatic C–H Bonds

Several reagents can be used as an azide source to synthesize synthetically valuable C–N_3_ bonds. Tertiary aliphatic C–H bonds can be selectively functionalized via Zhdankin azidoiodane reagent **83**–**2**. Visible light was used to excite Ru(bpy)_3_Cl_2_ that cleaves the labile I–N_3_ bond, triggering the cascade of radical reactions that leads to the product formation (Scheme 83). The selectivity and compatibility of this reaction with different groups is underlined by the conversion of the dipeptide **83**–**1** to **83**–**3** in 30% yield.^294^ A related C–H azidation was performed by using tosyl azide as an alternative azide source with the help of 4-benzoylpyridine to promote the photocatalytic C–H cleavage in various cycloalkanes.^295^

**Scheme 84:**
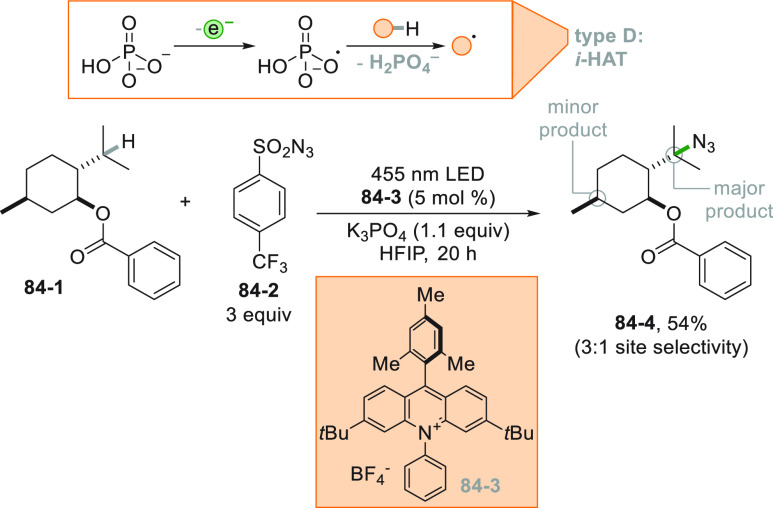
Photocatalyzed C–H to C–N_3_ Conversion

Another example of the functionalization of unactivated C–H bonds is depicted in Scheme 84 making use of tosyl azide **84**–**2**. The reaction needs the intermediacy of an oxygen radical center on a phosphate group, previously oxidized by the action of the mesityl acridinium photocatalyst **84–3.** This allows the C–H to C–N_3_ conversion in menthol benzoate **84**–**1** to give azide **84**–**4** in a satisfying yield, with a regioselectivity favoring the more electron-rich tertiary position.^296^

**Scheme 85:**
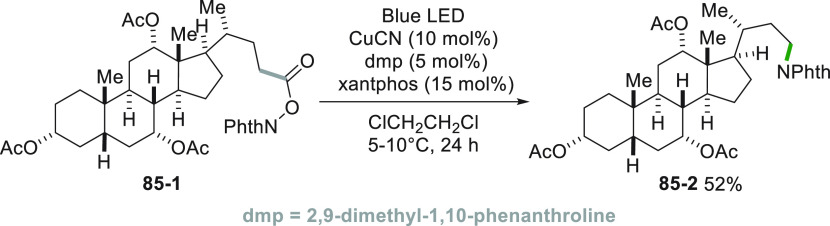
Decarboxylative C–N Coupling in Cholic Acid Triacetate

The synthesis of amines is undoubtedly more challenging to be dealt with, relying on radical chemistry. However, several strategies were developed to effectively forge this fundamental functional group. A classic reaction for the synthesis of amine is the Curtius reaction that has the drawback in handling of potentially dangerous azides. A dual copper/photoredox catalytic approach mimicked this process for the obtainment of *N*-protected amines from the *N*-hydroxyphthalimide ester of cholic acid triacetate **85**–**1** (Scheme 85, see also Scheme 8). The alkyl radical was again formed by Cu^I^-photocatalyzed reduction of **85**–**1**, but this recombine with the Cu^II^–phthalimide complex formed to release **85**–**2** (52% yield) by a formal decarboxylation process. A great variety of functional groups are compatible with this reaction including steroidal structures.^297^

**Scheme 86:**
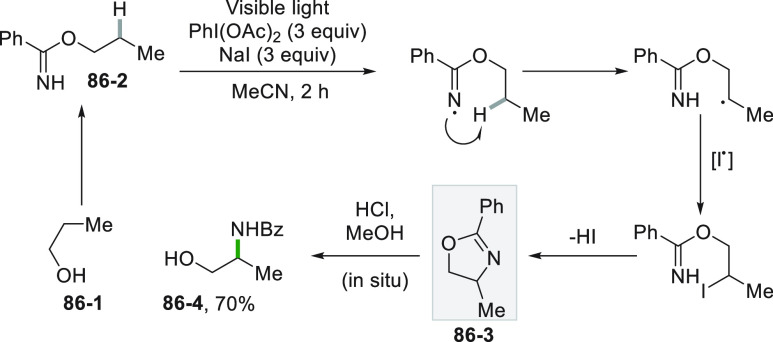
Radical Relay Chaperone Strategy Driven by the Photodecomposition of PhI(OAc)_2_

A very interesting approach to synthesize β-aminoalcohols from the unfunctionalized alcohol **86**–**1** relies on the introduction of a radical relay chaperone to direct the C–H functionalization of the β position of the OH group (Scheme 86). Imidate radicals can be accessed via the photodecomposition of PhI(OAc)_2_. A transient sp^2^*N*-centered radical is generated from **86**–**2**, which allows a 1,5-hydrogen atom transfer. A source of iodine promotes the formal transfer of an iodine radical to the β-position to the imidate, followed by cyclization to obtain **86**–**3** which can be promptly hydrolyzed to **86**–**4**. The nature of the substituents on acetimidate **86**–**2** may affect the overall yield.^298^

**Scheme 87:**
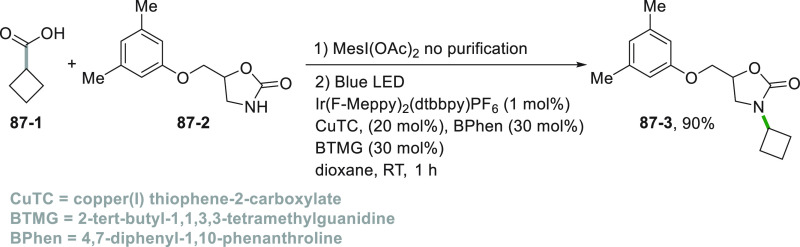
Late Stage Functionalization of Skelaxin

Direct cross-coupling between alkyl carboxylic acids and nitrogen nucleophiles can be achieved by dual copper/photoredox catalysis through iodonium activation. The scope of the transformation is broad and applicable to a diverse array of nitrogen nucleophiles such as heterocycles, amides, sulfonamides, and anilines to give the corresponding C–N coupling product in excellent yields on short time scales (5 min to 1 h). The high regioselectivity obtained in late stage functionalization of complex pharmaceuticals such as Skelaxin **87**–**2** (to give **87**–**3** in 90% yield from **87**–**1**, Scheme 87, see also Scheme 48) gave an idea of the importance of the approach.^299^

Similar strategies were explored for the synthesis of amines via C(sp^3^)–N cross-coupling combining a copper catalyst and the action of a photoredox catalyst by using anilines^300^ or benzophenone imines^301^ as nitrogen source. Hydroxylamines were instead formed under photoorganocatalytic conditions by reaction of carboxylic acids and nitrosoarenes.^302^

### 3.3 C–O Bond

**Scheme 88:**
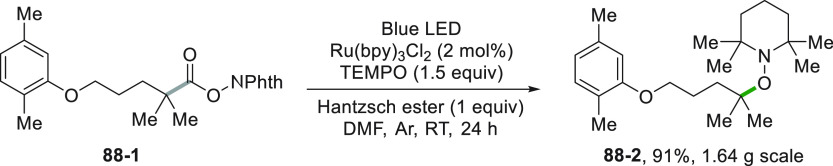
Decarboxylative Oxygenation of Phthalimide Esters

The C–O bond formation is without doubt a prerogative of polar chemistry. However, there are examples of photochemically driven reactions making use of an alkyl radical for the introduction of different oxygen-containing functional groups. In Scheme 88, the nonenolizable ester **88**–**1** is transformed into **88**–**2** via a photochemically promoted decarboxylation of the NPhth-ester (see also Scheme 8) in the presence of Hantzsch ester to yield a tertiary radical. The intermediate is promptly quenched by TEMPO, affording **88**–**2** in 91% yield, in a multigram scale reaction.^303^

**Scheme 89:**
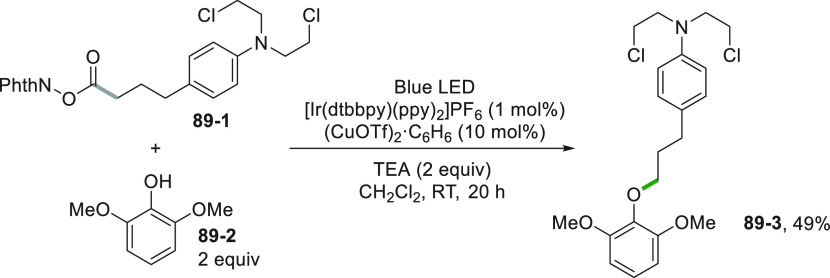
Decarboxylative C(sp^3^)-O Cross-Coupling

A similar reaction was employed to synthesize alkyl aryl ethers, given their importance in medicinal and agricultural chemistry. A tandem photoredox and copper catalysis approach allows the decarboxylative coupling of alkyl *N*-hydroxyphthalimide esters (NHPI) with phenols (**89**–**2**Scheme 89). Various NHPI esters of different drugs and natural products easily underwent a late-stage decarboxylative etherification. As an example, the chlorambucil derivative **89**–**1** was converted into the corresponding 2-MeO phenyl ether **89**–**3** in 49% yield.^304^

**Scheme 90:**
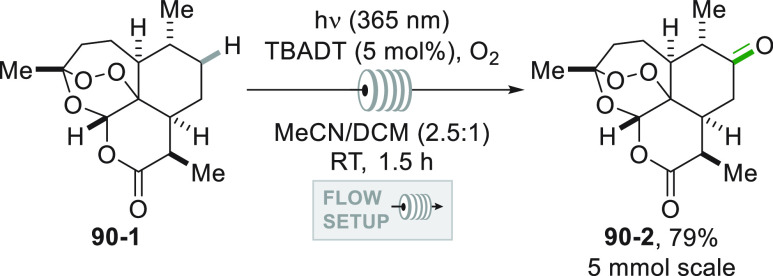
Late Stage Regioselective Carbonylation of Artemisinin

Following a similar strategy, carboxylates are converted into alcohols via a photocatalytic decarboxylative hydroxylation mediated by the mesityl acridinium salt. In this case, molecular oxygen is used as the oxidant, to promote the formation of the desired C–O bond. Since the reactions gave mainly a mixture of ketones and hydroperoxides, reduction *in situ* by sodium borohydride allowed the synthesis of alcohols in good yields.^305^ A decarboxylative hydroxylation may be carried out with the intermediacy of Barton esters that upon irradiation in oxygen-saturated toluene followed by treatment with P(OEt)_3_ afforded an alcohol intermediate for the total synthesis of Crotophorbolone.^306^ The more challenging oxidation of unactivated alkanes to alcohols or ketones can be achieved through a photoelectrochemical approach, as testified by the C–H bond activation of cyclohexane to prepare a mixture of cyclohexanone and cyclohexanol (the so-called KA oil) with high partial oxidation selectivity (99%) and high current utilization ratio (76%). The highest current ratio was obtained illuminating the solution with 365 nm wavelength.^307^ Decatungstate photocatalysis was efficiently applied to oxidize activated and unactivated C–H bonds. Taking advantage of a microflow reactor setup, a late stage regioselective CH_2_/C=O conversion in several natural compounds, such as artemisinin **90**–**1** to form artemisitone-9 **90**–**2** was readily pursued even in a 5 mmol scale (Scheme 90).^308^

### 3.4 C-Halogen Bond

Halogenation of alkanes through a radical reaction under UV irradiation is one of the core pathways to chemically activate a paraffin. Industrially, chlorine gas is used to functionalize methane. A major drawback of the classical chain reaction using either Cl_2_ or Br_2_ under direct irradiation is the formation of di or polyhalogenated products. The application of microflow technology in combination with visible light irradiation (with an absorption maximum in the near UV at ca. 350 nm) allowed the monobromination of different alkanes with molecular bromine. High selectivity for the monobrominated compound and excellent overall yields (between 60 and 99%) could be achieved for secondary and tertiary alkanes, along with primary benzylic positions.^309^

**Scheme 91:**
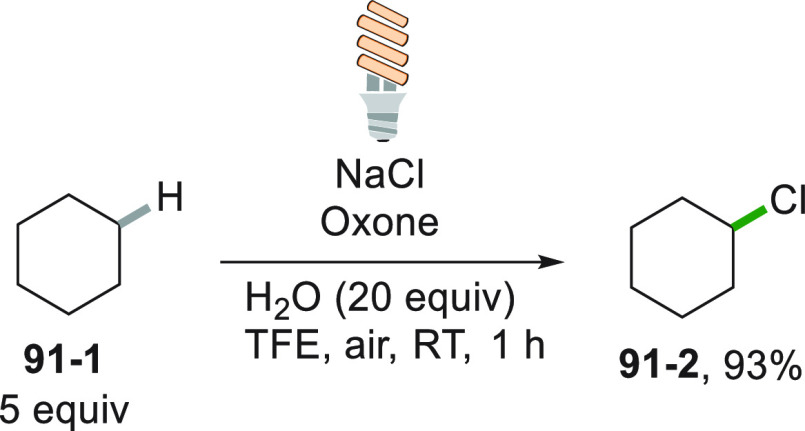
Photoinduced Monochlorination of Cyclohexane

Chlorination with molecular chlorine, on the other hand, suffers from the low yields of the reaction, typically around 50%, from the high concentrations of HCl generated in the process and from the toxicity of the chlorine gas itself. However, when Cl_2_ was generated by mixing NaClO with HCl and the chlorination took place under flow conditions, efficient C–H to C–Cl conversion resulted.^310,311^ A photochemical alternative using NaCl as chlorine source was developed.^312^ In the reaction, Cl_2_ was formed *in situ* by oxidation of the chloride anion with oxone. The monochlorination of cyclohexane **91**–**1** to give **91**–**2** could be obtained in 93% isolated yield thus overcoming the limitation of the classical chlorination process with chlorine gas (Scheme 91, see also Scheme 15).

**Scheme 92:**
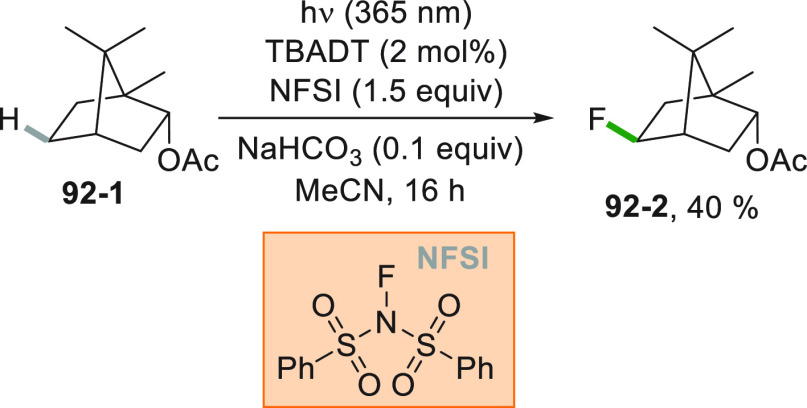
TBADT-Catalyzed Fluorination of Alkanes

Fluorination is essential to modern medicinal chemistry, both as a viable way to insert radiotracers or to deactivate specific degradation pathways in drugs. Photochemistry is a reliable tool to achieve the fluorination of C–H bonds, following different strategies. Excited TBADT may formed a radical intermediate (from unactivated alkanes) that abstracts the fluorine atom from the labile N–F bond of the fluorinating agent *N*-fluorobenzenesulfonimide (NFSI). An *N*-centered radical resulted which closes the radical cycle oxidizing the reduced photocatalyst. Acetate **92**–**1** was fluorinated in 40% yield following this procedure to yield **92**–**2** (Scheme 92). The reaction applied to sclareolide, however, was not selective and gave a mixture of fluorinated regioisomers (68% overall yield).^313^

Following a similar reaction scheme, uranyl acetate was employed in combination with NFSI to promote the fluorination of secondary alkanes but poorly on the benzylic positions. Indeed, in the absence of an aromatic scaffold, the excited U=O abstract a hydrogen atom through HAT, while the presence of an aromatic ring deactivated the excited state of the catalyst via exciplex formation preventing the fluorination to occur.^314^ Acetophenone in its excited state promoted the hydrogen abstraction of secondary alkanes, with the advantage that a common CFL housebulb can be used to promote an efficient conversion, using Selectfluor as the fluoride source.^315^ In this case, the authors irradiated the tail of the n-π* absorption band of the ketone which can be found in the visible region due to the high concentration of the photocatalyst present in solution. *N*-Alkyl phthalimides having an alkyl chain linked to the nitrogen was fluorinated by using Selectfluor under photocatalyst-free conditions. An exciplex was supposed to be formed between the reagents and it was proposed that the C–F bond formation took place concomitantly with hydrogen atom abstraction with the nitrogen radical of the fluorinating agent.^316^

**Scheme 93:**
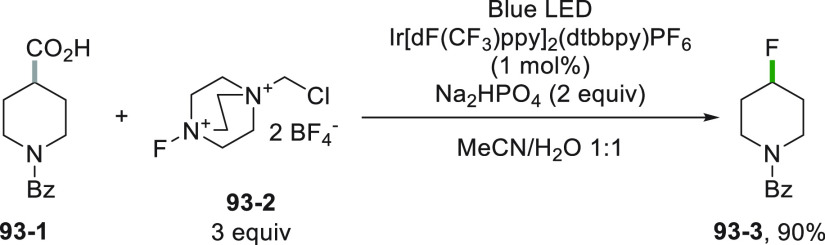
Regioselective Fluorination of Carboxylates

A considerable regioselectivity in the fluorination reaction can be achieved using carboxylates as alkyl radical precursors and again Selectfluor as a fluorinating reagent. The reaction is possibly initiated by reduction of Selectfluor **93**–**2** by means of Ir[dF(CF_3_)ppy]_2_(dtbbpy)PF_6_. Fluorination of different carboxylic acids can be achieved in a very high yields (between 70 and 99%), and **93**–**1** was readily converted into **93**–**3** in 90% yield (Scheme 93).^317^ In case of unactivated primary substrates, a prolonged irradiation (12–15 h) was mandatory to achieve a high conversion of the substrate.

**Scheme 94:**
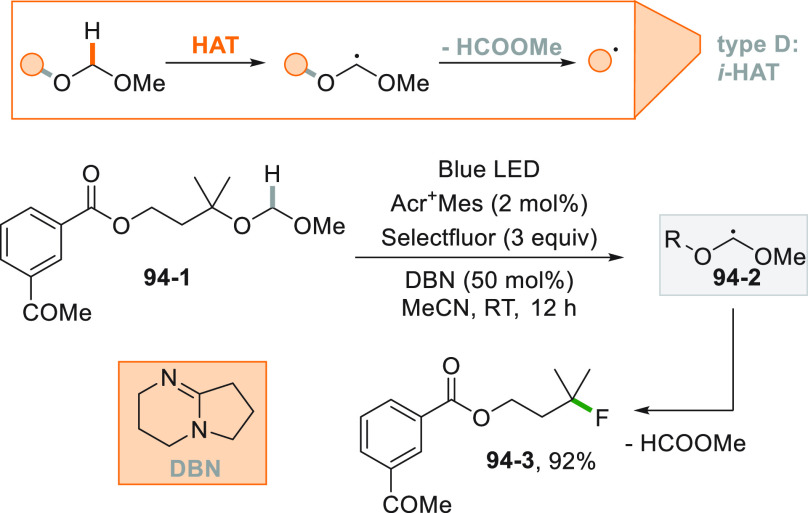
DBN-Mediated HAT in C–F Bond Formation

An interesting case is the fluorination of compounds having the MOM group to direct the halogenation event. In this case, the PC oxidized an imidine base (DBN) that acted as hydrogen atom abstractor of the dioxolanyl group in compound **94**–**1** (Scheme 94). The resulting α,α-dioxy radical **94**–**2** released an alkyl radical (upon formiate loss) that was fluorinated by Selectfluor. This metal-free approach again used visible light and is particularly successful when applied to tertiary alkyl ethers to give sterically hindered alkyl fluorides (e.g., **94**–**3**).^318^

**Scheme 95:**
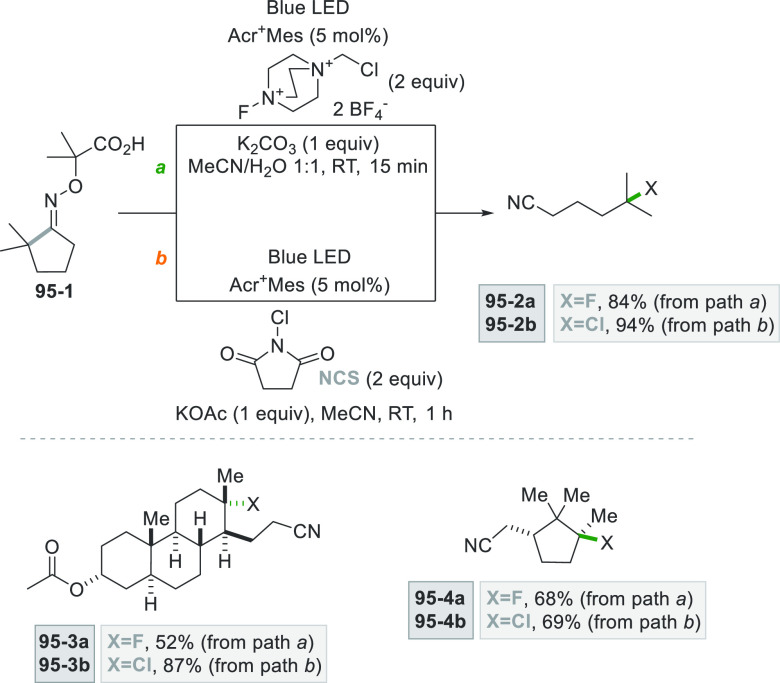
Ring-Opening Halogenation of Oximes

Interestingly, fluorination of carboxylates with Selectfluor was also reported to occur under heterogeneous photocatalytic conditions, using titania as photocatalyst to promote the oxidation of the carboxylate anion.^319^ Fluorination and chlorination of nitriles and ketones could be obtained starting from oximes, using Selectfluor and NCS as halogen sources, respectively. With this methodology, γ-functionalization of ketones and a complex photoinduced ring-opening/halogenation of oximes via the intermediacy of an iminyl radical was pursued. The C=N moiety of the reagent (e.g., **95**–**1**) was preserved in the products (**95–2a,b**) in its oxidized nitrile form (Scheme 95). Several natural products could be functionalized following this methodology, such as androsterone (**95–3a,b**) and camphor (**95–4a,b**) derivatives (Scheme 95, see also Scheme 20).^320^

Alcohols were converted into their corresponding pyruvates that upon irradiation in the presence of an Ir^III^ photocatalyst with blue LEDs released an alkyl radical prone to be chlorinated by 2,2,2-trichloroacetate as the chlorine atom source. A series of secondary and tertiary chlorides could be obtained in good to excellent yields.^321^

**Scheme 96:**
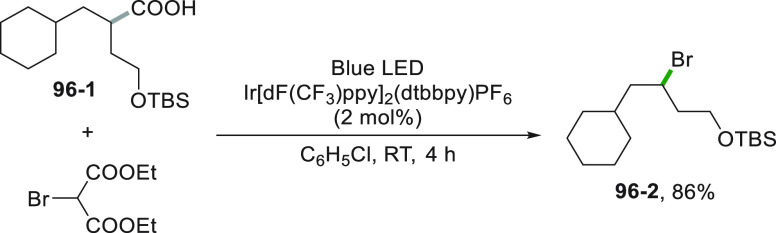
Bromomalonate as Brominating Agent

An Ir-based photocatalyst was used to promote bromination of carboxylic acid (**96**–**1**) with bromomalonate as brominating agent (Scheme 96).^322^ The acids used in this work were likewise converted into the corresponding alkyl chlorides and iodides in the presence of the corresponding *N*-halosuccinimides.^322^

**Scheme 97:**
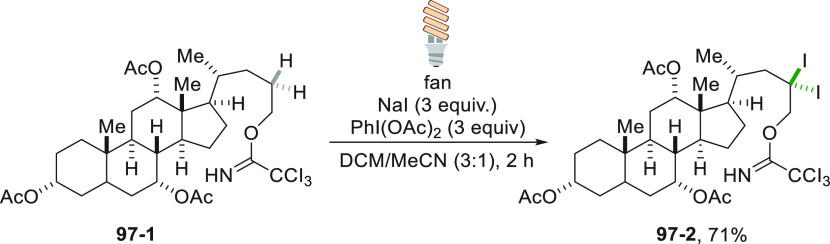
Gem di-Iodination of a Cholic Acid Derivative

A radical relay strategy was employed to synthesize gem-diiodides through successive intramolecular 1,5-HAT processes and iodine trapping. Indeed, the excitation with visible light of an N–I imidate group, formed *in situ* from the reaction of a trichloroacetimidate with PhI(OAc)_2_ as an iodine source, allowed the synthesis of a small library of gem di-I compounds in good yields. As an example, the cholic acid derivative **97**–**1** has been converted to its corresponding di-iodo derivative **97**–**2** in 71% yield (Scheme 97). Moreover, the authors could also achieve a dibromination using NaBr and TBABr and visible light, while only monochlorination is reported when NaCl, TBACl, and UV light were adopted.^323^

### 3.5 C–S or C–Se Bonds

Alkyl radicals were sparsely used for the unusual introduction of a SCF_2_X (X = F, H) or an SAr moiety in an organic compound. The introduction of a SCF_2_X group has recently sparked attention due to the remarkable hydrogen donor nature of the group when X = H, making it the lipophilic surrogate for OH or NH groups.^324^ On the other hand, the trifluoromethylthio group increases the metabolic stability and the lipophilicity of drugs.

**Scheme 98:**
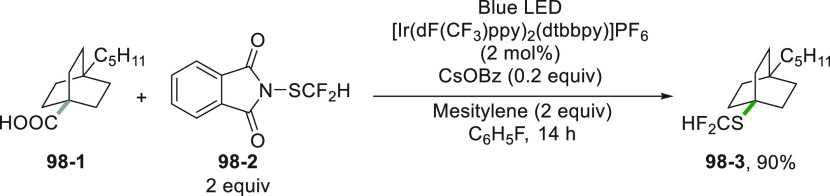
Difluorothiomethylation of Carboxylic Acids

One strategy for the introduction of a SCF_2_X group is the photocatalyzed (by Ir^III^ PC) oxidation of alkyl carboxylates via visible light irradiation in the presence of PhthN-SCF_2_H (**98**–**2**) as the sulfur donor. Indeed, **98**–**1** was converted into **98**–**3** in high yields (Scheme 98). The reaction was sustained by the stability of the imidyl radical liberated in the process, that was able to promote a chain reaction oxidizing a further carboxylate group. Indeed, the quantum yield for the reaction was found to be 1.7.^325^ Bis-methyltiolation was observed in different cases, possibly due to HAT triggered by an intermediate of the reaction, presumably PhthN^•^ and following transfer of SCF_2_X from the reactant. To avoid the formation of byproducts either mesitylene or 3-(methyl) toluate were added as sacrificial hydrogen donors.

**Scheme 99:**
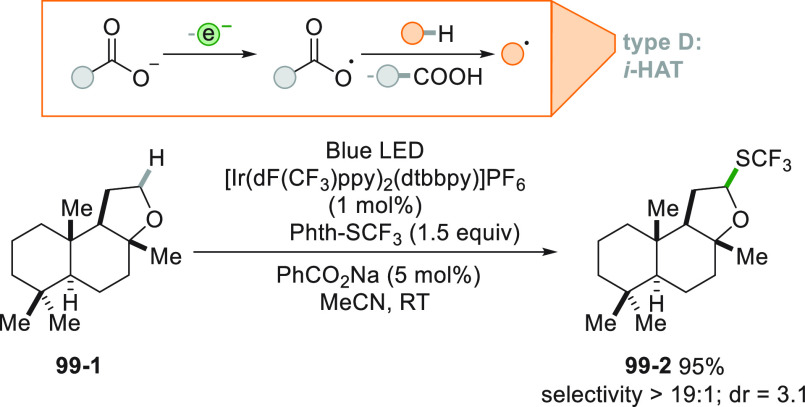
Trifluoromethylthiolation of Ambroxide

An interesting follow-up for this methodology from the same group made use of the hydrogen atom transfer process previously reported as detrimental for the reaction yield. In fact, when using an aryl carboxylate instead of an aliphatic one, the carboxyl radical that is formed upon electron transfer with the excited Ir catalyst is now stable enough to act as a hydrogen abstractor, selectively targeting secondary or tertiary H in alkyl chains. Also, in this case, PhthN-SCF_2_X acted as the sulfur source. The conversion of ambroxide **99**–**1** to its trifluorothiomethyl derivative **99**–**2** proceeded with 95% yield (Scheme 99).^326^

**Scheme 100:**
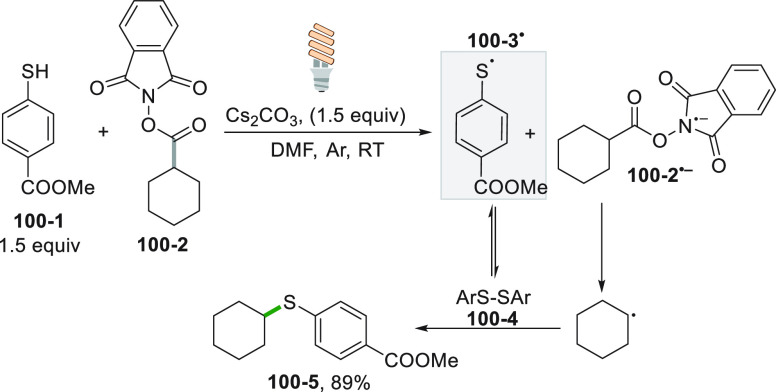
Arylthiation of *N*-Acyloxyphthalimides

A photocatalyst-free decarboxylative arylthiation took place by mixing an *N*-acyloxyphthalimide (e.g., **100**–**2**) in the presence of an aryl thiol (**100**–**1**) under basic conditions (by Cs_2_CO_3_) upon visible light irradiation. In this case, a SET between **100**–**1** and **100**–**2** caused the formation of **100**–**2**^**•–**^ along with thiyl radical **100**–**3**^**•**^ (that easily dimerized to disulfide **100**–**4**). Trapping of the resulting cyclohexyl radical (by loss of PhthN^**–**^ from **100**–**2**^**•–**^) with **100**–**4** afforded alkylaryl sulfide **100**–**5** in 89% yield (Scheme 100).^327^

**Scheme 101:**
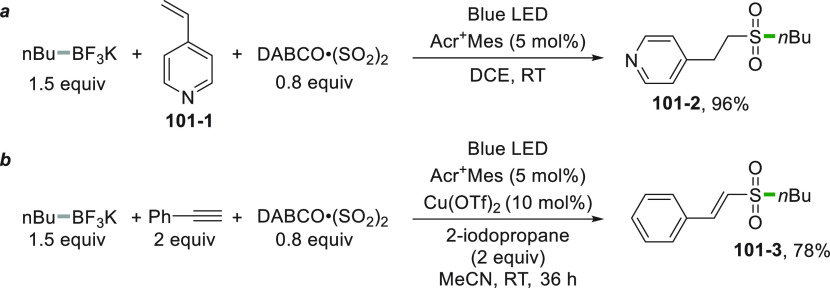
Sulfonylation of (a) Styrenes and (b) Alkynes

The most widely used reaction for the C–S bond synthesis requires the incorporation of sulfur dioxide by using DABSO (DABCO(SO_2_)_2_) as its surrogate as depicted in Scheme 101.^328^ Thus, excited mesityl acridinium salts promoted the oxidation of an alkyl-BF_3_K salt that generated a nucleophilic radical able to react with DABSO. The sulfonyl radical intermediate formed has been employed in a three-component reaction with electron poor olefins (e.g., a vinyl piridine **101**–**1**, Scheme 101a)^329^ or an alkyne (phenyl acetylene, Scheme 101b),^330^ affording alkyl sulfone (**101**–**2**) or (*E*)-vinyl sulfone (**101**–**3**), respectively.

Alternatively, alkyl iodides can be used to react with olefins decorated with EWGs and DABSO to generate a broad range of alkyl sulfones.^331^ A very similar strategy was implemented by the same authors using differently substituted Hantzsch esters as alkyl radical precursors, upon irradiation in the presence of Eosin Y.^332^ In the latter case, the sulfonyl radical added onto vinyl azides and, after releasing of molecular nitrogen, an imidyl radical resulted which reacted with the reduced photocatalyst, forming an anion that is easily protonated. After a tautomeric equilibrium, (*Z*)-2-(alkylsulfonyl)-1-arylethen-1-amines were formed, with good regioselectivity and complete control over the configuration of the double bond.^332^

Cyclobutanone oximes can be reduced via photocatalytic means in the presence of Ir(dtbbpy)(ppy)_2_PF_6_ to form γ-cyanoalkyl radicals after radical fragmentation. In this process, a vinyl sulfone was used having the dual role of radical acceptors and SO_2_ source, allowing the synthesis of β-ketosulfones or allylsulfones through a radical transfer mechanism.^333^

### 3.6 C–H Bond

**Scheme 102:**
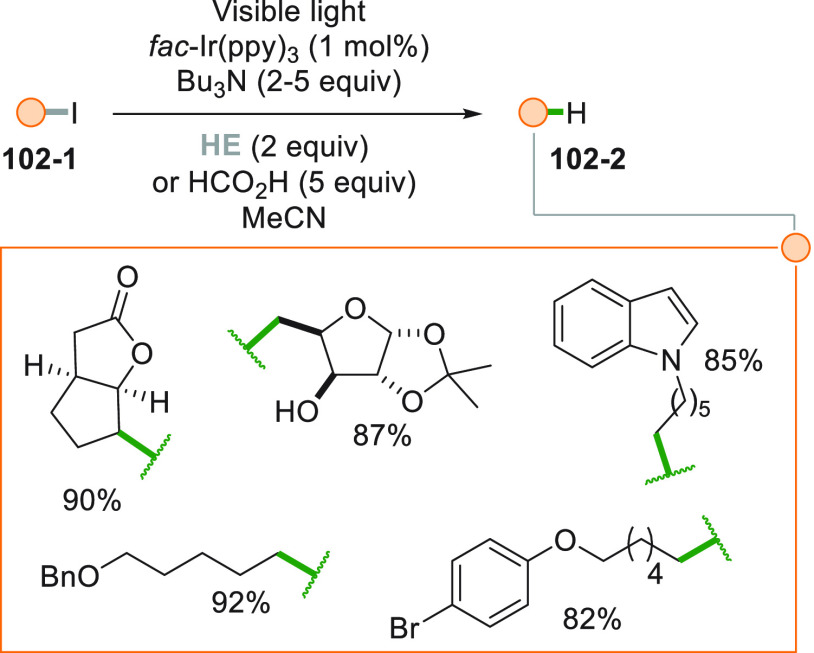
Photocatalyzed Reduction of Alkyl Iodides

Classical radical reductive dehalogenation is one of the most successful reactions based on tin chemistry.^21^ Photocatalysis and photochemistry propose a milder and more environmentally friendly alternative to this process, via different strategies. As an example, *fac*-Ir(ppy)_3_ was used to convert alkyl iodides **102**–**1** into their corresponding alkyl radicals using Hantzsch ester or HCO_2_H as the hydrogen atom source for the HAT process that drives the reaction to the formation of **102**–**2** (Scheme 102). The authors optimized their procedure by using tributylamine as the sacrificial electron donor to reduce the oxidized form of the catalyst and restore the catalytic cycle.^334^ A variation of this protocol using *p*-toluenethiol, DIPEA, and *fac*-Ir(mppy)_3_ was used to synthesize D-albucidin.^335^

Other catalytic systems were proved to be competent in the reduction of halides. In particular, unactivated aryl and alkyl bromides could be reduced using [Ir(ppy)_2_(dtbbpy)]PF_6_ in combination with TTMSS as a reducing agent. The mild conditions typical of the reaction were critical to obtain both the mono and the bis reduction of a gem-dibromocyclopropane in a selective fashion.^336^

Alkyl iodides and bromides were reduced under metal-free conditions via irradiation of 4-carbazolyl-3-(trifluoromethyl)-benzoic acid as the photocatalyst and 1,4 cyclohexadiene as sacrificial hydrogen donor.^337^ The reduction of C–X bonds to C–H bonds can take place under photocatalyst-free conditions by PET reactions between the halide and an amine as sacrificial reductant. In this way, adamantane was obtained in 95% yield by photochemical reduction of 1-bromoadamantane.^338^ Borohydride-mediated radical photoreduction of alkyl halides (iodides, bromides, and chlorides) is another valuable tool for the formation of a C–H bond.^339^

**Scheme 103:**
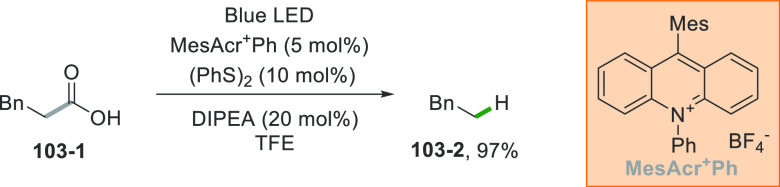
Hydrodecarboxylation of Carboxylic Acids

The C–H bond formation could be achieved via a hydrodecarboxylation of carboxylic acids. In fact, carboxylic acid **103**–**1** could be reduced in 97% yield to **103**–**2** by generating the corresponding carboxyl radical through excitation of an acridinium photocatalyst with 450 nm LEDs, in the presence of 10% mol of (PhS)_2_ (Scheme 103). The authors achieved good yields in the decarboxylation of different carboxylic acids. Most notably they succeeded in the double reduction of doubly substituted malonic acids, although with the necessity of longer irradiation times and higher catalyst loading to compensate for the increased amount of substrate to be reduced.^340^

**Scheme 104:**
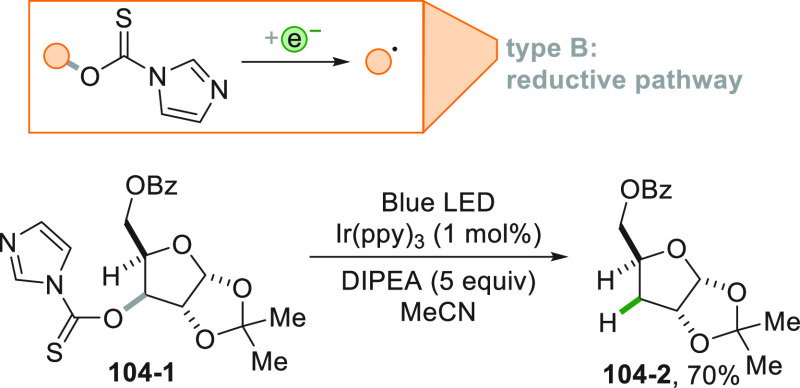
Reduction of a Xylofuranose Derivative

The challenging reduction of alcohols to the corresponding alkane can take place via functionalization of the OH group to form an *O*-thiocarbamate. This compound is the substrate of a photocatalyzed Barton-McCombie deoxygenation in combination with Ir(ppy)_3_ and DIPEA under an oxidative quenching. Accordingly, the xylofuranose derivative **104**–**1** was cleanly reduced to **104**–**2** in 70% yield by maintaining the benzoyl group in position 5 (Scheme 104).^93^ The reaction was studied mostly on secondary alcohol derivatives being another interesting alternative to the usual tin-mediated reaction.^22^

An alternative pathway to reduce the hydroxy function required a more sophisticated functionalization of the OH group making use of two consecutive photochemical reactions. Conducting the reaction in CBr_4_ under UVA irradiation, the hydroxy groups of a series of primary alcohols were converted into their bromides and then subjected to a one-pot photoreduction mediated by the dimeric gold complex [Au_2_(dppm)_2_]Cl_2_ in the presence of DIPEA.^341^

## 4 Formation of a Ring

### 4.1 Three/Four-Membered Rings

**Scheme 105:**
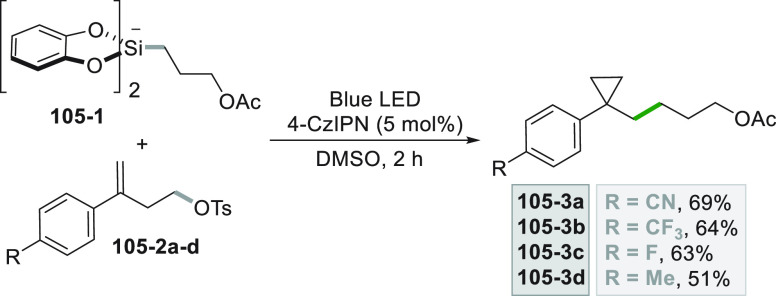
Synthesis of 1,1-Disubstituted Cyclopropanes

In this last section, selected examples will be given when a photogenerated alkyl radical is used for the construction of a ring. Scheme 105 shows one example of formation of a three-membered ring. 1,1-Disubstituted cyclopropanes **105–3a–d** were obtained through the addition of an alkyl radical (from silicate **105**–**1**) onto homoallylic tosylates **105–2a–d**. The trick here is a radical/polar crossover process where the reduction of the benzyl radical adducts to benzyl anions (by SET with the reduced form of the photoorganocatalyst 4-CzIPN) followed by intramolecular substitution gave the three-membered ring (Scheme 105).^342^ The versatility of the method was demonstrated by using alkyl trifluoroborates or 4-alkyldihydropyridines as radical precursors and a good tolerance of various functional groups.

A related approach was adopted for the construction of cyclobutanes.^102^ Here, the alkyl radical was formed by easily oxidizable electron-rich alkyl arylboronate complexes and added to an iodide-tethered alkene such as methyl 5-iodo-2-methylenepentanoate. However, changing the length of the chain in the haloalkyl alkenes led to the synthesis of three-, five-, six-, and seven-membered rings.^102^

### 4.2 Five-Membered Rings

**Scheme 106:**
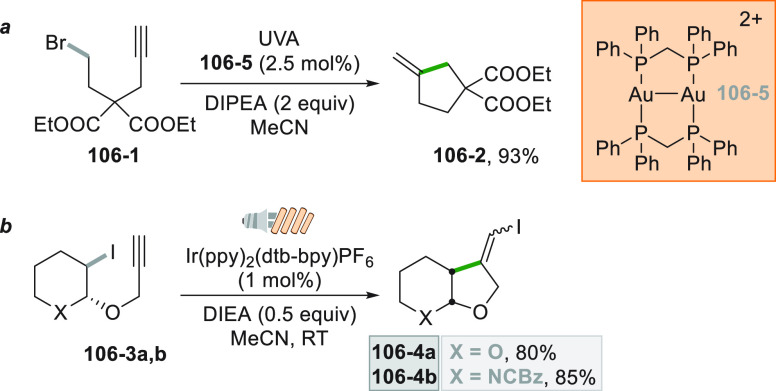
Metal-Photocatalyzed Synthesis of Cyclopentanes from Alkyl Halides

Five-membered ring is one of the privileged structures accessible via photogenerated alkyl radicals. A common approach is the cyclization onto an alkyne to form an exocyclic double bond as exemplified in Scheme 106. In most cases, an alkyl halide is reduced by an excited photocatalyst and the resulting radical cyclizes in a *5-exo dig* fashion to form the desired alkene. When using a dimeric gold complex **106**–**5** the reaction of alkyl bromide **106**–**1** generates diester **106**–**2** in 93% yield (Scheme 106a).^343^ Cyclopentanes were likewise formed starting from an unactivated alkyl iodide that underwent an intramolecular radical closure by using a strong reductant in the excited state (Ir(ppy)_2_(dtb-bpy)PF_6_). The iodine atom, however, was incorporated in the final product forming an alkenyl iodide.^344^ The same metal-based photocatalyst was effective to induce a visible light-promoted preparation of five-membered heterocycles (Scheme 106b). The cyclization step was applied on a Ueno–Stork reaction starting from 2-iodoethyl propargyl ethers (e.g., **106–3a,b**) to construct a tetrahydrofuran ring (in **106–4a,b**).^345^ The examples described in Scheme 106 required an amine as a sacrificial donor. However, amines can be used as efficient reducing agents by a PET reaction with excited alkynyl halides. The resulting photocyclization may then be carried out under metal-free conditions and in a flow photomicroreactor providing the preparation of five-membered rings in a 4 g scale.^346^

The dehalogenation/cyclization strategy was explored even under heterogeneous conditions by using platinum nanoparticles on titania (PtNP@TiO_2_) as the photoredox catalyst. The pyrrolidine scaffold was then obtained by reaction of *N*-(2-iodoethyl)-4-methyl-*N*-(prop-2-yn-1-yl)benzenesulfonamide under irradiation (DIPEA as sacrificial donor).^347^

**Scheme 107:**
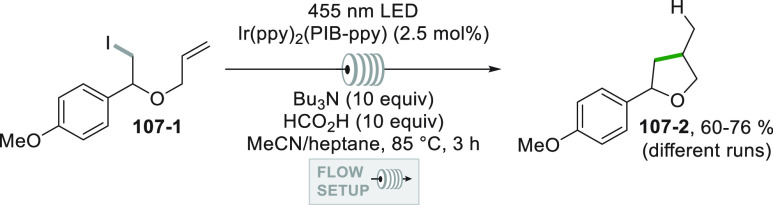
Photocatalyzed Synthesis of Tetrahydrofurans in Flow

As an alternative, a biphasic system may be adopted (Scheme 107). In fact, a polyisobutylene-tagged *fac*-Ir(ppy)_3_ complex (Ir(ppy)_2_(PIB-ppy)) soluble in heptane was prepared. The substrate **107**–**1** along with the reagents were soluble in a MeCN phase. However, heating at 85 °C allowed the two phases to mix. Preparation of tetrahydrofuran derivative **107**–**2** was then accomplished in continuous flow in a satisfying yield with an automatic recovery and reuse of the catalyst (Scheme 107).^348^

**Scheme 108:**
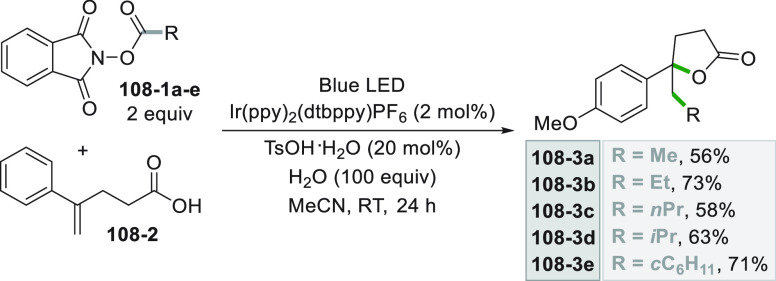
Photocatalyzed Lactonization of Alkenoic Acids

Alkyl *N*-hydroxyphthalimide esters were used as alkylation reagents in the functionalization of alkenoic acid **108**–**2** (Scheme 108). The alkyl radical added onto the double bond, and the resulting benzyl radical was oxidized to a benzyl cation readily trapped by water and cyclization of the resulting hydroxy acid gave alkyl-substituted lactones **108–3a–e** in moderate yields.^349^

Alkyl *N*-hydroxyphthalimide esters were exploited for the photocatalyzed (by a Ru^II^ complex) alkylation of *N*-arylacrylamides that caused the cyclization of the adduct radical onto the phenyl ring to afford 3,3-dialkyl substituted oxindoles.^350^ Moreover, the same radical precursors have been used for the derivatization of alkynylphosphine oxides under metal- and oxidant-free conditions to form benzo[*b*]phospholes in very good yields.^351^

**Scheme 109:**
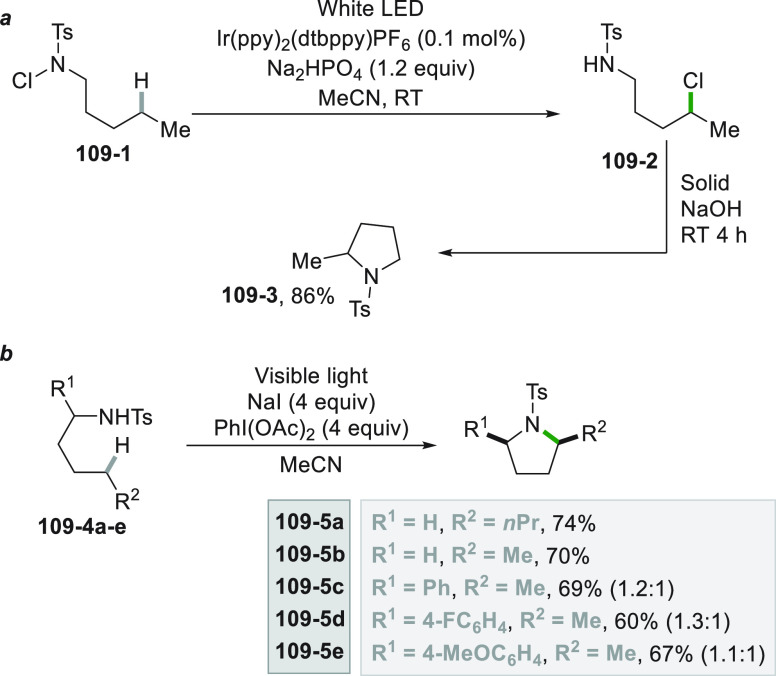
Photochemically Induced Synthesis of Pyrrolidines

A five-membered ring may be accessed via late-stage C(sp^3^)-H functionalization in *N*-chlorosulfonamides **109**–**1** (Scheme 109a). The Ir^III^-photocatalyzed reduction of **109**–**1** induced the elimination of the chloride anion along the formation of a *N*-centered radical prone to abstract a hydrogen atom from a remote position to afford an alkyl radical. Oxidation of this radical to the cation followed by incorporation of the chloride anion gave the corresponding chloride **109**–**2** that upon treatment with solid NaOH formed pyrrolidine **109**–**3** by an intramolecular nucleophilic substitution.^352^ The mildness of the process allowed the application onto biologically important (−)-cis-myrtanylamine and (+)-dehydroabietylamine derivatives.

A related intramolecular 1,5-HAT transfer was induced in *N*-tosyl amides **109–4a–e**. In this case, the haloamide is formed *in situ* by iodination by reaction of **109–4a-e** with iodine (obtained by oxidation of iodide anion by PhI(OAc)_2_). The excess of iodine allowed for tuning the amount of iodine released in solution by forming the triiodide anion. Then, visible light irradiation of the mixture induced the cyclization to give *N*-tosylpyrrolidines **109–5a–e** (Scheme 109b).^353^

Even primary nonactivated sp^3^-hybridized positions were functionalized again by a remote intramolecular radical 1,5-hydrogen abstraction in γ-bromoamides to produce several γ-lactones in a one-pot fashion.^354^ Trifluoroethyl amides were found useful as the directing group increasing the efficiency of the hydrogen abstraction process.

**Scheme 110:**
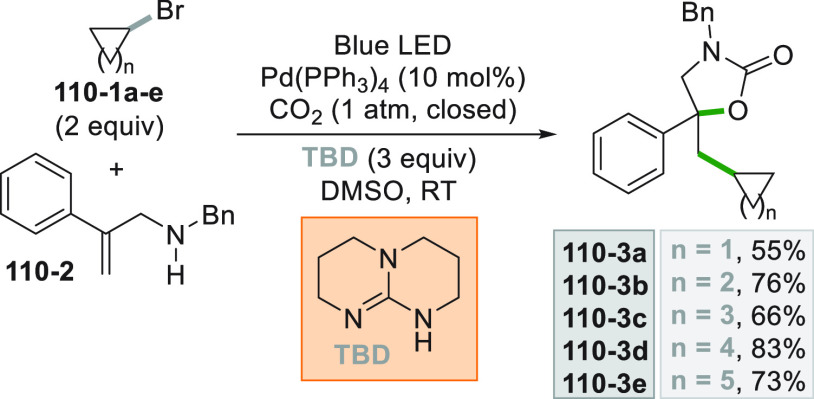
Three-Component Synthesis of 2-Oxazolidinones

It is also possible to incorporate more than one heteroatom in the ring starting from benzyl amine **110**–**2** and unactivated bromides **110–1a–e** (Scheme 110). Compound **110**–**2** incorporates CO_2_ (with the help of the base TBD), and the resulting carbamate underwent attack by an alkyl radical photogenerated by reaction of **110–1a–e** and an excited Pd^0^ photocatalyst (Pd(PPh_3_)_4_). Ring closing yielded valuable 2-oxazolidinones **110–3a–e** under very mild conditions and easy scalability.^355^

### 4.3 Six-Membered or Larger Rings

**Scheme 111:**
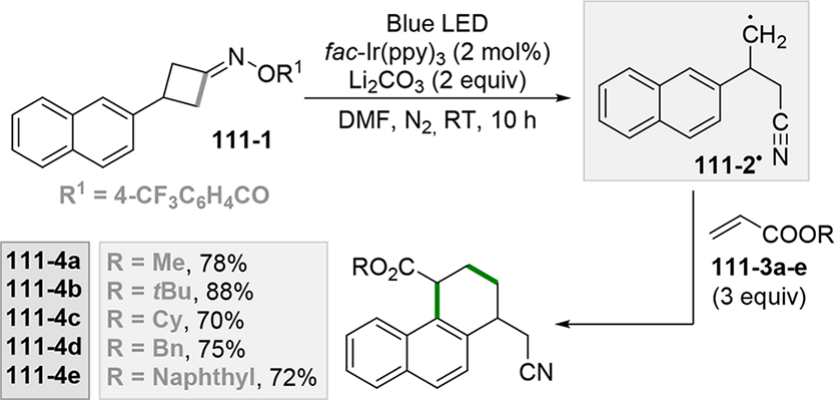
Photocatalyzed Preparation of 1,2,3,4-Tetrahydrophenanthrenes

Different approaches were devised to form a six-membered ring even cointaining heteroatoms. A cyclohexane ring was constructed by ring opening of an iminyl radical by Ir^III^-photocatalyzed reduction of a 3-phenyl *O*-acyl oxime (e.g., **111**–**1**) to give radical **111**–**2**^**•**^ that upon addition onto unsaturated esters **111–3a–e** and ensuing cyclization led to cyanoalkylated 1,2,3,4-tetrahydrophenanthrenes (**111–4a–e**, Scheme 111).^356^

Six-membered rings have been likewise obtained by ring expansion in cycloalkanone derivatives. This expansion was caused by the photocatalyzed decarboxylation of α-(ω-carboxyalkyl) β-keto esters, followed by an *exo-trig* cyclization of the resulting radical onto the carbonyl group that ultimately led to the one-carbon expanded cycloalkanones by β-cleavage.^357^

**Scheme 112:**
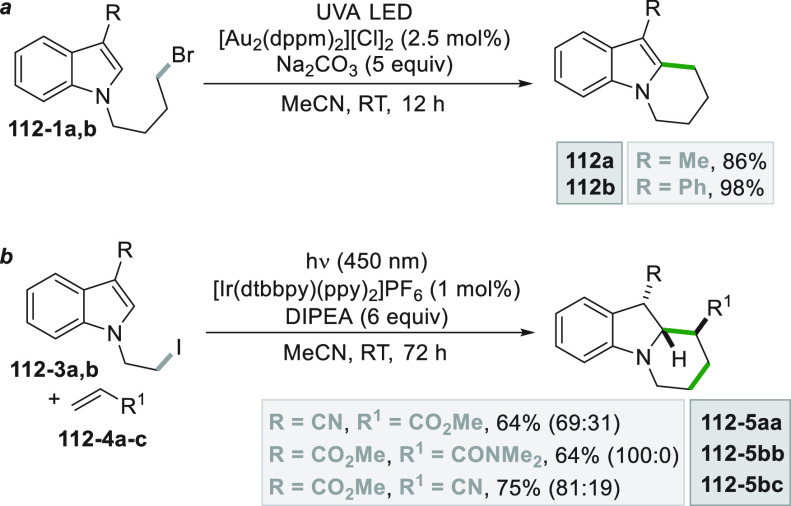
Intramolecular C–C Bond Formation in Indoles

Reduction of indoles having an unactivated haloalkane chain is a useful approach to construct a ring. As an example, bromo derivatives **112–1a,b** were reduced by a Au^I^ photocatalyst and radical cyclization onto the heteroaromatic ring afforded 6,7,8,9-tetrahydropyrido[1,2-*a*]indoles **112–2a,b** in excellent yield (Scheme 112a).^358^ Interestingly, changing the reaction conditions and starting from *N*-(2-iodoethyl)indoles **112–3a,b** in place of **112–1a,b** in the presence of Michael acceptors **112–4a–c** caused a dearomatizative tandem [4 + 2] cyclization to deliver tri- and tetracyclic benzindolizidines **112–5aa–bc** with high diastereoselectivity and yield (Scheme 112b).^359^

**Scheme 113:**
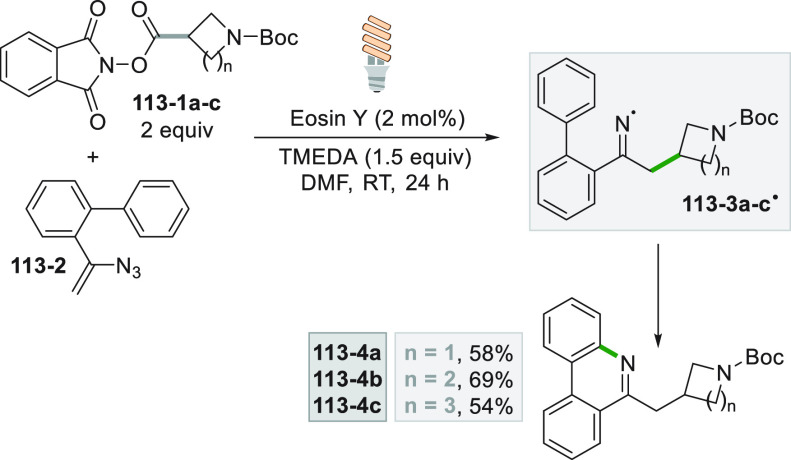
Photocatalyzed Synthesis of Functionalized Phenanthridines

The phenanthridine core is one of the elective scaffolds to be prepared by using a cyclization step induced by photogenerated alkyl radicals. Scheme 113 illustrated a representative case where an alkyl radical added onto a vinyl azide **113**–**2**, and after nitrogen loss the resulting iminyl radicals **113–3a-c**^**•**^ yielded phenanthridines **113–4a-c** by ring closure.^360^ The method has several advantages including metal-free conditions (a dye as a POC) an excellent functional group tolerance and a broad substrate scope.

**Scheme 114:**
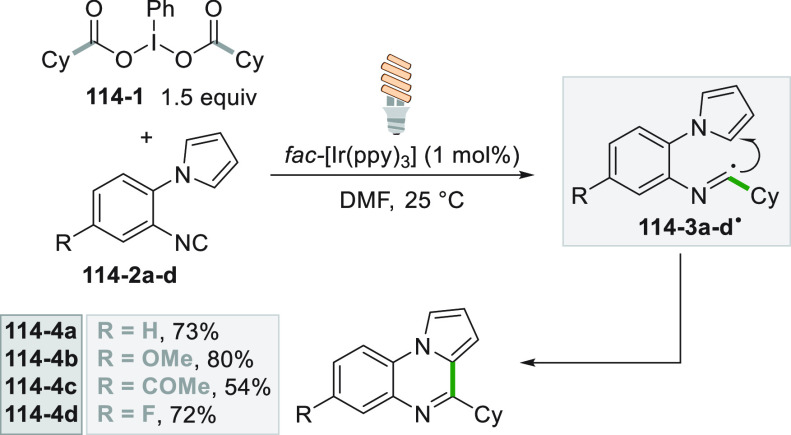
Photoredox Preparation of Pyrrolo[1,2-*a*]quinoxalines

An alternative way to prepare phenanthridines is by having recourse to photoredox gold catalysis employing bromoalkanes as alkyl radical source. In this case, radicals attack a biaryl isonitrile thus forming a sp^2^-hybridized radical that readily cyclizes upon the pendant arene.^361^ Aryl isocyanides (e.g., **114–2a–d**) were largely used for the construction of heterocycles such as pyrrolo[1,2-*a*]quinoxalines **114–4a–d**. Phenyliodine^III^ dicarboxylate **114**–**1** was used for the incorporation of the cyclohexyl group both in batch and flow under Ir^III^-photocatalyzed conditions (Scheme 114, see also Scheme 49).^362^

The photocatalyzed insertion of SO_2_ into an unactivated C(sp^3^)-H bond was designed to prepare 1,2-thiazine 1,1-dioxide derivatives under uncatalyzed conditions. In fact, visible light irradiation of the complex between an electron-poor *O*-aryl oxime and DABCO·(SO_2_)_2_ releases an iminyl radical that upon 1,5-HAT, SO_2_ incorporation and cyclization gave the hoped-for heterocycle in a satisfying yield.^363^

**Scheme 115:**
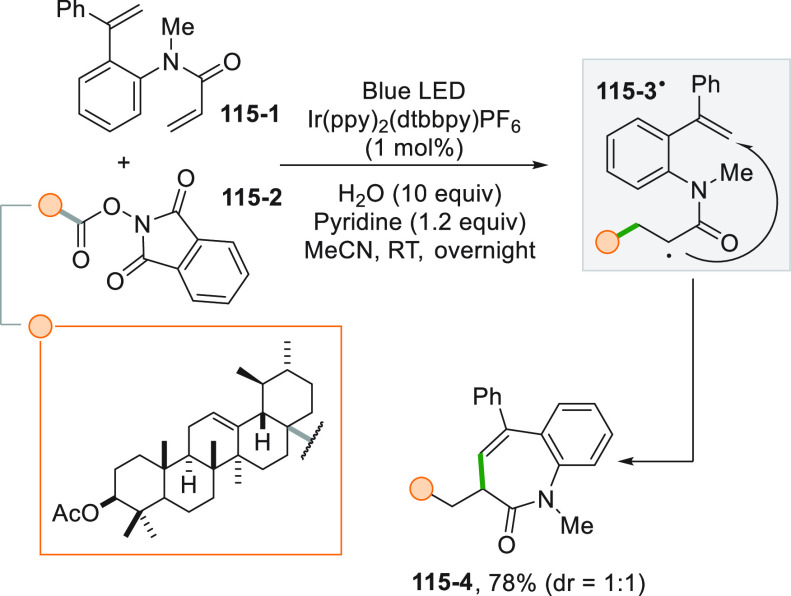
Late Stage Functionalization of Ursolic Acid

In rare instances a ring larger than six may be constructed. By using the approach depicted in Scheme 115, it was possible to pursue a late stage functionalization on ursolic acid (a compound having excellent pharmaceutical activity). Accordingly, the NHPI ester of ursolic acid acetate (**115**–**2**) underwent a radical addition cascade by a photocatalyzed reaction with acrylamide-tethered styrene (**115**–**1**) with the intermediacy of radical **115**–**3**^**•**^. As a result, the benzazepine unit was incorporated in the end compound **115**–**4** combining two privileged bioactive scaffolds.^364^

## 5 Conclusions and Outlook

This review provides a concise and up-to-date selection of modern methods to generate alkyl radicals via photochemistry and photocatalysis. The effort and interest of the chemical community in developing and applying these new methods is witnessed by the rapid increase in the number of articles devoted to this topic that appeared in the literature in the last two decades. Indeed, the rediscovery of photocatalysis and the renaissance of visible light-driven processes have contributed to elevate radical chemistry from the isolated (yet efficient) niche of the tyrannical organotin compounds to a vast plethora of methodologies that relies on more environmental benign compounds. The facile synthesis of the precursors necessary for these transformations, along with the readily available setups (a vast number of reactions can occur by simple irradiation with visible LEDs), made radical chemistry approachable, appointing the photon as the agent of this revolutionary democracy.

**Figure 4:**
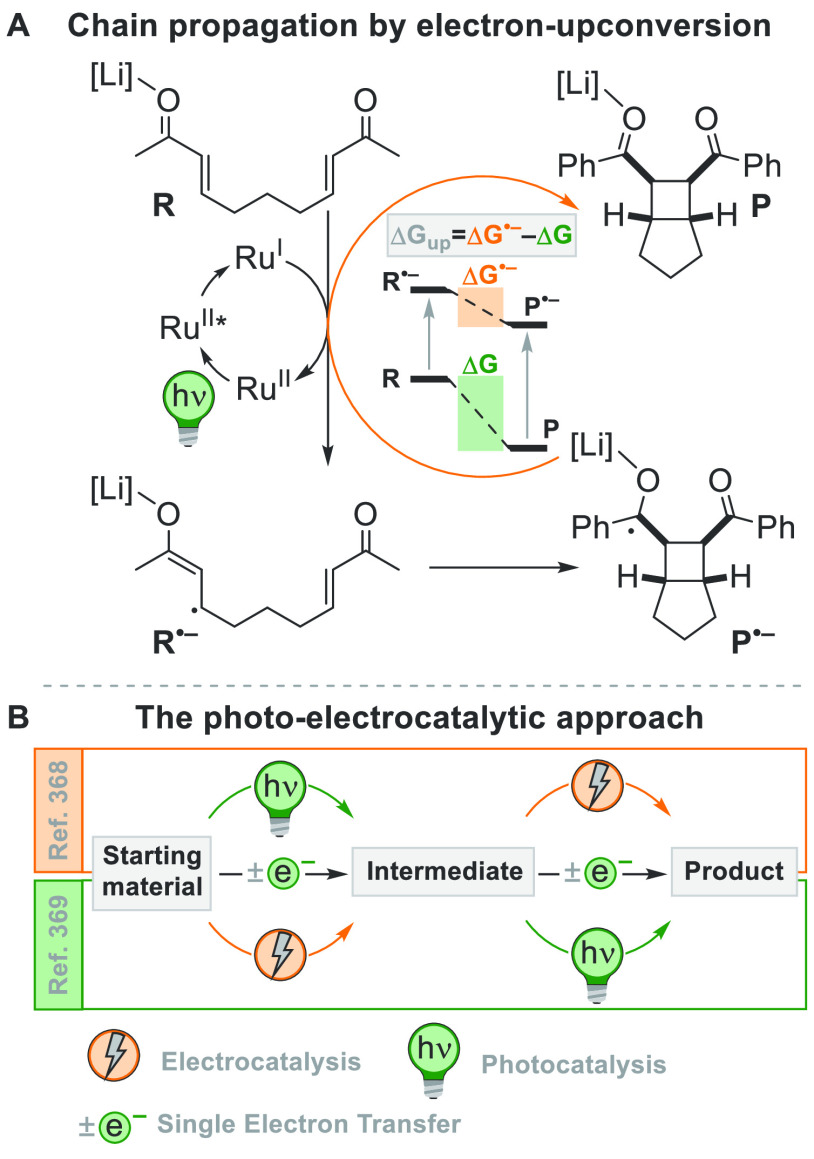
(A) Upconversion of the reducing power of the intermediates in a photocatalytic/photoinitiated cyclization. (B) Two pathways to employ the photoelectrocatalytic strategy: either promoting a single electron transfer with photocatalysis first and a second one with electrocatalysis or vice versa.

Photocatalysis has reached the stage of maturity; however, we are still far from the statement of Ciamician envisioning “industrial colonies without smoke [···] forests of glass tubes [···]; inside of these will take place the photochemical processes that hitherto have been the guarded secret of the plants, but that will have been mastered by human industry which will know how to make them bear even more abundant fruit than nature, for nature is not in a hurry and mankind is”.^365^ New practical methods and theoretical assumptions are needed to foster the revolution that has just started. A promising approach makes use of the upconversion of reductants to generate strongly reductive species, but the method was not applied so far to alkyl radicals.^366^ This phenomenon can be exploited, for example, if the reaction of a radical anion **R**^**•–**^ to give **P**^**•–**^ is less exoergonic (see the Δ*G*^**•–**^ value in Figure 4A) than its neutral counterpart (Δ*G*, referred to **R** → **P** conversion). The difference between these two free energies defines the upconversion energy (Δ*G*_up_ = Δ*G*^**•–**^ – Δ*G*). The high quantum yields associated with the transformation of **R** into **P** in Figure 4A (Φ = 44) were attributed to the presence of electrocatalytic cycles propagated by **P**^**•–**^, which is able to transfer an electron to the reactant, closing the catalytic cycle. This phenomenon is attributed to **P**^**•–**^ being a better reductant than **R**^**•–**^, due to the diminished conjugation (Figure 4A).

The novel approach granted by the merging of homogeneous photocatalysis with electrocatalysis (see Figure 4B) is surfacing as the new challenge in this constantly evolving topic.^367−369^

Joining the almost unlimited potential of these two interchangeable fields of research would open unprecedented scenarios in chemical synthesis, allowing one to tweak the reactivity of intermediates and excited state species at will, walking on the path carved by the institution of the photon democracy.

## Abbreviations

Acr^+^Mes: 9-mesitylene-10-methylacridinium
alkyl-DHPs: 4-alkyl-1,4-dihydropyridines
BDE: bond dissociation energies
BI-OAc: benziodoxole acetate
BI–OH: hydroxylbenziodoxole
4CzIPN: 1,2,3,5-tetrakis(carbazol-9-yl)-4,6-dicyanobenzene
DABSO: 1,4-diazabicyclo[2.2.2]octane bis(sulfur dioxide)
DBAD: di-*tert*-butylazodicarboxylate
DCN: 1,4-dicyanonaphthalene
DIAD: diisopropyl azodicarboxylate
DMAc: *N*,*N*-dimethylacetamide
DMAP: 4-dimethylaminopyridine
EY: Eosin Y
HAT: hydrogen atom transfer
*d*-HAT: direct hydrogen atom transfer reaction
*i*-HAT: indirect hydrogen atom transfer reaction
HDAC: histone deacetylase
HE: Hantzsch ester
Ir[dF(CF_3_)ppy]_2_(dtbbpy)^2+^: bis(2-(2,4-difluorophenyl)-5-trifluoromethylpyridine)(di*tert*-butylbipyridine)iridium
*fac*-Ir(ppy)_3_: *fac*-(tris(2,2′-phenylpyridine))iridium
[Ir(ppy)_2_(dtbbpy)]^+^: bis(2-phenylpyridine) (di-*tert*-butylbipyridine)iridium
LB: Lewis base
NFSI: *N*-fluorobenzenesulfonimide
NHPI: alkyl *N*-hydroxyphthalimide esters (Phth-ester)
PC: photocatalyst
PCET: proton-coupled electron transfer
PFBI–OH: perfluorohydroxylbenziodoxole
POC: photoorganocatalysts
RFTA: riboflavin tetraacetate
Ru^II^(bpy)_3_^2+^: tris(2,2′-bipyridine)ruthenium
SET: single electron transfer
TBACN: tetrabutylammonium cyanide
TBADT: tetrabutylammonium decatungstate
TBD: 1,5,7-triazabicyclo[4.4.0]dec-5-ene
TMHD: tetramethylheptanedione
TMSCN: trimethylsilyl cyanide
TTMSS: tris(trimethylsilyl)silane
XAT: halogen atom transfer reaction
